# Cyclooxygenase-1 deletion in 5 × FAD mice protects against microglia-induced neuroinflammation and mitigates cognitive impairment

**DOI:** 10.1186/s40035-025-00501-9

**Published:** 2025-08-22

**Authors:** Jie Wang, Hong Ni, Yu Wang, Luyao Wei, Hanqing Ding, Zhongzhao Guo, Hao Pan, Ying Yu, Jia Luo, Weidong Pan, Deheng Wang, Zun-Ji Ke

**Affiliations:** 1 Department of Chinese Medicine and Integrative Medicine, Shanghai Geriatric Medical Center, Zhongshan Hospital, Fudan University, Shanghai, 201104 China; 2 Department of Chinese Medicine and Integrative Medicine, Zhongshan Hospital, Fudan University, Shanghai, 200032 China; 3 School of Integrative Medicine, Shanghai University of Traditional Chinese Medicine, Shanghai, 201203 China; 4 Engineering Research Center of Traditional Chinese Medicine Intelligent Rehabilitation, Shanghai University of Traditional Chinese Medicine, Shanghai, 201203 China; 5 Department of Neurology, Shuguang Hospital Affiliated to Shanghai University of Traditional Chinese Medicine, Shanghai, 201203 China; 6 Department of Pharmacology and Tianjin Key Laboratory of Inflammatory Biology, Tianjin Medical University, Tianjin, 300070 China; 7 Department of Pathology, University of Iowa Carver College of Medicine, 51 Newton Road, Iowa City, IA 52242 USA; 8 Department of Rehabilitation, Yueyang Hospital of Integrated Traditional Chinese and Western Medicine, Shanghai, 200437 China; 9 Department of Neurosurgery, Shuguang Hospital Affiliated to Shanghai University of Traditional Chinese Medicine, Shanghai, 201203 China

**Keywords:** Alzheimer’s disease, Cyclooxygenase-1, Neuroinflammation, Microglia, Cognitive impairment, NLRP3 inflammasome

## Abstract

**Background:**

Alzheimer's disease (AD) is a neurodegenerative disease with major symptoms including memory and learning deficits. Neuroinflammation associated with reactive microglia promotes AD progression. These reactive microglia secrete prostaglandins, which are synthesized through the enzymatic activity of cyclooxygenase (COX)-1 and COX-2. Here, we aimed to elucidate the specific mechanisms of COX1 in AD pathogenesis and its interactions with neuroinflammatory processes.

**Methods:**

We conducted backcrossing between COX-1 knockout (KO) and 5 × FAD mice to evaluate the effect of COX-1 deficiency on neuroinflammation. In addition, single-cell sequencing and microarray datasets from public databases and ingenuity pathway analysis in vitro were employed to explore gene expression profiles in the brains of AD mice.

**Results:**

We identified a significant upregulation of COX-1 in 5 × FAD mice, with expression specifically localized to microglia in an age-dependent manner. Additionally, COX-1 KO alleviated neuroinflammation and accumulation of Aβ plaques, subsequently improving cognitive behavior in 5 × FAD mice. Moreover, microglia exhibited an amoeboid morphology in 5 × FAD mice, whereas in age-matched 5 × FAD/COX-1 KO mice, microglia had a ramified appearance. Additionally, our study demonstrated a pharmacological approach that inhibits the prostaglandin E2 (PGE2)/EP2 receptors via inhibition of the cAMP-PKA-NFκB-p65 pathway and NLRP3 inflammasome activation, producing similar beneficial effects as observed in COX-1 KO mice.

**Conclusion:**

Our findings indicate that targeting the COX-1/PGE2/EP2 signaling pathway may alleviate neuroinflammation and impede AD progression. Moreover, the EP2 receptor presents a promising pharmacological target for mitigating the pathological effects associated with COX-1 activity in AD patients.

**Supplementary Information:**

The online version contains supplementary material available at 10.1186/s40035-025-00501-9.

## Introduction

The incidence of Alzheimer's disease (AD) was around 55 million people globally in 2019 and this figure is projected to increase to 139 million by 2050 [1]. With this rise, AD will affect approximately 5%–8% of people aged over 60 years, posing significant challenges to public health. AD is pathologically characterized by the formation of senile plaques composed of amyloid-beta (Aβ) peptides resulting from aberrant cleavage of amyloid precursor protein (APP), and neurofibrillary tangles which arise from hyperphosphorylation of tau protein [2]. Aducanumab was designed to directly target aggregated oligomeric and neurofibrillary forms of Aβ [3], yet its efficacy remains debatable [4]. In this context, addressing modifiable risk factors presents a promising strategy for AD prevention.

Neuroinflammation is increasingly recognized as a significant contributor to AD progression [5]. Aspirin is a nonselective inhibitor of cyclooxygenase (COX) enzyme, inhibiting the synthesis of prostaglandins (PGs) and reducing pro-inflammatory cytokines. Aspirin is widely used in anti-inflammatory and antiplatelet treatments [6]. It can hinder AD progression by facilitating Aβ peptide clearance, decreasing tau phosphorylation, and improving synaptic plasticity [7]. In addition, chronic low-dose aspirin reduces Aβ plaques in 5 × FAD mice by inducing lysosomal biogenesis through activation of peroxisome proliferator-activated receptor [8, 9]. However, the specific mechanism by which aspirin inhibits neuroinflammation through targeting COX-1 or COX-2 remains unclear.

Overactivation of microglia, in conjunction with inflammatory mediators, is critically involved in the neuroinflammatory process [10]. Microglial processes are highly dynamic, and their activation is typically characterized by progressive morphological changes from a highly branched (ramified) state to an activated amoeboid state, along with upregulation of surface molecules [11]. Microglia are recognized to possess diverse reactive phenotypes associated with stages of AD. During early stages, microglia adopt a homeostatic or disease-associated microglia (DAM)-like phenotype with phagocytic activity (e.g., expression of triggering receptor expressed on myeloid cells 2 [TREM2] and secretion of arginase-1 [Arg-1]), which are stimulated by anti-inflammatory cytokines such as interleukin-4 (IL-4) and transforming growth factor-β. This stimulation leads to the secretion of various factors like Arg-1, which possesses migratory and phagocytic properties that facilitate microglial migration to injury sites and subsequent removal of accumulated Aβ [12–14]. With disease progression, microglia transit to a hyperinflammatory state, marked by increased expression of NLR family pyrin domain containing 3 (NLRP3), interleukin-1β (IL-1β) and CD68, which exacerbates the inflammatory cascade and eventually results in neuronal death and chronic neuroinflammation [13–15]. Consequently, elucidating the pathways underlying the alteration of microglial states during AD progression is crucial for the development of therapeutic strategies.

Moreover, COX-1 expression is elevated in neurodegenerative disorders [16] and is predominantly localized within microglia, where it contributes to the pro-inflammatory processes in AD [17, 18]. Clearing COX-1 can reduce Aβ-induced neuroinflammation and inhibit proinflammatory factor secretion [19]. A high-profile clinical trial showed that the selective COX-1 inhibitor piroxicam can slow the progression of AD [20]. Unlike COX-1, COX-2 is mainly expressed in neurons in many neurodegenerative diseases [21], including Parkinson’s disease [22, 23] and amyotrophic lateral sclerosis [24]. COX-2 knockout even exacerbates neuroinflammation in lipopolysaccharide (LPS)-treated mice [25]. COX-1 plays a significant role in the neuroinflammatory processes associated with AD. Inhibiting COX-1 can reduce memory impairment by suppressing inflammation [26]. Upregulation of COX-1 leads to increased production of PGs including prostaglandin E2 (PGE_2_), which exacerbates Aβ aggregation, synaptic degeneration, neuronal apoptosis, and cognitive decline in AD models [17]. Notably, PGE_2_ binds to EP2/EP4 receptors and activates the cyclic adenosine monophosphate (cAMP)/protein kinase A (PKA)/nuclear factor kappa-light-chain-enhancer of activated B cells (NFκB) signaling cascade, driving NLRP3 inflammasome assembly and subsequent IL-1β secretion [27, 28]. Interestingly, targeting COX-1 may mitigate memory deficits induced by hypobaric hypoxia through attenuating the NLRP3 inflammasome-mediated neuroinflammatory pathways, thereby preserving neurogenic processes [29]. These findings imply that selective COX-1 inhibitors may reduce neuroinflammation. Nevertheless, the extent to which COX-1 and the downstream NLRP3 inflammasome contribute to microglia-induced neuroinflammation in AD remains largely unexplored.

In this study, we aimed to examine the potential role of COX-1 in AD progression by crossing COX-1 KO mice with 5 × FAD mice, a well-built murine model of AD [30]. Particularly, the impact of COX-1 deletion on microglia-induced neuroinflammation and the underlying mechanisms were investigated.

## Materials and methods

### Single-cell and microarray data sources

Single-cell data were obtained from ssREAD (https://bmblx.bmi.osumc.edu/ssread/), a user-friendly web server with compiled single-cell data that provides extensive, detailed analyses and interpretations of the results [31]. Data of aging persons were obtained from Mathys H et al. (public data ID: syn18485175) [32], and data of age-matched AD patients were obtained from Zhou Y et al. (syn21125841) [33]. Data of 7-month-old wild type (WT) mice were obtained from Keren-Shaul H et al. (public data ID: GSE98969) [34]. Data from 7-month-old AD mice clusters were obtained from Zhou Y et al. (public data ID: GSE140510) [33].

The original microarray datasets were obtained from the Gene Expression Omnibus (GEO) database (https://www.ncbi.nlm.nih.gov/geo/) (Accession: GSE33000 [35]). Raw microarray CEL files were preprocessed using the R package affy (version 1.76.0) with the Robust Multi-array Average normalization method, followed by log2(x + 1) transformation to stabilize expression variance [36]. For the ROSMAP (Religious Orders Study and Memory and Aging Project, public data ID: syn3157322) cohort, we employed the Wilcoxon rank-sum test given the large-scale sample size and non-normally distributed data between AD, mild cognitive impairment (MCI), and cognitively normal (CN) groups. Disease stratification was performed using standardized neuropathological and clinical criteria according to COGDx and Braak Staging diagnosis classification [37].

### Animals

5 × FAD mice are APP/PS1 double transgenic mice that express 695-amino-acid isoform of the human APP (APP695) containing the Swedish (K670N/M671L), Florida (I716V), and London (V717I) mutations, as well as human PSEN1 with M146L/L286V mutations. Heterozygous mice were generated by crossing 5 × FAD mice with C57BL/6 mice. Both were obtained from Model Animal Research Center of Nanjing University (Nanjing, China). COX-1 KO mice were kindly provided by the Chinese Academy of Sciences and were bred in the Experimental Animal Center of Shanghai University of Traditional Chinese Medicine. 5 × FAD/COX-1 KO transgenic mice were generated by crossing heterozygous 5 × FAD (±) mice with homozygous COX-1^−/−^ mice to obtain 5 × FAD (±)/COX-1 (±) mice, followed by subsequent crossings of 5 × FAD (±)/COX-1 (±) mice to generate 5 × FAD (±)/COX-1^−/−^ mice (referred to as 5 × FAD/COX-1 KO mice). In 5 × FAD mice, amyloid deposition and gliosis begin to appear at 2 months and increase progressively in the subiculum and deep cortical layers. They show impaired memory in the Y-maze, deficits in fear conditioning, as well as learning and memory deficits in the Morris water maze from 6 months of age [38, 39]. In this study, male mice were selected to minimize variability associated with hormonal fluctuations during the estrous cycle in females, which can influence neuroinflammatory responses and behavioral outcomes [40, 41]. Therefore, male C57BL/6, 5 × FAD, and 5 × FAD/COX-1KO mice aged 3 months, 6 months and 9 months (*n* = 10, at each age) were used in the experiments. 8-month-old 5 × FAD/COX-1 KO mice were treated with ONO-AE1-259 for 1 month (*n* = 5). All mice were hosted in an environment at temperature of 22 ± 2 °C and humidity of 55% ± 10% under a 12-h light/dark cycle (light on 08:00–20:00) with ad libitum access to sterilized drinking water and standard food. Before experiments, all mice were allowed for 7 days of acclimation. All experimental protocols were approved by the Animals Research Ethics Committee of Shanghai University of Chinese Medicine (No. PZSHUTCM200821019). Experimenters conducting behavioral tests, histological analyses, and molecular assays were blinded to mouse genotypes. Mouse genotyping methods are provided in additional file 1.

### Brain tissue harvest

Tissue preparation was conducted following previously established protocols with slight modifications [42]. After behavioral experiments, brain samples were collected. Mice were anesthetized by intraperitoneal injection of 1% sodium pentobarbital (10 mg/kg) (Aladdin, Shanghai, China), followed by cardiac perfusion. Brains were carefully harvested and bisected. Hippocampi were manually microdissected from coronal brain sections (bregma − 1.34 to − 3.88 mm) using fine forceps under stereomicroscopic guidance (Leica M80), following protocols from Paxinos and Franklin. The left hemisphere was then drop-fixed in 4% paraformaldehyde (PFA) for 24 h, and then transferred to a 30% sucrose solution for dehydration. Once the brain sank, it was sliced into 30 μm sections using a freezing microtome. Five brain slices were randomly selected between bregma − 1.46 and − 3.08 for quantification. The right hemisphere of the brain was flash-frozen by liquid nitrogen and stored at − 80 °C for mRNA and protein extraction. The methodological sequence is provided in Fig. 2a.

### Measurement of PGs by high-performance liquid chromatography (HPLC)/mass spectrometry (MS)

The PGs profiles in mouse hippocampus were measured as previously reported [43]. The hippocampus was dissected and homogenized in 1 mL PBS containing 100 μmol/L indomethacin. The supernatant was collected and spiked immediately with 5 ng PGD_2_-d4 (Cayman Chemical, Ann Arbor, MI), PGE_2_-d4 (Cayman Chemical), 6-keto Prostaglandin F_1α_-d4 (Cayman Chemical,), PGF_2α_-d4 (Cayman Chemical), and TxB_2_-d4 (Cayman Chemical), then purified by solid phase extraction using StrataX C18 cartridges (Phenomenex, Torrance, CA). The solid phase extraction cartridge was conditioned with 1 mL of acetonitrile and equilibrated with 1 mL of water. The sample was applied to the cartridge, which was then washed with 1 mL of 5% acetonitrile in water and dried with vacuum for 15 min. The analyte and internal standards were eluted from the cartridge using 1 mL of 5% acetonitrile in ethyl acetate. The eluate was collected and dried under a gentle stream of nitrogen. The resulting residue was reconstituted in 200 μL of 5% acetonitrile in water and filtered by centrifugation using 0.2 μm Nylon Microspin filters (Alltech Associates, Columbia, MD), then quantitated by LC/MS/MS as described previously [44].

### Hemostasis model

A mouse tail bleeding assay was performed as described [45]. Mice were anesthetized with pentobarbital (100 mg/kg, i.p.) and placed prone on a warming pad from which the tail protruded. The mouse tail was transected at 3 mm from the tip and immediately immersed into 10 mL saline at 37 °C. The bleeding time was determined as the time from tail transection to the cessation of blood flow for more than 2 min.

### Microglial isolation

Microglial isolation was performed as described previously [46]. Briefly, 9-month-old male C57BL/6 J and 5 × FAD mice were perfused transcardially with heparinized Ringer's solution under deep anesthesia. Brains were removed, meninges carefully dissected, and tissue minced in papain-based enzymatic solution (containing 116 mmol/L NaCl, 5.4 mmol/L KCl, 26 mmol/L NaHCO₃, 1 mmol/L NaH₂PO₄, 1.5 mmol/L CaCl₂, 1 mmol/L MgSO₄, 0.5 mmol/L EDTA, 25 mmol/L glucose, 1 mmol/L cysteine, and 20 U/mL papain). After 90-min digestion at 37 °C/5% CO₂, the reaction was quenched with 20% fetal bovine serum (FBS)-Hank's Balanced Salt Solution (HBSS). The cell suspension was sequentially processed by DNase I treatment (0.5 mg/mL, 5 min), mechanical dissociation using fire-polished pipettes, and 70-μm filtration. For myelin removal, cells underwent Percoll density centrifugation: a discontinuous gradient of 20% SIP/HBSS overlaid with pure HBSS was centrifuged at 200× *g* (20 min). The pellet containing glial cells was resuspended in Dulbecco’s modified Eagle’s medium (DMEM)/F12 medium supplemented with 10% FBS, penicillin/streptomycin, and 5 ng/mL granulocyte–macrophage colony-stimulating factor (GM-CSF), then plated on poly-*L*-lysine-coated T75 flasks. Cultures were maintained at 37 °C/5% CO₂ with biweekly medium change. Floating microglia were harvested from confluent cultures (2 weeks) by collecting supernatant fractions without mechanical agitation. Cells were centrifuged (200× *g*, 7 min), replated in GM-CSF-free medium, and used for experiments after ≥ 3 days of adaptation. Cells were collected for subsequent experiments.

### EP2 receptor agonist treatment

ONO-AE1-259, which exhibits a high affinity for EP2 receptors with a *K*i value of 3 nmol/L, has been characterized in structure and activity [47, 48]. In this study, 8-month-old 5 × FAD/COX-1 KO mice were subcutaneously injected with 50 μL of a saline solution containing ONO-AE1-259 (OnoPharmaceutical Co., Ltd., Osaka, Japan) (10 μg/kg, dissolved in 1% DMSO in sterile 0.9% Nacl), as previously described [49], on days 1–5 each week for 4 weeks.

### Hematoxylin–eosin (HE) staining

The brain, liver, stomach, and kidney tissues were fixed in 4% PFA, followed by gradient ethanol dehydration. Paraffin sections were stained with HE (G1120, Solarbio, Beijing, China). All sections were observed under a microscope (Axio Imager, Zeiss, Oberkochen, Germany).

### Evans Blue (EB) staining

The blood–brain barrier (BBB) permeability was evaluated using EB extravasation assay following established protocols [50]. In brief, mice received intravenous injection of 2% EB (E8010, Solarbio) solution in 0.9% saline (4 mL/kg body weight). One hour later, animals were deeply anesthetized with sodium pentobarbital (150 mg/kg), followed by right ventricular blood collection. Complete transcardial perfusion with 0.9% saline was performed to eliminate intravascular dye prior to brain harvest. Brain tissues were homogenized (1:10 *w*/*v*) in phosphate buffered saline (PBS) and mixed with 50% trichloroacetic acid to precipitate proteins. After centrifugation (10,000× *g*, 20 min, 4 °C), the supernatant absorbance at 620 nm was measured using a microplate reader (SpectraMax® M5, Molecular Devices, San Jose, CA). EB concentrations were quantified against a standard curve (0.1–100 μg/mL) and normalized to both brain tissue mass and serum EB levels to account for individual circulatory variations.

### Immunohistochemistry (IHC) staining

IHC staining was conducted following previous protocols with slight modification [42]. Brain slices were rinsed three times in PBS, then placed in a 6-well plate with a PBS, methanol, and 30% hydrogen peroxide mixture (5:4:1 ratio) and shaken for 30 min. The plate was subsequently placed on a shaker for 30 min. The brain slices were then transferred to 0.1% Triton-X100 in PBS buffer in 6-well plates and shaken for an additional 30 min. Further blocking was performed in a PBS buffer solution containing 1% BSA for 2 h. The brain slices were incubated with rabbit anti-Iba-1 antibody (Cat. No. 019-19741, Wako, Osaka, Japan; 1:1000) overnight at 4 °C. On the second day, the slices were incubated with horseradish peroxidase (HRP)-conjugated goat anti-rabbit secondary antibody (Cat. No. 32460, Life Technologies, Carlsbad, CA; 1:1000). The chromogenic development was facilitated using a 3,3’-Diaminobenzidine (DAB, Sigma, St. Louis, MO) solution. Upon completion of the color development process, the brain sections were rinsed three times with PBS and mounted onto adhesive slides for air dry. Subsequently, the sections underwent a series of dehydration and degreasing steps, and were ultimately sealed with neutral resin prior to microscopic analysis (Axio Imager, Zeiss, Oberkochen, Germany). Iba1^+^ microglia density was quantified in five brain slices (selected between bregma − 1.46 and − 3.08 mm). For each slice, three non-overlapping 100 × 100 µm^2^ fields in the cortex, CA1, and dentate gyrus were imaged at 20× magnification. Iba1^+^ cells were manually counted using ImageJ and normalized to area (cells/mm^2^). The numbers of positive cells from a total of 15 observation areas were averaged for each mouse.

### Immunofluorescence (IF) staining

Brain sections were blocked with 5% BSA in Tris-buffered saline with Tween 20 (TBST) for 10 min. Then they were incubated with the following primary antibodies overnight at 4 °C: rabbit anti-Iba-1 (Cat. No. 019-19741, Wako, Osaka, Japan; 1:500) in combination with either goat anti-COX-1 (Cat. No. NB100-867, Novus, Centennial, CO; 1:200), mouse anti-CD68 (Cat. No. YM3050, Immunoway, Salem, MA; 1:200), or rat anti-Arg-1 (Cat. No. SC-271430, Santa Cruz, Dallas, TX; 1:200), or mouse anti-6E10 (Cat. No. 803004, Biolegend, San Diego, CA; 1:1000). Additionally, mouse anti-Iba-1 (Cat. No. SC-32725, Santa Cruz; 1:100) was mixed with rabbit anti-EP2 (Cat. No. ab167171, Abcam, Cambridge, UK; 1:250), and mouse anti-COX1 (Cat. No. sc-19998, Santa Cruz) was combined with either rabbit anti-glial fibrillary acidic protein (GFAP) (Cat. No. 16825-1-AP, Proteintech, Rosemont, IL; 1:250) or rabbit anti-CD31 (endothelial marker, Cat. No. ab222783, Abcam; 1:100) or rabbit anti-NeuN (neuronal marker, Cat. No. MAB377, Sigma; 1:200). Tissue sections were washed three times with PBS and subsequently incubated in the dark with secondary antibodies, including donkey anti-rabbit Alexa Fluor 555 (Cat. No. A31572, Life Technologies, Carlsbad, CA; 1:1000) combined with either donkey anti-goat Alexa Fluor 488 (Cat. No. A-21081, Life Technologies; 1:1000), goat anti-rat Alexa Fluor 488 (Cat. No. A11006, Life Technologies; 1:1000), or goat anti-mouse Alexa Fluor 488 (Cat. No. A21151, Life Technologies; 1:1000). Additionally, donkey anti-rabbit Alexa Fluor 488 (Cat. No. A21206, Life Technologies; 1:1000) was used in combination with goat anti-mouse Alexa Fluor 555 (Cat. No. A32727, Life Technologies; 1:1000) for 2 h at RT. After three washes with PBS, nuclei were counterstained with DAPI (Cat. No. C1006, Beyotime, China). The sections were mounted by fluorescent mounting medium (Cat. No. P0126, Beyotime, Shanghai, China), and observed with a confocal laser microscope (Leica TCS SP2, Germany), using a 20 × air objective with a numerical aperture of 0.5.

Morphometric analysis was performed offline with Fiji ImageJ software (W. Rasband, National Institutes of Health). Antibody areas were determined automatically by creating outline masks based on brightness thresholds from maximal projected confocal images [51]. Fluorescence intensity was quantified using ImageJ (Fiji) after background subtraction and ROI selection, then normalized according to cell-specific markers [52]. Meanwhile, co-expression (e.g., Iba1^+^CD68^+^) was quantified using the Coloc2 plugin, with a Pearson’s coefficient ≥ 0.5 defining positivity. Data were expressed as the percentage of double-positive cells relative to total Iba1^+^ cells per field [53]. For quantification of plaque-associated microglia, Aβ plaques were identified by 6E10 immunostaining (green channel). The Auto Thresholding plugin in Fiji was used to segment plaques with a minimum area of 20 µm^2^. A 20-µm radial buffer zone around each plaque’s centroid was defined as the plaque-associated region. Iba1^+^ cells with > 50% of their soma or processes within this zone were classified as plaque-associated. Plaque-associated Iba1^+^ cells were counted per plaque and normalized to plaque area (cells/mm^2^) [54].

### Skeleton and Sholl analysis of microglia

Skeleton and Sholl analysis of microglia was performed based on published protocols [55]. Briefly, the image was converted to 8-bit grayscale to optimize the visualization of positive staining. Brightness and contrast were adjusted to improve visualization of microglial processes if the image was too dim. Contrast was enhanced by the unsharp mask filter, followed by a despeckle procedure to remove noise. Finally, the image was converted to binary using the Image | Adjust | Threshold option. The “despeckle”, “close”, and “remove outlier” functions were applied. The image was then skeletonized using FIJI software and AnalyzeSkeleton plugins. At least 3 to 5 images in the cortex region with a minimum of 15 microglia per mouse were analyzed. As for Sholl Analysis, branching complexity was quantified by counting intersections of microglial processes within concentric circles at increasing radii (3 µm) from the soma using the ImageJ (Fiji) software [52].

### Thioflavin S (Thio-S) staining

Thio-S staining was performed according to our previously published protocols [42]. Brain slices were taken from the cryopreservation solution, rinsed with PBS three times, and mounted on 1% gelatin-coated slides to air dry. Then, 0.05 g of Thio-S powder was dissolved in 1 mL ddH_2_O, and 0.5 mL was mixed with 49.5 mL of 50% alcohol. The dried slices were placed in this solution in a light-proof shaker for 8 min, washed twice with 80% alcohol for about 10 s each, followed by 4–5 washes with ddH_2_O. They were then shaken in PBS at 4 °C for 30 min, washed 4–5 times with ddH_2_O, blocked with a fluorescence sealing agent, and observed under a fluorescence microscope (Zeiss, Oberkochen, Germany). Five brain slices from each mouse (5 mice in each group) were analyzed. The percentage of positive signals in the selected area relative to the total area was analyzed with the Image J software.

### Open field test (OFT)

OFT was performed following established protocols in four 50 × 50 × 40 cm^3^ white opaque chambers. Mice were placed in the center, and their movement speed, distance traveled, central area crossings, and time spent in the center were recorded over 5 min [42]. After each test, the chambers were cleaned with 75% alcohol to remove odors and fecal matter. Data were analyzed using the Noldus EthoVision XT software (Noldus Information Technology, Wageningen, Netherlands).

### Novel object recognition (NOR) test

Two identical objects were placed in the center of the test box. Each mouse was placed in the box and its interactions with the objects were recorded for 5 min. After an hour, one object was replaced with a new one with a different shape, color and material, and mouse behavior was recorded for another 5 min. The mouse ability to recognize the new object was also evaluated 24 h later. The discrimination ratio was calculated as follows: discrimination ratio = *T*_new_/(*T*_new_ + *T*_familiar_) × 100%. Data were analyzed with the Noldus Ethovision XT software.

### Morris water maze (MWM)

MWM was performed as previously described [42]. Mice were trained to localize the hidden platform on day 1 to day 5 (three trials per day with 1-h intervals). On day 6, a probe trial was performed to test the spatial memory of mice. Their swimming paths were tracked using the Noldus EthoVision XT system.

### Alanine aminotransferase (ALT), aspartate aminotransferase (AST), urea, and creatinine assays

Following a 10–12 h overnight fasting, venous blood was collected between 09:00 and 11:00, processed within 1 h of collection, and analyzed for AST and ALT (for liver injury) as well as urea and creatinine (for renal filtration capacity) using a Beckman Coulter AU5800 automated analyzer according to manufacturer’s specifications.

### Aβ ELISA assay

Cortex was isolated and lysed in radioimmunoprecipitation (RIPA) buffer (Cat. No. P0038, Beyotime) supplemented with protease inhibitors (Cat. No. P1005, Beyotime). Protein levels were quantified by BCA method (Cat. No. P00100S, Beyotime) and diluted to 1 μg/mL. Aβ40 (Cat. No. AB-B208711, Abmart) and Aβ42 (Cat. No. AB-B21488, Abmart) levels were quantified using ELISA kits according to the manufacturer’s instructions.

### Cell culture and treatment

BV2 cells, a murine microglia cell line, were cultured in DMEM with 10% FBS and 1% penicillin–streptomycin (Invitrogen, Carlsbad, CA) in 6-well plates (250,000 cells/well) with 5% humidified CO_2_ and 95% air at 37 °C, as previously described [56, 57]. Upon reaching 70%–80% confluency, cells were treated with the selective COX-1 inhibitor SC-560 (6 μmol/L; Abcam) for 1 h. Then the culture medium was collected for quantitative real-time PCR (qRT-PCR) and western blotting. H89 dihydrochloride hydrate (25 μmol/L; Selleck, Houston, TX), an inhibitor of PKA, was added for 2 h in the cell culture medium followed by PGE_2_ treatment (1 μmol/L; Selleck) for 4 h. QNZ (20 μmol/L; Selleck), an inhibitor of NF-κB, was added for 30 min in the cell culture followed by PGE_2_ treatment (1 μmol/L; Selleck) for 4 h. TG4-155 (1 μmol/L, Selleck), an inhibitor of EP2, was added for 1 h in the cell culture medium followed by PGE_2_ treatment (1 μmol/L; Selleck) for 4 h [58].

### IL-1β ELISA assay

IL-1β levels were determined using the mouse IL-1β ELISA Kit (R&D Systems, PMLB00C) according to the manufacturer’s protocol [59]. Brain tissues were homogenized in RIPA buffer containing 1 × protease inhibitor cocktail at a ratio of 1:10 (*w*/*v*). Homogenates were centrifuged at 12,000× *g* for 15 min at 4 °C, and supernatant was collected. Total protein concentration was determined using a BCA Protein Assay Kit. BV-2 murine microglial cells were cultured in DMEM medium supplemented with 10% FBS and 1% penicillin/streptomycin. Cells were treated as discussed above and supernatants were collected after centrifugation (300× *g*, 5 min) to remove debris. Supernatants were stored at −80 °C until analysis.

### Immunofluorescence on BV2 cells

BV2 cells treated with PGE_2_ or PGE_2_ + H89 (an inhibitor of PKA) were grown on coverslips in 6-well plates to about 60% confluency. After 24 h of incubation, cells were fixed with 4% PFA for 30 min, washed with PBS three times, and incubated with rabbit anti-NF-κB p65 antibody (Cat. No. 8242S, CST, Danvers, MA; 1:200) at 4 °C overnight. The coverslips were then gently rinsed three times with PBS and incubated in the dark with donkey anti-rabbit Alexa Flour 555 (Cat. No. A31572, Life Technologies; 1:1000) for 2 h at RT. Subsequently, nuclei were counterstained with DAPI (Cat. No. C1006, Beyotime), and mounted with fluorescent mounting medium (Cat. No. P0126, Beyotime). Photographs were taken by a fluorescence microscope (LSM780, Zeiss, Oberkochen, Germany).

### Transfection of siRNA

The siRNAs for NF-κB p50 and p65 were designed and synthesized by Shanghai Genomeditech Co., Ltd. The target sequence of *p50* was 5’-CGCCAUCUAUGAUAGCAAA-3’ and that of *p65* was 5’-CUGAAGCUAUAACUCGCCU-3’. Briefly, 5 μL siRNA stock solution was diluted in 250 μL Opti-MEM medium, then 5 μL of lipofectamine 2000 (Lipo2000) transfection reagent (Thermo Fisher Scientific) was diluted with 250 μL Opti-MEM medium and incubated for 5 min at RT. Next, the aforementioned solutions were mixed and incubated for 18 min to form a transfection complex. Following this, 1500 μL of Opti-MEM medium and 500 μL of the transfection complex were added to cell cultures and incubated for 6 h. Then the medium was replaced by 2 mL of DMEM medium and cultured at 37 °C for 24 h.

### cAMP ELISA

cAMP level was quantified by a direct competitive ELISA kit according to the manufacturer’s instructions (Cat. No. ab65355, Abcam). Briefly, frozen tissues were homogenized on ice in 0.1 mol/L HCl at a ratio of 1:5 (*w*/*v*) and centrifuged at 12,000 × *g* for 5 min. The supernatant was collected for ELISA analysis. Color developed was read at OD 450 nm using an ELx800 plate reader (BioTek, Santa Clara, CA).

### Western blot analysis

Proteins were extracted from hippocampus, BV2 cells or isolated microglia cells from mice. Protein concentrations were determined by a BCA Protein Assay kit (Thermo Fisher Scientific). Ten micrograms of protein were separated by 12.5% SDS-PAGE (Yeasen Biotech, Shanghai, China), and transferred to nitrocellulose membranes (Yeasen Biotech). The membranes were blocked for 1 h with 5% BSA in the TBST buffer (10 mmol/L Tris, pH 7.5, 100 mmol/L NaCl, and 0.1% Tween 20) and then washed three times with TBST. Then, the blots were incubated overnight at 4 °C with specific primary antibodies (1:1000 dilution in 5% nonfat milk in the TBST buffer) of goat anti-COX-1 (Cat. No. NB100-867, Novus), mouse anti-COX-1 (Cat. No. sc-19998, Santa Cruz), rabbit anti-COX-2 (Cat. No. T588524S, Abmart), rabbit anti-PKA (Cat. No. ab108385, Abcam), rabbit anti-PKA (phospho T197) (Cat. No. ab75991, Abcam), rabbit anti-EP2 (Cat. No. ab167171, Abcam), rabbit anti-NLRP3 (Cat. No. 15101, CST), rabbit anti-Apoptosis-associated speck-like protein (ASC) (Cat. No. 67824, CST), rabbit anti-Caspase-1 (Cat. No. 24232, CST), rabbit anti-NF-κB p65 (Cat. No. 8242S, CST), rabbit anti-NF-κB p50 (Cat. No. 13586, CST), rabbit anti-IL-1β (Cat. No. 31202, CST), mouse anti-β-tubulin (Cat. No. 10094, Proteintech). On the second day, membranes were incubated at RT for 1 h with goat anti-rabbit HRP-conjugated IgG antibody (Cat. No. A0208, Beyotime; 1:10,000), rabbit anti-goat HRP-conjugated IgG antibody (Cat. No. A16136, Invitrogen; 1:5000) or goat anti-mouse HRP-conjugated IgG antibody (Cat. No. A32742, Invitrogen; 1:5000). Subsequntly, membranes were washed three times with TBST. Proteins were visualized using a super-sensitive electrochemiluminescence (ECL) reagent (Cat. No. MA0186, Meilunbio, Dalian, China) with a Molecular Imager ChemiDoc XRS System (Tanon, Shanghai, China). Blots were repeated at least three times, and band density was quantified with the Image J software.

### RNA extraction and qRT-PCR array

Total RNA was extracted from hippocampus or BV2 cells with Trizol (Invitrogen), with purity and concentration measured by NanoDrop 2000 micro-ultraviolet spectrophotometry (1011U, NanoDrop Technologies, Wilmington, DE). RNA was stored at −80 ℃. RNA was reversely transcribed into cDNA using the cDNA Reverse Transcription Kit (Takara, Kusatsu, Japan). qRT-PCR was accomplished using SYBR Premix Ex Taq II (Takara) on an ABI StepOnePlus Real-Time PCR System (Thermo Fisher Scientific). Primers were synthetized by Sangon Bio-tech Co., Ltd. (Shanghai, China) (Table S1). PCR procedure was conducted as follows: 95 °C for 30 s, then 40 cycles of 95 °C for 5 s and 60 °C for 34 s. Target gene expression was normalized to GAPDH, and calculated based on the 2^−ΔΔCt^ method [60].

### Statistical analysis

Data are presented as mean ± SEM and were analyzed with the GraphPad Prism 8.4.3 software. Differences between two groups were analyzed with Student’s *t*-test, and differences between multiple groups were analyzed by one-way ANOVA or two-way ANOVA. *P* < 0.05 was considered as statistically significant.

## Results

### COX-1 is primarily expressed in microglia and COX-2 is mainly expressed in neurons

**Fig. 1:**
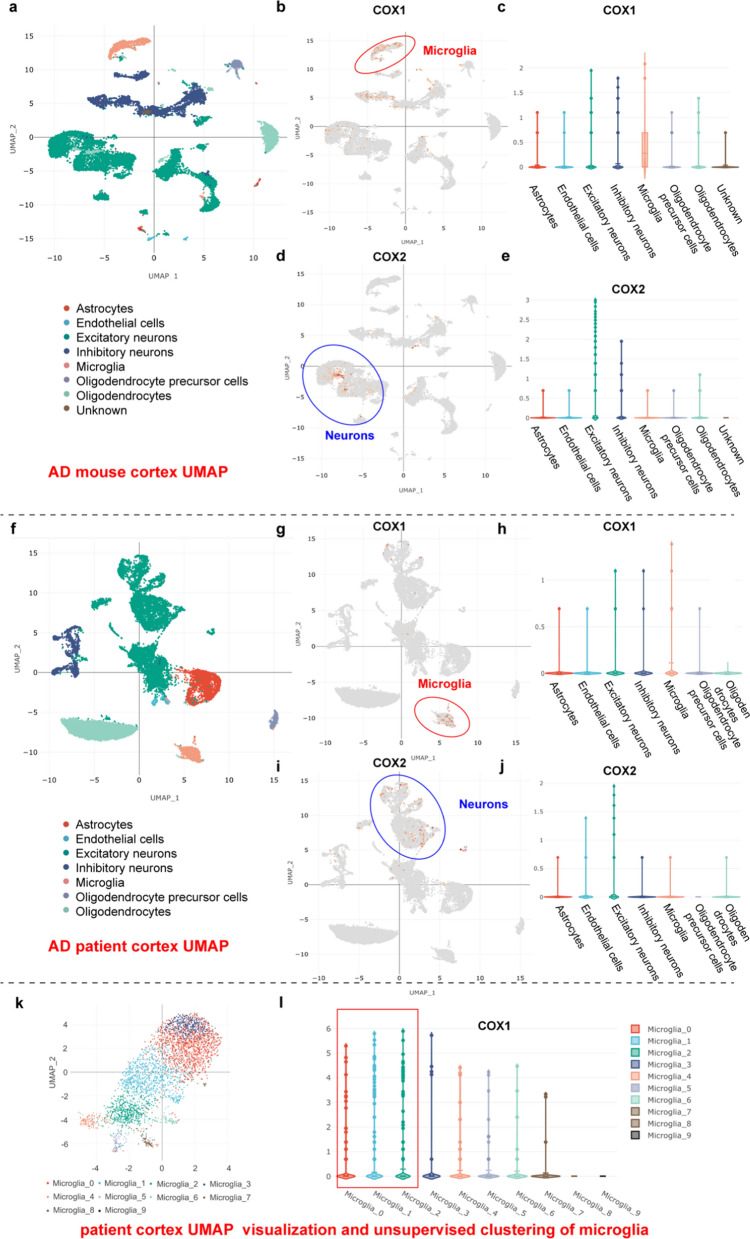
Single-cell expression profiling of COX-1 in different cell types by using ssREAD. **a** UMAP visualization and unsupervised clustering of brain cells derived from AD mice (Data ID: AD00305, male, 7 months old). Colors represent cluster identity. **b** Expression of COX-1 in different subpopulations of brain cells. **c** Average expression of COX-1 across cell clusters. **d** Expression of COX-2 in different subpopulations of brain cells. **e** Average expression of COX-2 across cell clusters. **f** UMAP visualization and unsupervised clustering of brain cells derived from AD patients (Data ID: AD00107, male, 77–90 years). Colors represent cluster identity. **g** Expression of COX-1 in different subpopulations of brain cells. **h** Average expression of COX-1 across cell clusters.** i** Expression of COX-2 in different subpopulations of brain cells. **j** Average expression of COX-2 across cell clusters. **k** Single-cell expression profiling of microglia identified 10 subsets of microglia using ssREAD. UMAP visualization and unsupervised clustering of microglia derived from AD donors (AD01203, males, 72–103 years). Expression of COX-1 in different subpopulations of microglia. **l** Average expression of COX-1 across microglial clusters

To examine the gene expression of COX-1 and COX-2 in the context of aging and AD, we utilized four published datasets in the ssREAD database to compare their expression across various species and cell types [31]. The uniform manifold approximation and projection (UMAP) visualization from the single-cell dataset provides a comprehensive overview of eight distinct cell types, including astrocytes, microglia, neurons, oligodendrocyte progenitor cells, and oligodendrocytes, within the context of AD (Fig. 1a) [33]. Subsequent analysis showed that COX-1 was predominantly expressed in microglia, whereas COX-2 was primarily expressed in excitatory neurons (Fig. 1b–e). Additionally, comparison among AD patients, WT mice, and AD mice consistently showed that COX-1 was more highly detected in microglia, while COX-2 was more prominently expressed in excitatory neurons across all groups (Fig. 1f–j, Fig. S1). Quantitative analysis of COX-1 expression across various cell types revealed that COX-1 is predominantly expressed in microglia (Table S2, from ssREAD database, https://bmblx.bmi.osumc.edu/ssread/, *P* < 0.05). This is consistent with previous research showing that COX-1 is more highly expressed in microglia, where it plays a proinflammatory role in the pathophysiology of AD, in contrast to COX-2 [17, 25].

**Fig. 2:**
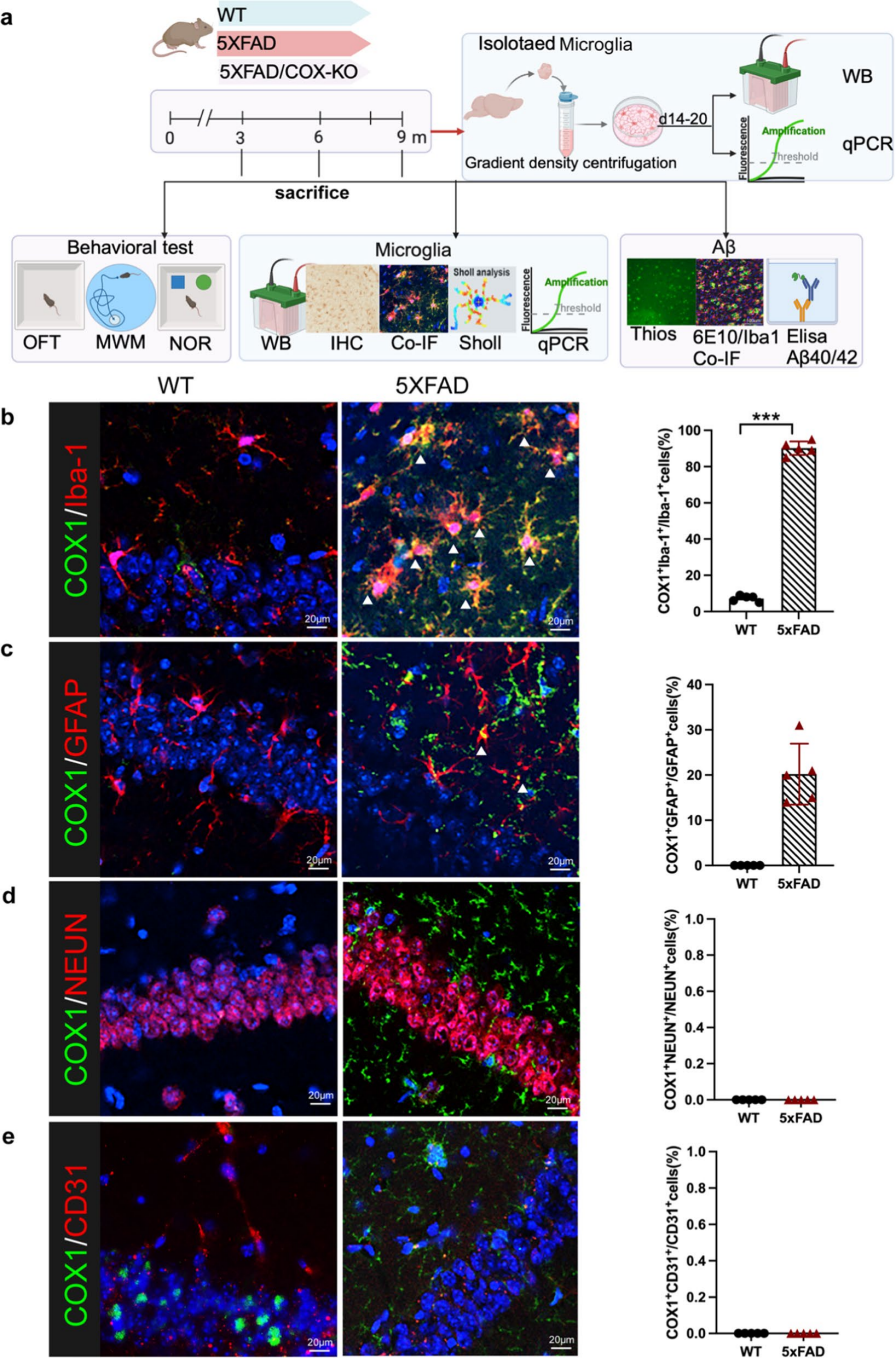
Co-localization of COX-1 with different cell-type-specific markers by immunofluorescence staining. **a** A comprehensive flow diagram outlining the methodological sequence. **b–e** Analysis of co-localization of COX-1 with Iba-1, GFAP, NeuN, and CD31. *n* = 5. ***P < 0.001

We further analyzed colocalization of COX-1 with different cell-type markers (i.e., Iba1-microglia, GFAP-astrocytes, CD31-endothelial cells, NeuN-Neurons). As expected, COX-1 expression was strongly colocalized with Iba1^+^ microglia in the cytoplasm in AD mice (Fig. 2b). Moreover, limited co-localization of COX-1 with GFAP was observed in AD mice (Fig. 2c). COX-1 was not co-localized with CD31^+^ endothelial cells (Fig. 2e) or NeuN^+^ neuron cells (Fig. 2d) in both WT and AD mice. Overall, our results indicated potential role of COX-1, rather than COX-2, in neuroinflammation. Combining single-cell data from mice and humans and IF co-staining, our data establish a central nervous system-specific regulatory role for COX-1 in driving microglia-related neuroinflammatory cascades.

### COX-1 increases in the microglia of 5 × FAD mice in a time-dependent manner

We further conducted a detailed analysis of COX-1 expression across various microglial subpopulations using ssREAD. The UMAP visualization of microglial subtypes revealed 10 distinct subtypes in AD patients (Fig. 1k) [61]. Microglia of cluster 0 exhibit a unique enrichment of genes such as *Axl*, *Clec7a*, and *Cybb*, which are related to a hyperresponsive inflammatory phenotype observed in AD mice. Microglia of cluster 1 specifically express genes related to transcriptional activity. Microglia of cluster 2 exhibit a unique enrichment of genes linked to neurodegenerative diseases, such as *Trem2*, glutamate-ammonia ligase (*Glul*), and S100 calcium-binding protein A (*S100a*), as well as genes associated with activated microglia (Table S3). COX-1 expression was predominantly observed in clusters 0, 1, and 2, which corresponded to elevated expression of pro-inflammatory microglial [34], myeloid neurodegeneration-related [62], and human AD-related single-nucleus [32] gene sets, respectively, when compared to other clusters (Fig. 1l).

**Fig. 3:**
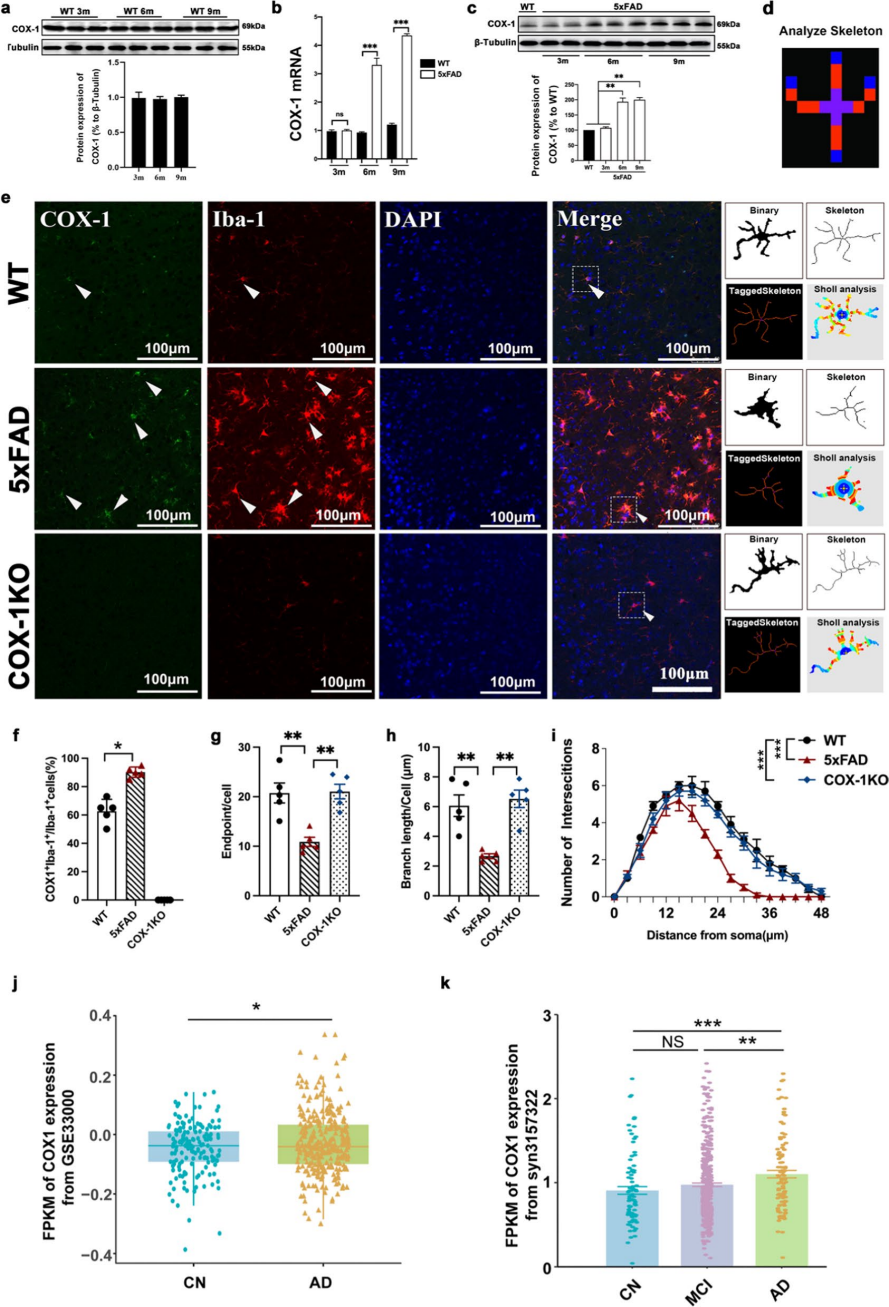
COX-1 expression was increased in an age-dependent manner in AD. **a** COX-1 protein level in the hippocampus of WT mice at 3, 6, and 9 months detected by Western blots (*n* = 3). **b** COX-1 mRNA expression in the hippocampus of WT and 5** × **FAD mice at 3, 6, and 9 months detected by RT-qPCR (*n* = 5). **c** COX-1 protein levels in the hippocampus of WT and 5** × **FAD mice at different ages detected by Western blots (*n* = 3). **d** A representative skeletonized image processed using the Analyze Skeleton plugin of Image J software to identify and tag skeletonized processes as orange, endpoints as blue, and junctions as purple. **e** Double immunofluorescence staining of COX-1 (green) and Iba-1(red) in the cortex of WT, 5** × **FAD and COX-1 KO mice at 9 months old, as well as skeleton analysis and sholl analysis of Iba-1^+^ microglia. **f** The percentage of COX-1^+^Iba-1^+^ to the total Iba^+^ cells (*n* = 5). **g** Quantification of microglial process length/cell in different groups (*n* = 5). **h** Quantificative analysis of microglial endpoints/cell of different groups (*n* = 5). **i** Quantification of the Sholl profiles. Intersections were counted at 0 μm from the soma center to a radius of 48 μm (*n* = 5 mice/group, *n* = 10 cells/mouse). **j** COX1 expression from GEO database (GSE33000) in CN and AD patients. **k** COX1 expression from the GEO database (syn3157322) among CN, MCI, and AD. Means ± SEM; ns: nonstatistical significance; ^*^P < 0.05, ^**^P < 0.01, ^***^P < 0.001

Next, we assessed COX-1 expression in 3-, 6- and 9-month-old WT mice. The protein level and mRNA expression of COX-1 in the WT group were relatively consistent across age groups (Fig. 3a, b and Fig. S2a). However, COX-1 mRNA and protein were significantly increased from 3 to 9 months in the hippocampus of 5 × FAD mice (Fig. 3a–c, and Fig. S2a). No significant differences in COX-2 expression were detected among WT and 5 × FAD mice across the 3-, 6-, and 9-month age groups (Fig. S2b) or in isolated microglia (Fig. S2c). COX-1 was prominently colocalized with Iba1^+^ microglia and increased in 9-month-old 5 × FAD mice compared to WT mice (Fig. 3e, f ). Quantitative skeleton analysis and sholl analysis showed that microglia exhibited a highly ramified morphology characterized by extensive arborization in WT and COX-1 KO mice. In contrast, microglia in 5 × FAD mice displayed progressive morphological transformation to an amoeboid phenotype with significant process retraction (Fig. 3g–i).

To further investigate the expression patterns of COX-1 and COX-2 in AD, we analyzed transcriptomic datasets from the GEO database. In the GSE33000 cohort, a significant upregulation of COX-1 expression in AD patients was observed when compared with the CN group (Fig. 3j), whereas no significant changes of COX-2 expression were observed (Fig. S2d). Moreover, analysis of the syn3157322 dataset revealed increased expression of COX1 in MCI and AD patients compared with the CN group in a stage-dependent manner (Fig. 3k). These results align with our findings from AD model, where hippocampal COX-1 expression increased at 6 and 9 months, with no significant alterations of COX-2.

### COX-1 deletion leads to changes of microglial phenotypes and alleviates neuroinflammatory responses in 5 × FAD mice

**Fig. 4:**
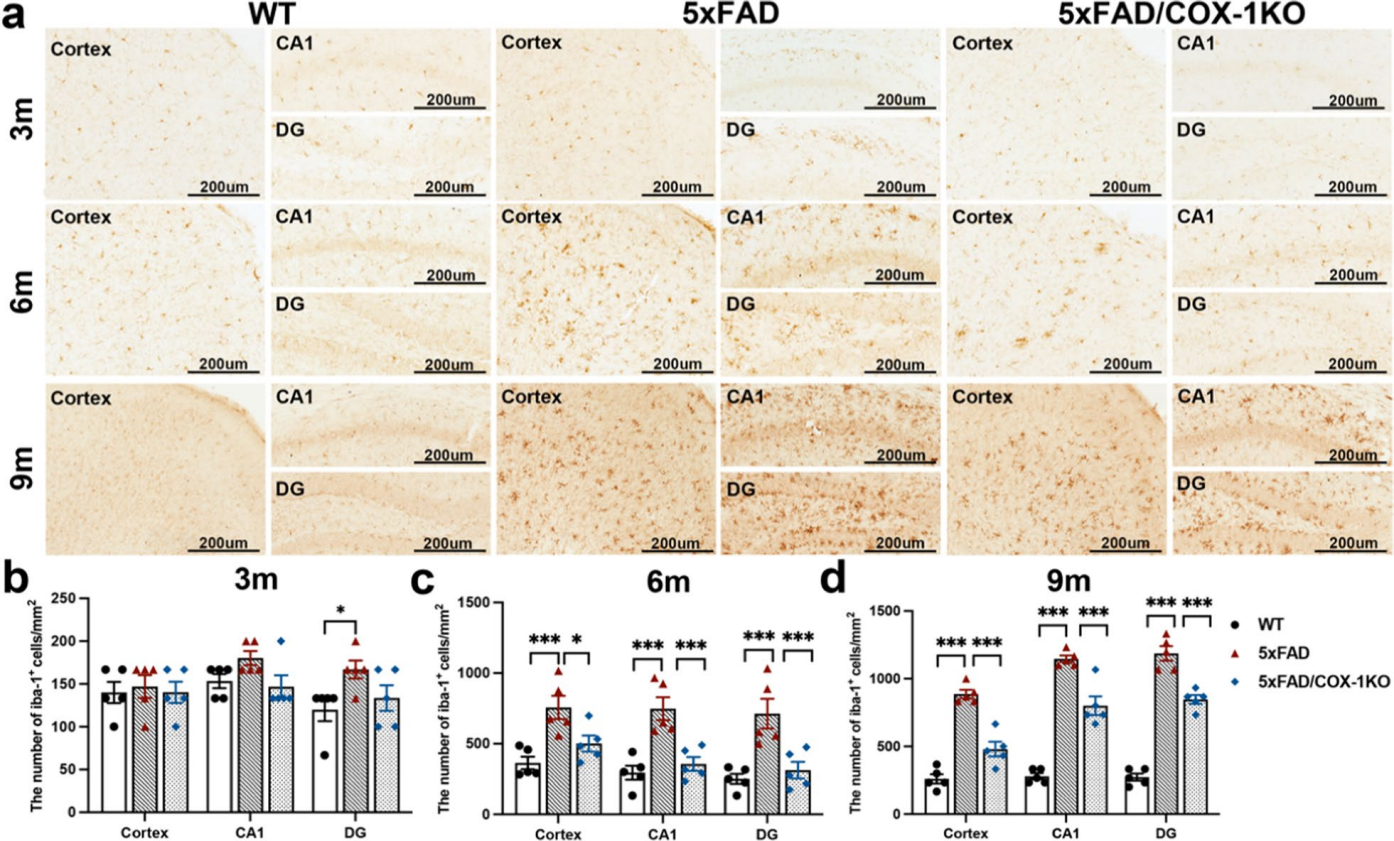
COX-1 deletion reduces the number of Iba1^+^ microglia in 5 × FAD mice. **a** The numbers of Iba-1^+^ cells was evaluated by immunohistochemistry with Iba-1 antibody in the cortex, CA1 and DG regions of WT, 5 × FAD and 5 × FAD/COX-1 KO mice at different ages (3-, 6-, and 9-month-old) (*n* = 5). **b**-**d** Quantification of Iba-1^+^ cells in the cortex, CA1 and DG regions of WT, 5 × FAD and 5 × FAD/COX-1 KO mice at different ages (3, 6, and 9 months) (*n* = 5). Means ± SEM; ^*^P < 0.05, ^***^P < 0.001

To investigate the role of COX-1 in microglia-induced neuroinflammation, we generated 5 × FAD/COX-1 KO mice (Fig. S2e, f). The age-dependent increase in Iba-1^+^ microglia in the cortex, CA1, and dentate gyrus (DG) in the 5 × FAD group compared to the WT group, was reversed in the 5 × FAD/COX-1 KO mice (Fig. 4).

**Fig. 5:**
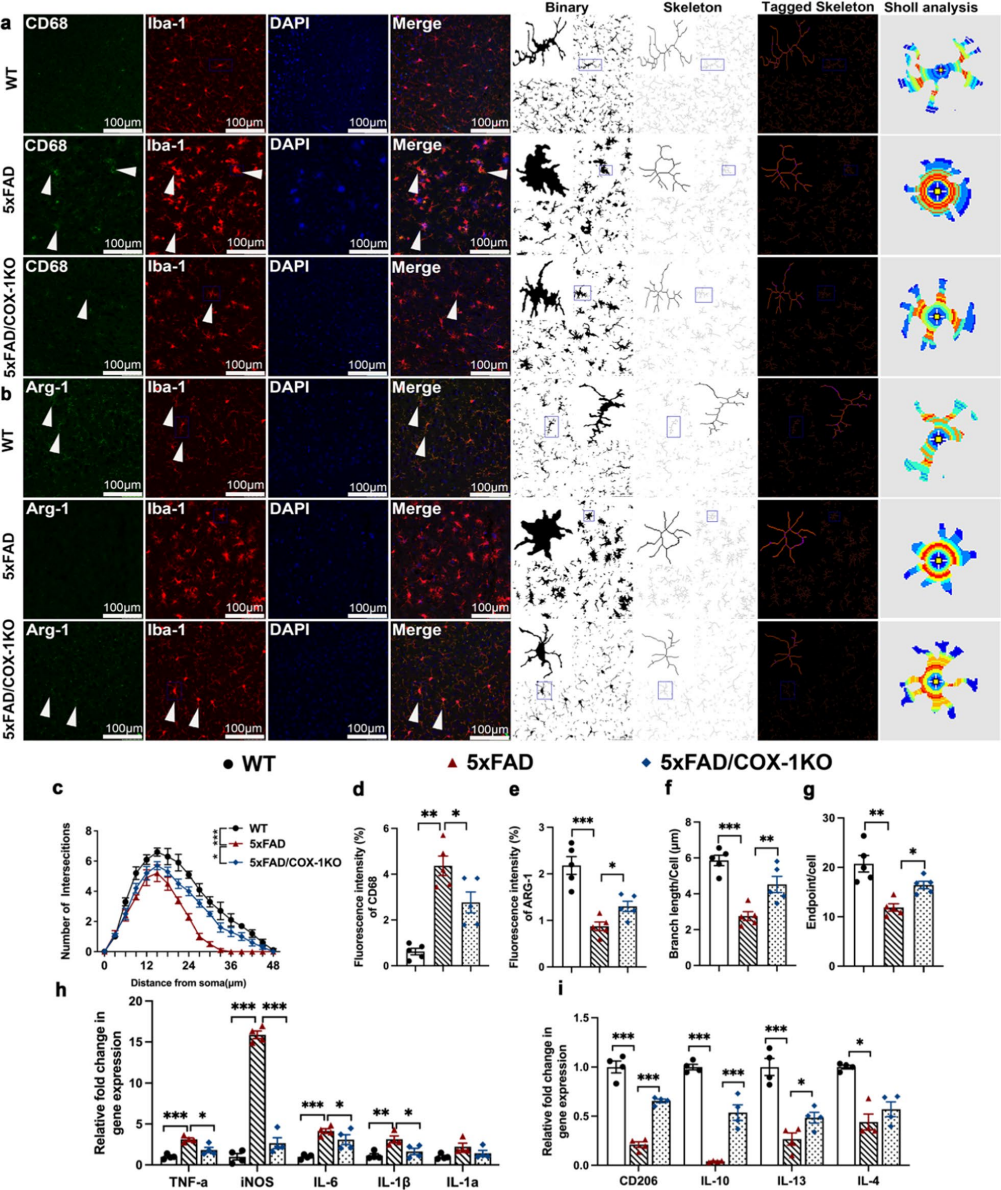
COX-1 deletion reverses the morphological and phenotypic changes of microglia in 5 × FAD mice. **a, b** Double immunofluorescence staining for Iba-1(red) and CD68 (green) or Arg-1 (green) in the cortex of mice at age of 9 months, as well as skeleton analysis and sholl analysis of Iba-1^+^ microglia (*n* = 5). **c** Quantification of the Sholl profiles for the three groups. Intersections were counted at 0 μm from the soma center to a radius of 48 μm (*n* = 5 mice/group, *n* = 10 cells/mouse). **d, e** Fluorescence intensity of CD68 and Arg-1 in different groups (*n* = 5). **f, g** Microglial process length/cell and microglial endpoints/cell in different groups (*n* = 5). **h** mRNA expression of proinflammatory cytokines (TNF-α, iNOS, IL-6, IL-1β and IL-1α) in different groups analysed by qRT-PCR (*n* = 5). **i** mRNA expression of anti-inflammatory cytokines (CD206, IL-10, IL-13 and IL-4) in different groups analysed by qRT-PCR (*n* = 5). Means ± SEM; ^*^P < 0.05, ^**^P < 0.01, ^***^P < 0.001; Scale bar = 100 μm

Microglia with ramified morphology and Arg1 expression are associated with Aβ phagocytosis, whereas amoeboid microglia expressing CD68 secrete proinflammatory cytokines IL-1β and TNF-α. These phenotypes align with the transcriptional signatures of DAM-like and inflammatory microglial subsets reported in AD models [13, 14]. To further determine the impact of COX-1 deletion on the transformation of microglial morphology and phenotypes in AD mice, we sequentially labeled microglia by CD68, a surface maker of activated microglia, and Arg-1, which is mainly expressed in resting microglia. Moreover, the total length of branches and the number of branching endpoints of each microglia were also calculated by two-dimensional reconstruction of microglia [63]. With AD progression, microglia became round and CD68^+^ microglia were significantly increased, with low levels of Arg1. In contrast, 5 × FAD/COX-1 KO mice showed reduced Iba1^+^CD68^+^ microglia and increased Iba1^+^Arg1^+^ microglia (Fig. 5a, b, d, e). Quantitative skeleton analysis and sholl analysis revealed significantly reduced branching complexity of microglial processes in 5 × FAD mice, indicative of activated amoeboid morphology. Strikingly, COX-1 deletion restored ramified complexity, with increased process length and terminal endpoints (Fig. 5a, c, f, g). After COX-1 deletion, microglia remained somewhat hypertrophic, suggesting that microglial activation may be influenced by other regulatory mechanisms and signaling pathways involved in neuroinflammation.

Furthermore, we found downregulation of proinflammatory factors *Tnf-α*, *iNOS*,* Il-6*, and* Il-1β* (Fig. 5h) and upregulation of anti-inflammatory cytokines *CD206*,* Il-10*,* Il-13*, and* Il-4* (Fig. 5i) in 5 × FAD/COX-1 KO mice, compared with 5 × FAD mice. Overall, COX-1 KO consistently changed the phenotypes of microglia and alleviated neuroinflammation in 5 × FAD mice. However, COX1-KO did not affect morphology of astrocytes or neuronal populations in 5 × FAD mice (Fig. S2h), highlighting its specific role in inflammation over cellular morphogenesis. These findings establish COX-1 as a critical regulator of microglial polarization and neuroinflammatory progression in AD pathogenesis.

Moreover, we evaluated the safety of COX1 KO in 5 × FAD mice. Evans Blue staining demonstrated no evident cerebral hemorrhage (e.g., dye leakage or vascular rupture) in each group. However, 9-month-old 5 × FAD and 5 × FAD/COX-1 KO mice revealed higher brain dye content than WT mice, indicating increased BBB permeability. This may be attributed to the inherent BBB disruption caused by AD progression, according to the absence of significant differences between the 5 × FAD/COX1 KO and 5 × FAD groups (Fig. S3a). Moreover, HE staining revealed no characteristic signs of cerebral hemorrhage (e.g., erythrocyte extravasation or vascular disruption) in the hippocampal and cortical regions of 9-month-old WT, 5 × FAD, and 5 × FAD/COX-1 KO mice (Fig. S3b). The tail bleeding assay was also performed to evaluate bleeding risk under specific conditions. The results showed that COX-1 KO induced a modest prolongation of bleeding time in the AD mice (Fig. S3c). Multi-organ HE analysis across all experimental groups revealed preserved organ architecture without evidence of pathological alterations and bleeding events (Fig. S3d). Finally, liver function (ALT, AST) and renal function (urea, creatinine) were comparable among these groups (Fig. S3e–h). These results confirmed that COX-1 inhibition does not induce spontaneous microhemorrhage or clinically relevant bleeding events or systemic toxicity, but induces modest prolongation of bleeding time under specific conditions.

### COX-1 deletion reduces the levels of Aβ in the brains of 5 × FAD mice

**Fig. 6:**
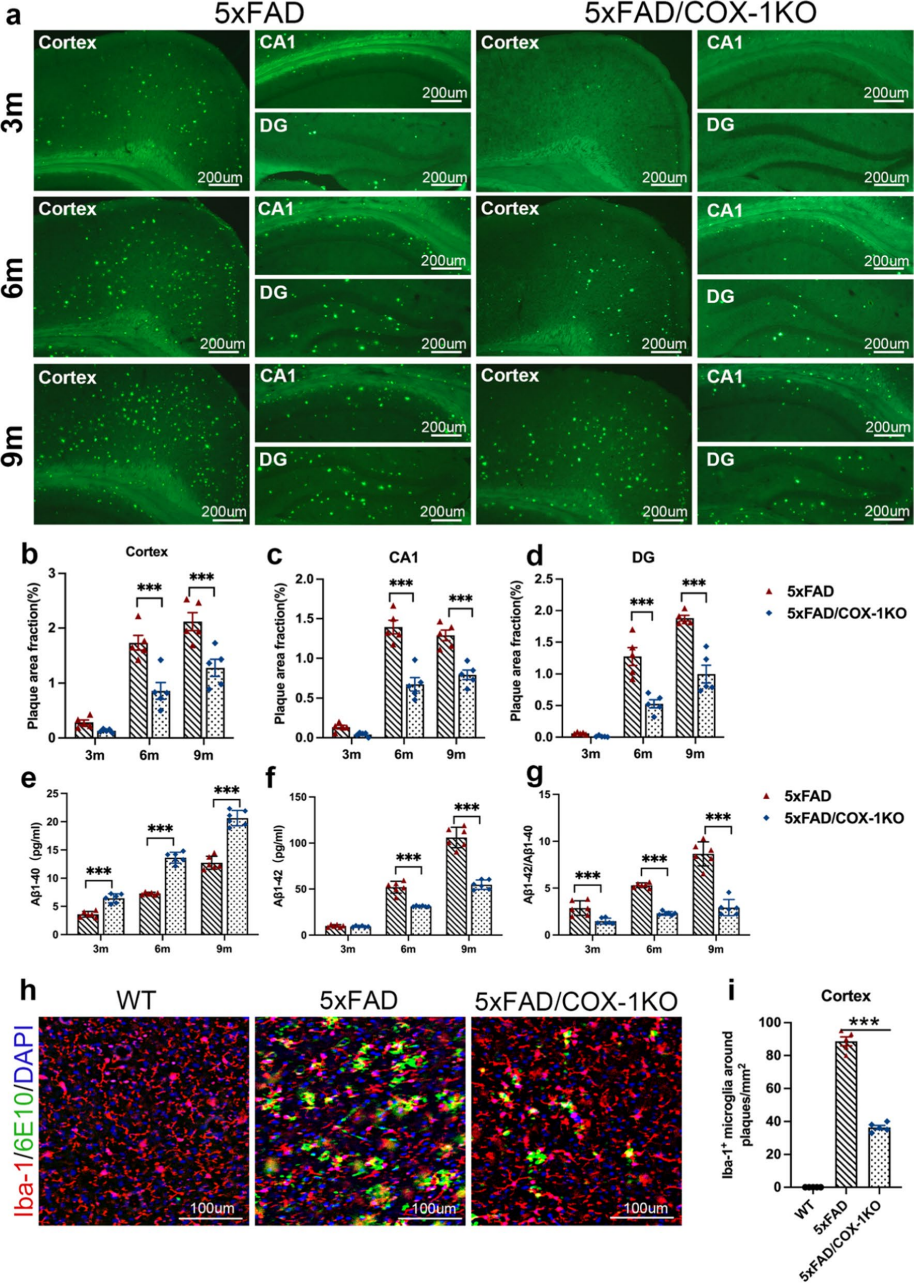
COX-1 deletion reduces the deposition of Aβ in 5 × FAD mice. **a** Amyloid plaques labeled with thioflavin S in the cortex, CA1 and DG of 5 × FAD and 5 × FAD/COX-1 KO mice at different ages (3, 6, and 9 months, *n* = 5). **b**-**d** Quantification of amyloid plaques in the cortex, CA1 and DG regions of 5 × FAD and 5 × FAD/COX-1 KO mice at different ages (3, 6, and 9 months, *n* = 5). **e**–**g** Levels of Aβ1-40 and Aβ1-42 as well as the Aβ1-42/Aβ1-40 ratio (*n* = 6). **h** Iba-1 (red) and 6E10 (green) immunostaining in the cortex of WT, 5 × FAD and 5 × FAD/COX-1 KO mice. **i** Iba-1^+^ microglia around plaques/mm^2^ (*n* = 5). Means ± SEM; ^*^P < 0.05, ^**^P < 0.01, ^***^P < 0.001

The pathological hallmark of AD is characterized by the aggregation and deposition of Aβ in the brain. Our study demonstrated that the plaque area fraction (%) in the cortex and hippocampal CA1 and DG regions, exhibited an age-dependent increase in 5 × FAD mice (Fig. 6a–d). Conversely, significant reductions of Aβ deposition were observed in these regions in 5 × FAD/COX-1 KO mice, compared to age-matched 5 × FAD mice (Fig. 6a–d). Moreover, the level of Aβ1-40 increased in 5 × FAD/COX-1 KO mouse cortex, compared to 5 × FAD mice (Fig. 6e). In addition, the level of Aβ1-42 and the Aβ1-42/Aβ1-40 ratio decreased in the cortex of 5 × FAD/COX-1 KO mice, compared to 5 × FAD mice (Fig. 6f, g). Finally, we found there were significantly increased levels of 6E10^+^ plaque-around Iba1^+^ microglia in the cortex of 5 × FAD mice, compared to WT (Fig. 6h, i). COX-1 KO significantly reduced the plaque-associated Iba1^+^ microglia (Fig. 6h–i). Our results support the predominant role of Aβ42 in AD pathogenesis and suggest that COX-1 deletion may preferentially target Aβ42 catabolism through mechanisms potentially involving microglial phenotype transition.

### COX-1 deletion improves the motor ability and cognitive function of 5 × FAD mice

**Fig. 7:**
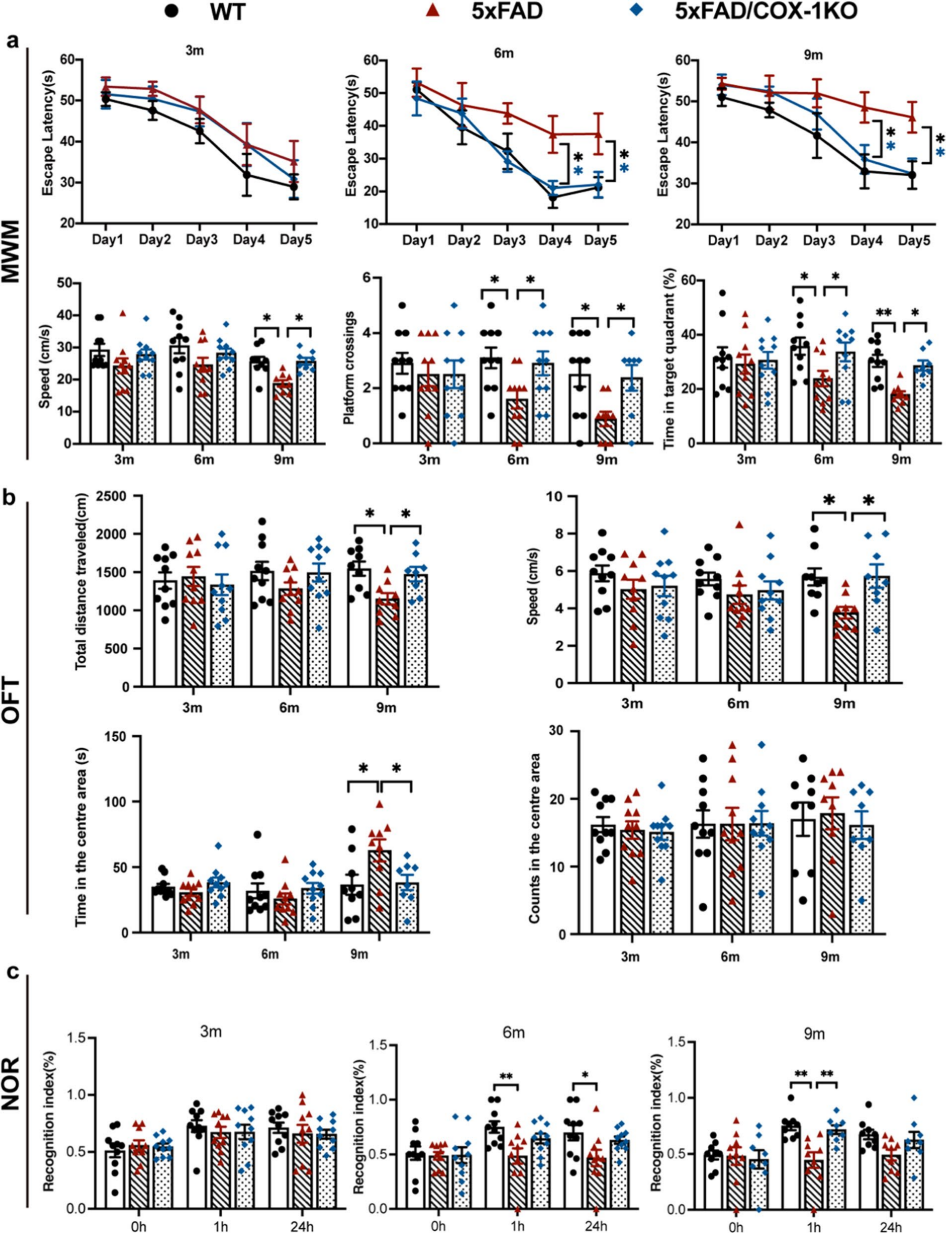
COX-1 deletion improves the motor ability and cognitive function of 5 × FAD mice. **a** Escape latency during the 5 training days, as well as the swimming speed, number of platform crossings and time spent in target quadrant during the probe trial (day 6) in the Morris water maze (*n* = 8–10). **b** Anxiety-like behavior and the motor ability of WT and 5 × FAD mice at different ages (3, 6, and 9 months) in the open field test (*n* = 8–10). **c** Memory performance of WT and 5 × FAD mice at different ages (3, 6, and 9 months) in the novel object recognition test (*n* = 8–10). Means ± SEM; ^*^P < 0.05, ^**^P < 0.01

In the MWM, the 6- and 9-month-old 5 × FAD mice showed significantly longer escape latency to find the hidden platform on days 4 and 5, and decreased number of crossings on day 6. Moreover, the 6- and 9-month-old 5 × FAD/COX-1 KO mice showed shorter latency to find the hidden platform on days 4 and 5, and increased number of crossings on day 6 compared to the 5 × FAD mice (Fig. 7a). Moreover, the swimming speed decreased significantly in 9-month-old 5 × FAD mice but was restored in age-matched 5 × FAD/COX-1 KO mice.

In the OFT, 5 × FAD mice exhibited locomotor deficits, manifested as initial decline of total movement distance and speed at 3 and 6 months without statistical significance, reaching significance at 9 months—a pathological trajectory substantially ameliorated by COX1-KO. In addition, the 9-month-old 5 × FAD mice spent significantly more time in the central region than WT mice, but the time was significantly reduced by COX-1 KO. However, there was no difference in the number of crossings in the central region among the groups (Fig. 7b). These results suggest that the speed of movement decreased in 9-month-old 5 × FAD mice compared with WT mice, but improved in 5 × FAD/COX-1 KO mice. Finally, cognitive impairment emerged with significant working memory deficits detectable by 6 months in the NORT. While COX1-KO provided modest protection at this stage, significant rescue of memory ability emerged only at 9 months in 1-h testing, coinciding with advanced pathological progression of AD (Fig. 7c). Hence, the above results suggest that COX-1 deficiency might attenuate the impairments in motor ability and cognitive function in 5 × FAD mice.

### The inflammatory PGE_2_/EP2 receptor is the downstream effector of COX-1-mediated neuroinflammation in 5 × FAD mice

**Fig. 8:**
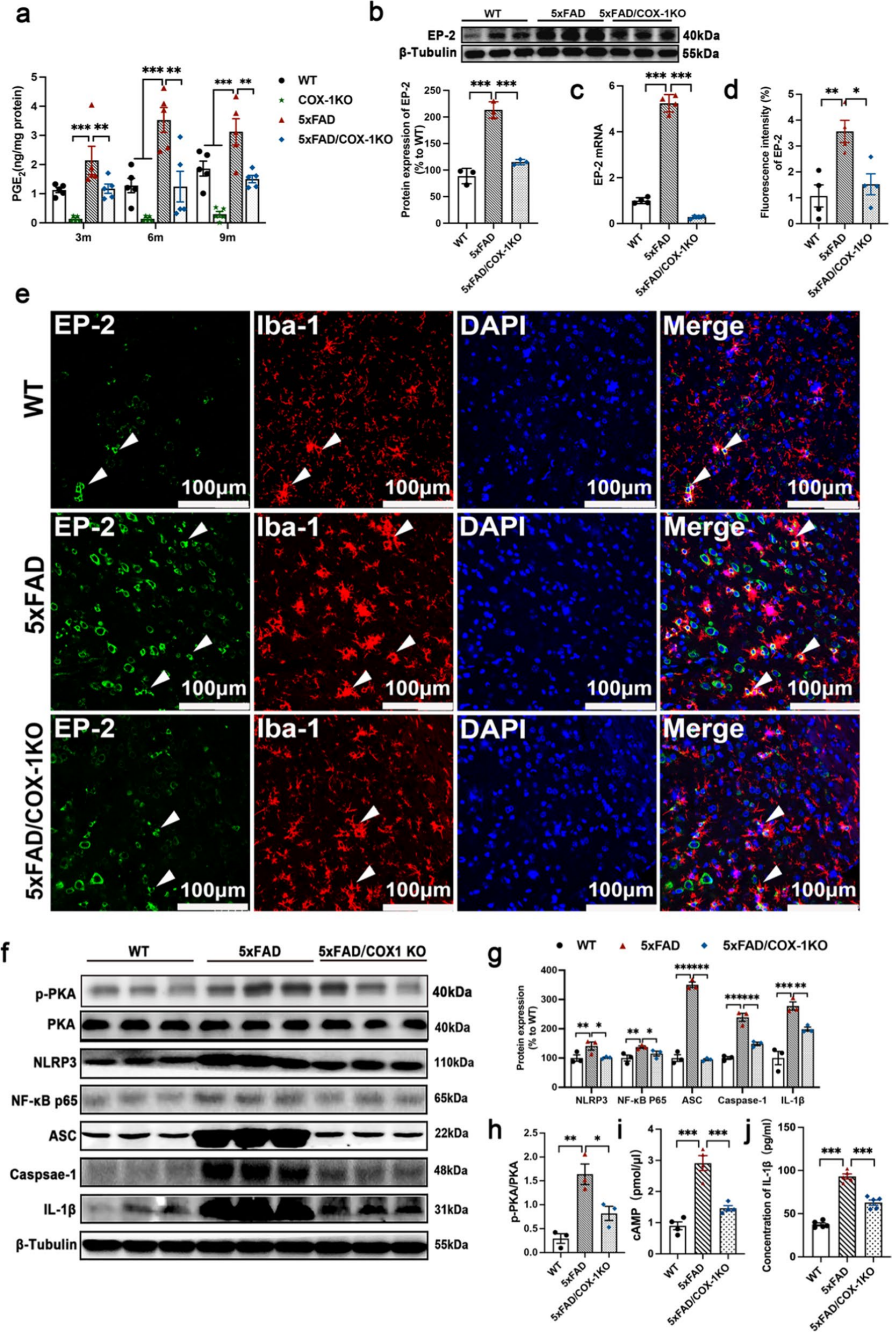
Deficiency of COX-1 inhibits NLRP3 inflammasomes through the PGE2/EP2 pathway in 5 × FAD mice. **a** PGE_2_ levels in the hippocampus detected by HPLC/mass spectrometry (*n* = 5). **b** EP2 protein levels in the hippocampus at age of 9 months detected by Western blotting (*n* = 3). **c** mRNA expression of EP2 in the hippocampus at age of 9 months determined by qRT-PCR (*n* = 4). **d, e** Double immunofluorescence staining for EP2 (green) and Iba-1(red) in the cortex and quantification of EP-2 fluorescence intensity (*n* = 4). **f**–**h** Proteins levels of p-PKA, PKA, NF-κB p65, NLRP3, ASC, Caspase-1 and IL-1β in the hippocampus detected by Western blotting (*n* = 3). **i**, **j** cAMP and IL-1β levels in the hippocampus of different groups tested by ELISA. Means ± SEM; ^*^P < 0.05, ^**^P < 0.01, ^***^P < 0.001

COX-1 catalyzes the rate-limiting step in the formation of prostaglandin H2 (PGH_2_) from arachidonic acid stored in the membrane. PGH_2_ is further isomerized to each terminal PG, including PGE_2_, PGD_2_, PGI_2_, PGF_2a_, and thromboxane A2 (TxA_2_)_,_ by the corresponding synthases [64]. Our HPLC/MS results revealed higher levels of PGD_2_, PGE_2_, and TxB_2_ (metabolite of TxA_2_) in 5 × FAD mice than in WT mice at both 6 and 9 months of age (Fig. 8a, Fig. S4a). However, the levels of PGF_2a_ and 6-Keto-PGF_1a_ (principal metabolite of PGI_2_) were not significantly different between 9-month-old WT and 5 × FAD mice (Fig. S4a). Importantly, COX-1 deletion decreased all types of PGs in 5 × FAD mice at different ages (Fig. 8a and Fig. S4a).

**Fig. 9:**
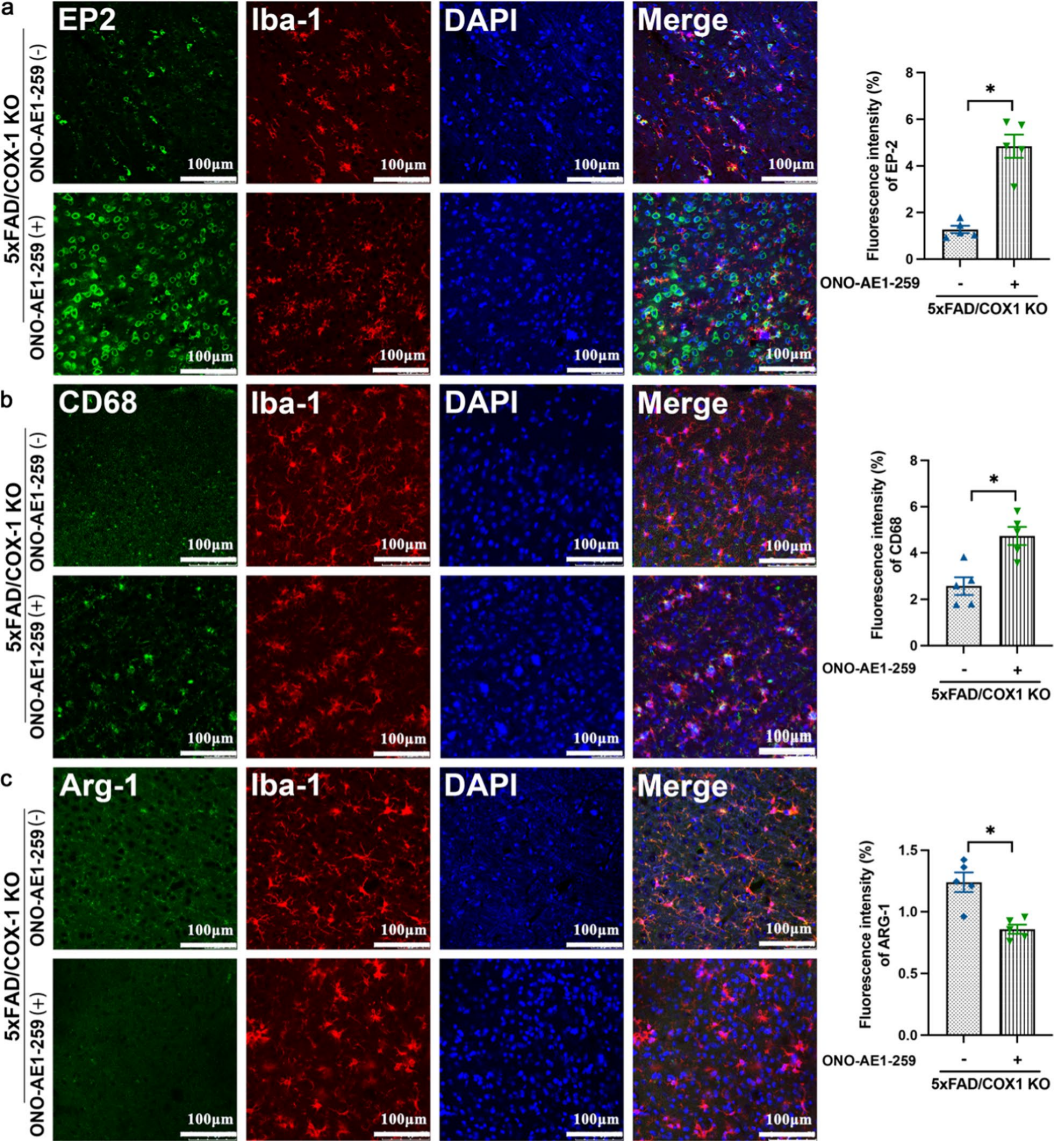
EP2 alters the microglial polarization state induced by COX-1 deletion in 5 × FAD mice. **a-c** Double immunofluorescence staining for EP2 (**a**), CD68 (**b**), or Arg-1 (**c**) with Iba-1 in the cortex of 5 × FAD/COX-1 KO mice with and without ONO-AE1-259 (an agonist of EP2) treatment, and quantification of fluorescence intensity (*n* = 5). Means ± SEM; ^*^P < 0.05

PGD_2_ and PGE_2_ play crucial roles in AD pathology [65, 66]. PGD_2_ exerts its cellular effects through activation of two distinct D-prostanoid G protein-coupled receptors, DP1 and DP2. Similarly, PGE_2_ exerts its multiple functions by binding to high-affinity G-protein coupled receptors, including EP1, EP2, EP3, and EP4 [47]. We next sought to identify whether PGE_2_ signaling through EP2 receptor is involved in the inflammatory responses in 5 × FAD mice. The mRNA expression of EP2 and EP4 was significantly higher in 5 × FAD mice than in WT mice (Fig. 8c and Fig. S4b). The EP4 receptor appears to be protective in AD [67], whereas the EP2 receptor seems to have detrimental effects [68, 69]. Our results showed that both the protein and the mRNA levels of EP2 were significantly higher in 5 × FAD mice than in WT mice, whereas COX-1 deletion inhibited the EP2 upregulation (Fig. 8b, c). EP2 was localized to Iba-1^+^ microglia in the cortex (Fig. 8e). EP2 fluorescence intensity was much higher in 5 × FAD mice compared to the WT mice, whereas COX-1 KO reversed this increase (Fig. 8d). Moreover, to further clarify the function of EP2 in the regulation of microglial state, we treated 5 × FAD/COX-1 KO mice with the EP2 agonist ONO-AE1-259. We found that ONO-AE1-259 increased the expression of EP2 in Iba-1^+^ microglia and reversed the change of microglial state in 5 × FAD/COX-1 KO mice (Fig. 9a–c).

Taken together, pharmacological activation of EP2 signaling altered the microglial polarization states, supporting the critical involvement of the key prostaglandin receptor EP2 in neuroinflammation in 5 × FAD mice. Our findings indicate that PGE_2_/EP2 signaling is essential for the neuroinflammation mediated by COX-1-derived PGE_2_.

### COX-1 deletion inhibits NLRP3 inflammasomes via the PGE2/EP2/cAMP/PKA/NF-κB pathway in 5 × FAD mice

The EP2 receptor can mediate the activity of the intracellular cAMP/PKA pathway induced by PGE_2_ [70]. Moreover, the PGE_2_/EP2 pathway may activate the cAMP–PKA–NF-κB axis and trigger activation of NLRP3 inflammasomes in scorpion envenomation [27]. NLRP3 plays a key role in AD pathogenesis, including regulating the deposition of Aβ, altering the morphology of microglia, and impairing synaptic plasticity and cognitive function [71, 72]. To determine whether EP2 can activate NLRP3 inflammasomes via the cAMP–PKA–NF-κB axis in 5 × FAD mice, we first examined the levels of cAMP in 5 × FAD mice. The level of cAMP in 5 × FAD mice was significantly higher than that in WT mice, and COX-1 KO reversed this increase (Fig. 8i). Western blotting showed that the PKA/NF-κB p65 pathway was activated, accompanied by significantly increased protein levels of NLRP3 inflammasome-related proteins NLRP3, ASC, Caspase-1 and IL-1β in 5 × FAD mice compared with WT mice, and COX-1 KO blocked this activation (Fig. 8f–h, j). These results suggest that EP2 may activate NLRP3 inflammasomes via the cAMP–PKA–NF-κB p65 pathway in 5 × FAD mice.

### COX-1 activates the cAMP–PKA–NF-κB p65 axis and NLRP3 inflammasomes through the PGE_2_/EP2 pathway in vitro

**Fig. 10:**
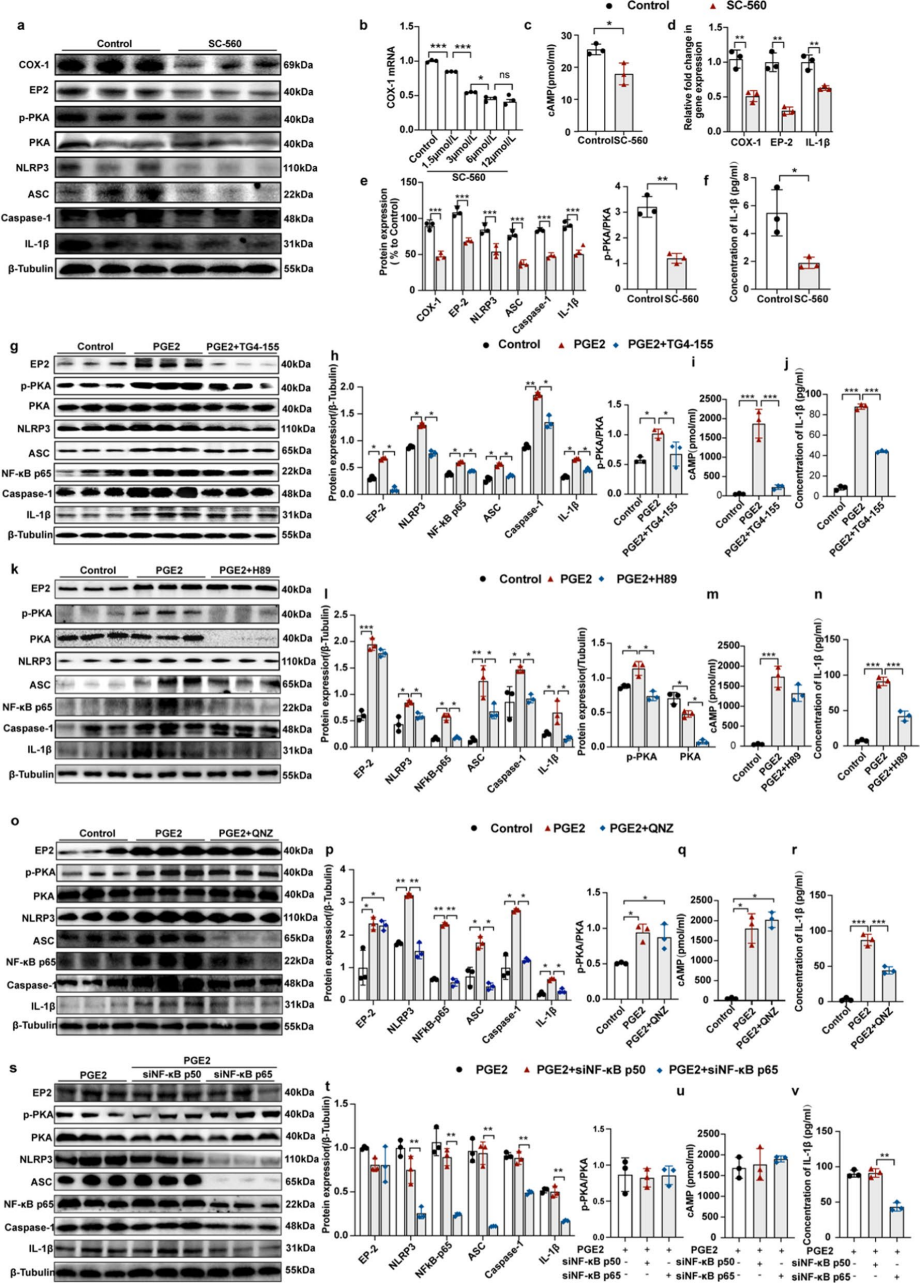
Suppression of COX-1 inhibits NLRP3 inflammasomes by inhibiting the EP2/cAMP/PKA/NF-κB p65 pathway in BV2 cells. **a, e** Western blotting for COX-1, p-PKA, PKA, EP2, NLRP3, ASC, Caspase-1 and IL-1β in BV2 cells with or without SC-560 (an inhibitor of COX-1) treatment (*n* = 3). **b** qRT-PCR analysis of COX-1 mRNA expression in BV2 cells with SC-560 treatment at different concentrations (*n* = 3). **c, f** cAMP and IL-1β levels detected by ELISA (*n* = 3). **d** qRT-PCR analysis of COX-1, EP2 and IL-1β mRNA expression in BV2 cells with or without SC-560 treatment (*n* = 3). **g, h** Western blotting for EP2, NLRP3, p-PKA, PKA,NF-κB p65, ASC, Caspase-1 and IL-1β in BV2 cells treated with PGE_2_ or PGE_2_ plus EP2 inhibitor TG4-155 (*n* = 3). **i, j** cAMP and IL-1β levels detected by ELISA (*n* = 3). **k**, **l** Protein levels of EP2, NLRP3, PKA, p-PKA, NF-κB p65, ASC, Caspase-1 and IL-1β in BV2 cells with PGE2 or PGE2 plus PKA inhibitor H89 treatment (*n* = 3). **m**, **n** cAMP and IL-1β levels detected by ELISA (*n* = 3). **o**, **p** Protein levels of EP2, NLRP3, NF-κB p65, ASC, Caspase-1 and IL-1β in BV2 cells with PGE_2_ or PGE_2_ plus NF-κB inhibitor QNZ treatment (*n* = 3). **q, r** cAMP and IL-1β level detected by ELISA (*n* = 3). **s**, **t** Protein levels of EP2, NLRP3, NF-κB p65, ASC, Caspase-1 and IL-1β in BV2 cells treated with PGE_2_ plus siRNAs NC, or siRNA for NF-κB p65 or NF-κB p50 (*n* = 3). **u**, **v** cAMP and IL-1β levels detected by ELISA (*n* = 3). Means ± SEM; ^*^P < 0.05, ^**^P < 0.01, ^***^P < 0.001

We further investigated the role of COX-1 in the NLRP3 inflammasome activation and related intracellular signaling pathways in vitro using a cultured microglial line, BV2 cells. SC-560 (a selective inhibitor of COX-1) inhibited COX-1 mRNA expression in a dose-dependent manner (Fig. 10b), so the concentration of 6 μmol/L was selected for the following experiments. SC-560 significantly downregulated the level of cAMP (Fig. 10c) and inhibited the EP2/cAMP/PKA/NF-κB pathway and protein levels of NLRP3 inflammasome-related proteins (NLRP3, ASC, Caspase-1 and IL-1β) in BV2 cells (Fig. 10e, f). To investigate the role of PGE_2_, we used PGE_2_ to activate BV2 cells. PGE_2_ indeed activated the EP2/cAMP/PKA/NF-κB pathway and the NLRP3 inflammasome (Fig. 10g–j). PGE_2_ induced a significant increase in p-NF-κB in BV2 cells (Fig. S4g). TG4-155 (an inhibitor of EP2), H89 (an inhibitor of PKA) and QNZ (an inhibitor of NF-κB) inhibited the effect of PGE_2_ on the NLRP3 inflammasome (Fig. 10g, h, j, k, l, n, o, p, r and Fig. S4c–f). In addition, TG4-155 decreased the level of cAMP, while H89 and QNZ did not (Fig. 10i, m and q). Since NF-κB functions as a homo- and hetero-dimer, we further investigated the role of NF-κB p65 and p50 using p65- and p50-specific siRNAs. Results showed that the NF-κB p65 siRNA inhibited the expression of NLRP3 inflammasome-related proteins (NLRP3, ASC, Caspase-1 and IL-1β) (Fig. 10s, t and v). Together, these results suggest that COX-1 may activate the cAMP‒PKA‒NFκB p65 axis and NLRP3 inflammasomes through the PGE_2_‒EP2 pathway in vitro.

## Discussion

Over the years, major phase 2–3 clinical trials for AD treatment have not achieved desirable effects [73, 74]. Neuroinflammation is recognized as a hallmark feature that contributes to neuronal impairment in AD [75]. Microglial overactivation plays a pivotal role in neuroinflammation and, in the context of AD, contributes to synaptic dysfunction, synaptic loss, and neuronal impairment [76, 77]. COX-1, a target irreversibly inactivated by aspirin, is highly expressed in microglia associated with neuroinflammation [17]. In this study, we present evidence highlighting the COX-1–microglial activation axis as an effective target for AD treatment. By using 5 × FAD mice, we found that COX-1 is primarily expressed in microglia, and its level increases in an age-dependent manner in 5 × FAD mice. Furthermore, COX-1 KO in AD mice restored cognitive impairment, accompanied by reduced accumulation of Aβ protein in the cerebral cortex and hippocampus. Furthermore, our study revealed an age-associated increase of microglia in 5 × FAD mice, which correlated with Aβ deposition. Notably, COX-1 deficiency attenuated the hyperinflammatory microglial state (evidenced by reduced CD68 and IL-1β) and promoted a phagocytic, homeostatic phenotype (elevated Arg1 and CD206), mirroring the DAM-like transcriptional profile observed in early AD [14].

Under specific conditions like accumulation of reactive oxygen species, microglia secrete pro-inflammatory cytokines in proximity to Aβ plaques [78, 79]. Increased levels of serum TNF-α and IL-1β correlate with an accelerated rate of cognitive impairment in AD patients [80]. In our study, we observed downregulation of proinflammatory mediators (*Tnf-α, iNOS, IL-6,* and *IL-1*) and upregulation of anti-inflammatory cytokines (*Cd206, IL-10, IL-13,* and *IL-4*) in 5 × FAD/COX-1 KO mice. A previous study showed that COX-1 KO mice exhibit reduced neuroinflammation induced by Aβ and attenuated plaque-associated microgliosis [19]. Moreover, NLRP3 inflammasome activation exacerbates Aβ aggregation and impairs microglial phagocytosis [59]. In the 5 × FAD/COX-1 KO mice, microglia shifted toward an anti-inflammatory phenotype, associated with enhanced Aβ phagocytosis. This suggests that COX-1 deletion may alleviate the PGE/EP2-driven lysosomal impairment, enabling anti-inflammatory microglia to clear Aβ more efficiently. Moreover, we observed a significant reduction in Aβ42 in 9-month-old 5 × FAD/COX-1 KO mice compared to 5 × FAD mice. These results indicate that COX-1 deletion reduces Aβ plaques by reprogramming microglia toward a pro-phagocytic, anti-inflammatory state. However, a study by Park et al. [81] in APP/PS1 mice showed no Aβ reduction with COX-1 deletion, whereas Choi et al. [26] demonstrated reduced pathology in 3 × Tg-AD mice. Our work resolves this discrepancy through two critical insights from the 5 × FAD/COX-1-KO model. Firstly, the 5 × FAD mice uniquely exhibit early-onset, aggressive Aβ42 deposition [30]. COX-1 deletion preferentially enhances microglial clearance of Aβ42. In contrast, models with mixed Aβ40/42 [82] (e.g., APP/PS1) or tau-driven pathology [83] (3 × Tg-AD) may obscure the isoform-specific effects of COX-1. Moreover, the severe neuroinflammation in 5 × FAD mice provides a microenvironment where COX-1 ablation exerts adequate anti-inflammatory effects. In addition, we observed increased Arg1^+^Iba1^+^ microglia (anti-inflmmatory phenotype) in 5 × FAD/COX-1 KO mice, suggesting that COX-1 ablation reprograms microglial function rather than simply reducing Iba1^+^ cell numbers. Our innovative 5 × FAD/COX-KO system uniquely identifies COX-1 as a target for Aβ42-selective clearance. We propose that the effect of COX-1 KO in AD depends on both the Aβ isoform context and the inflammatory tonus, with 5 × FAD mice being an ideal platform to study Aβ42-centric therapeutics.

Furthermore, Aβ triggers NLRP3 inflammasome priming and activation. NF-κB enhances NLRP3 and pro-IL-1β transcription, leading to NLRP3 oligomerization and further activating IL-1β and TNF-α. NLRP3 inflammasome deficiency promotes Aβ clearance in AD mice [59, 84]. While our study focused on the Aβ-driven NLRP3 activation via the COX-1–PGE2–EP2 axis, it is important to note that multiple pathways contribute to NLRP3 inflammasome priming in AD. For instance, monoaminergic depletion (e.g., serotonin, dopamine) and tau pathology can independently activate NLRP3 through mitochondrial ROS or lysosomal damage [85]. Aspirin has shown potential for repurposing as an inhibitor of NLRP3 inflammasomes, which could be advantageous for AD treatment [86]. Inhibition of COX-1 could alleviate the adverse effects of hypobaric hypoxia on adult neurogenesis and social memory by reducing NLRP3 inflammasome-related inflammation [29]. Our research further revealed that deletion of COX-1 inhibited the NLRP3 inflammasome both in vitro and in vivo, indicating that COX-1 plays a crucial role in the regulation of NLRP3 inflammasomes within microglia.

COX-1 up-regulation could lead to an increase in PGs, such as PGE_2_ and PGD_2_, which may increase the formation of Aβ, morphological change, neuronal loss, and cognitive performance [87]. Targeting the PGE_2_/EP2 immune pathway has been proposed to restore microglial function and slow down AD progression [68, 88]. We demonstrated here that COX-1 deficiency inhibited activation of the PGE_2_/EP2 pathway in microglia of 5 × FAD mice. EP2 deficiency in microglia results in enhancement of Aβ phagocytosis and reduced Aβ neurotoxicity [89]. In addition, conditional deletion of EP2 in microglia restores Aβ clearance, and prevents synaptic injury and memory deficits in the APP-PS1 model [88], suggesting that the activation of EP2 may suppress microglia function in AD. Previous studies showed that the PGE_2_/EP2 receptor signaling increases IL-1β secretion by stimulating the cAMP/PKA signal pathway [90–92]. cAMP/PKA signaling exerts opposing biological effects in AD pathogenesis through spatiotemporally distinct mechanisms, neuroprotective in neurons versus pro-inflammatory in microglia. In neurons, enhancing cAMP/PKA signaling promotes synaptic plasticity and mitigates Aβ toxicity [93]. However, in microglia, the cAMP-driven PKA signaling exacerbates neuroinflammation by activating microglia [94]. Our findings highlight a microglial-specific maladaptive response mediated by the COX-1/PGE2/EP2 signaling, which highlights the therapeutic imperative for cell type-selective modulation of cAMP pathways.

Interestingly, PGE_2_ induced by intraperitoneal injection of *T. serrulatus* venom acts on EP2/EP4 receptors on macrophages to activate the cAMP –PKA –NFκB axis and trigger NLRP3 inflammasome activation, stimulating the release of IL-1β. This suggests that COX1/2 inhibition could be used as an effective therapeutic intervention for neuroinflammation associated with the cAMP–PKA–NFκB–NLRP3 axis [27, 28]. We demonstrated that the cAMP–PKA–NFκB axis was inhibited in 5 × FAD/COX-1 KO mice and BV2 cells treated with SC-560, a selective inhibitor of COX-1. Moreover, we showed that PGE_2_ activated NLRP3 inflammasomes to release IL-1β, which was inhibited by PKA and NFκB inhibitors. It is well known that NF-κB is a central mediator of the priming signal of NLRP3 inflammasomes [95]. However, there is very limited information on the mechansims of NFκB regulation of NLRP3 inflammasomes in microglia [96, 97]. In this study, we demonstrated that the cAMP–PKA–NFκB p65 axis is an important downstream pathway of PGE_2_/EP2 signaling to activate NLPR3 inflammasomes and then release IL-1β, which altered microglial polarization.

In this study, we found that deleting COX-1 reduced neuroinflammation and preserved cognitive function in 5 × FAD mice. Additionally, blocking PGE_2_/EP2 receptors mimicked the positive functions of COX-1 KO and slowed cognitive decline in 5 × FAD mice, indicating that PGE_2_/EP2 is involved in COX-1-related neuroinflammation. Moreover, our mouse model did not exhibit brain hemorrhages, likely due to compensatory mechanisms and cell-specific COX-1 expression patterns. These results underscore the potential of selectively targeting microglial COX-1/PGE_2_/EP2 signaling for AD therapy without exacerbating cerebrovascular risks. Unlike COX-1 inhibitors, EP2 antagonists selectively block prostaglandin E2 signaling without affecting TXA_2_ synthesis, thereby preserving platelet aggregation [98]. Our study demonstrated that the EP2 receptor functions as a crucial downstream factor in COX-1-mediated neuroinflammatory processes and presents another promising pharmacological target for mitigating the pathological effects associated with COX-1 activity in AD patients. EP2 inhibitors may be a better choice for AD treatment with lower bleeding risk.

Although preclinical evidence supports COX-1 inhibition as a viable strategy for mitigating Aβ pathology, clinical outcomes of aspirin in AD trials remain inconsistent. While the ASPREE trial found no cognitive benefit from low-dose aspirin over a median follow-up of 4.7 years [99], cohort studies suggest that high-dose aspirin significantly reduces AD prevalence and preserves cognitive function [100]. This discrepancy may reflect key mechanistic and methodological differences. Low-dose aspirin is primarily used for post-stroke prophylaxis, potentially masking anti-dementia effects due to competing vascular outcomes. However, low doses of aspirin may inadequately suppress neuroinflammation, whereas high doses can achieve sufficient CNS penetration to modulate COX-1-dependent pathways. Furthermore, longitudinal analyses indicate that ≥ 10 years of aspirin use correlate with reduced dementia risk, suggesting sustained COX-1 inhibition is critical for therapeutic efficacy [101]. Thus, the discrepancy of clinical results likely arise from dfferent doses and durations, underscoring the need for tailored therapeutic strategies in future trials.

We acknowledge several limitations that warrant future investigation. First, our 5 × FAD/COX-1 KO model precludes cell-type-specific resolution. Future work will focus on developing tamoxifen-inducible *Ptgs1* conditional knockout mice (Cx3cr1-CreERT2;Ptgs1fl/fl) to temporally and spatially ablate COX-1 in microglia to precisely evaluate the interactions with astrocytes and neurons. Second, the precise molecular mechanisms of COX-1-dependent Aβ clearance remain unclear. Particularly, little is known on the interactions between prostaglandin metabolites (e.g., PGE2-EP2 signaling) and phagocytic receptors (TREM2/CD36). Finally, only male 5 × FAD mice were used in this study, which limited the generalizability of our findings given the documented sex-dimorphic microglial responses. Future studies will include age-matched female mice to assess sexual dimorphism in COX-1-mediated neuroprotection. Such comparative analyses will not only refine therapeutic strategies but also lay critical groundwork for clinical translation of COX-1-targeted interventions.

## Conclusions

**Fig. 11:**
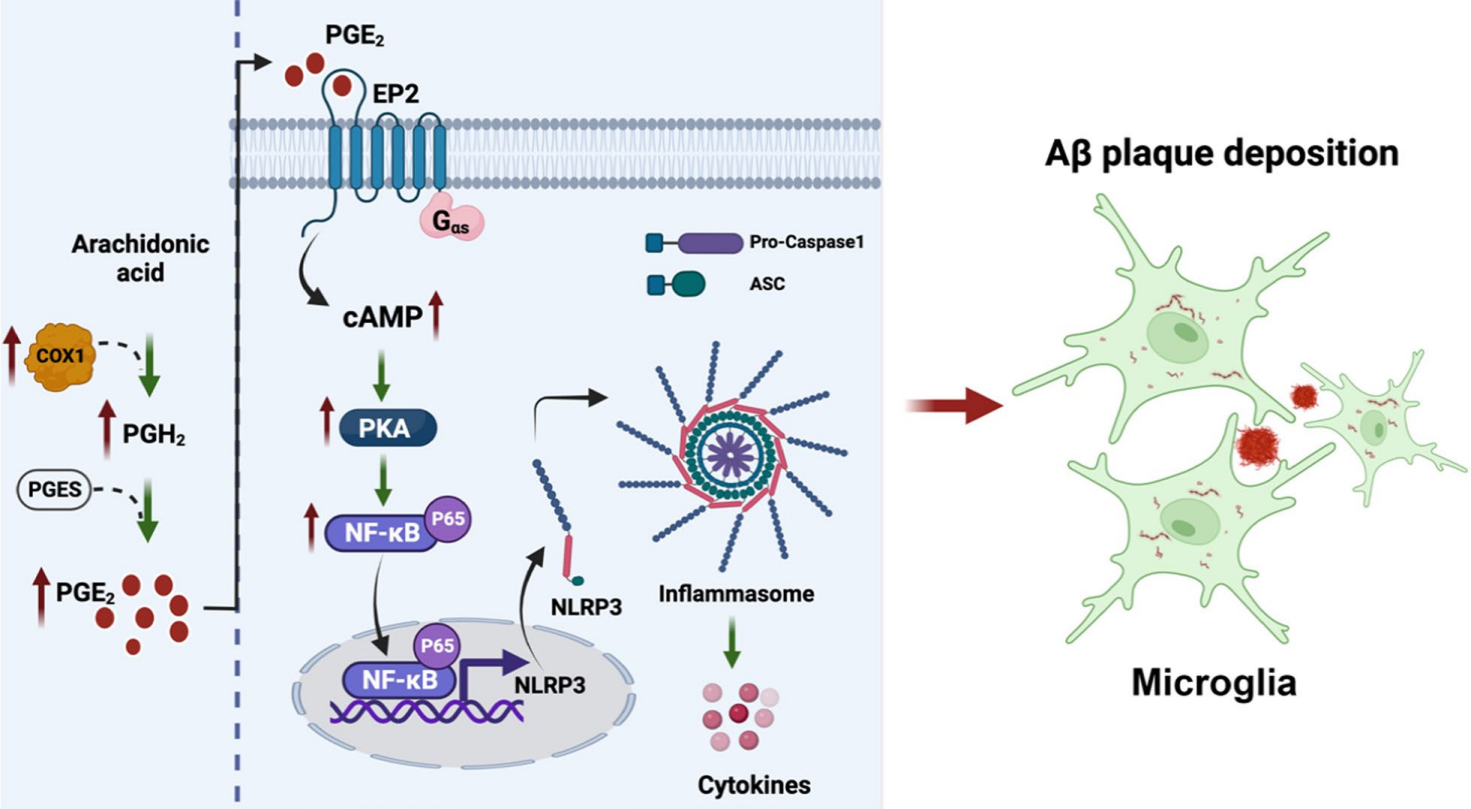
Involvement of COX-1 in neuroinflammation in AD. COX-1 level increases remarkably in AD progression, which promotes Aβ deposition and exacerbates neuroinflammation via the PGE_2_/EP2 pathway, resulting in activation of the cAMP–PKA–NFκB p65 axis and NLRP3 inflammasome activation

In this study, we identified the COX-1/PGE2/EP2 signaling pathway as a key contributor to chronic neuroinflammation in 5 × FAD mice through activation of the cAMP–PKA–NFκB p65 axis (Fig. 11). Importantly, COX-1 signaling can be pharmacologically suppressed by an EP2 inhibitor, indicating a novel target for anti-inflammatory therapies and promising clinical applications in AD. Pharmacological targeting of inflammatory EP2 signaling may effectively mitigate the pathological activity of COX-1, thereby offering therapeutic benefits for AD patients with minor adverse effects.

## Supplementary Information

**Additional file 1**. **Supplementary methods**. **Table S1** Primers for quantification of gene expression. **Figure S1**. Single-cell expression profiling of COX-1 in different cell types by using ssREAD. **Figure S2**. Expression of COX1/2 in AD and characterization of COX-1 expression in 5×FAD/COX-1 KO mice. **Figure S3**. Cerebral hemorrhage and safety assessments in 5×FAD/COX-1 KO mice. **Figure S4**. Levels of other PGs in the hippocampus, mRNA expression of DP/EP receptors, and levels of NF-κB/NLRP3 pathway-related proteins.

**Additional file 2**. **Table S2** Quantitative analysis of COX-1 expression between microglia and other cell types from ssREAD.

**Additional file 3**. **Table S3** Quantitative analysis of gene expressions among different clusters of microglia from ssREAD database.

**Additional file 4**. Uncropped Western blots.

## Data Availability

All data analyzed and presented in this study are available from the corresponding author on reasonable request.

## Abbreviations

AD: Alzheimer's disease
COX1: Cyclooxygenase 1
COX2: Cyclooxygenase 2
Aβ: Amyloid-beta
PGs: Prostaglandins
PGE_2_: Prostaglandin E_2_
cAMP: Cyclic adenosine monophosphate
NF-κB: Nuclear factor-κB
PKA: Protein kinase A
NLRP3: NOD-like receptor protein 3
APP: Amyloid precursor protein
DAM: Disease-associated microglia
TREM2: Triggering receptor expressed on myeloid cells 2
Arg-1: Arginase-1
NSAIDs: Non-steroidal anti-inflammatory drugs
IL-4: Interleukin-4
IL-1β: Interleukin-1β
LPS: Lipopolysaccharide
PFA: Paraformaldehyde
FBS: Fetal bovine serum
GFAP: Glial fibrillary acidic protein
Thio-S: Thioflavin S staining
OFT: Open field test
NOR: Novel object recognition
MWM: Morris water maze
RIPA: Radioimmunoprecipitation
DG: Dentate gyrus
HPLC: High-performance liquid chromatography
MS: Mass Spectrometry
NeuN: Neuronal nuclei
ASC: Apoptosis-associated speck-like protein
UMAP: Uniform manifold approximation and projection
PGH_2_: Prostaglandin H2
GPRs: G-protein coupled receptors
GEO: Gene Expression Omnibus
MCI: Mild cognitive impairment
BBB: Blood–brain barrier
ALT: Alanine aminotransferase
AST: Aspartate aminotransferase
TREM2: Triggering receptor expressed on myeloid cells-2
GLUL: Glutamate-ammonia ligase
S100A: S100 calcium-binding protein A
DG: Dentate gyrus
GM-CSF: Granulocyte–macrophage colony-stimulating factor
