# Supplementary material for: Cyclooxygenase-1 deletion in 5 × FAD mice protects against microglia-induced neuroinflammation and mitigates cognitive impairment
Source: Transl Neurodegener. 2025 Aug 22;14:43. doi: 10.1186/s40035-025-00501-9 (PMC12372357; doi:10.1186/s40035-025-00501-9)
Supplement: Supplementary file 1 — Additional file 1. Supplementary methods. Table S1 Primers for quantification of gene expression. Figure S1. Single-cell expression profiling of COX-1 in different cell types by using ssREAD. Figure S2. Expression of COX1/2 in AD and characterization of COX-1 expression in 5×FAD/COX-1 KO mice. Figure S3. Cerebral hemorrhage and safety assessments in 5×FAD/COX-1 KO mice. Figure S4. Levels of other PGs in the hippocampus, mRNA expression of DP/EP receptors, and levels of NF-κB/NLRP3 pathway-related proteins. [file 40035_2025_501_MOESM1_ESM.docx]

**Supplementary methods**

**Genotyping**

Genotyping was performed as previously described [1]. The tails of mice (21 days after birth) were cut off (3-5 mm in length) for genetic identification. Total DNA was isolated from the tails using the DNA extraction kit (Beijing Tiangen Biochemical Technology, China) and then PCR was performed. For APP/PS1 the following protocol was used: initial denaturation for 5 min at 95℃, followed by 35 cycles of denaturation for 30 sec at 95℃, annealing for 30 sec at 66℃, and extension for 30 sec at 72℃, followed by final extension for 10 min at 72℃. Primers (Sangon Biotech, China) used for PCR of 5×FAD mice are set as following. PS1 forward: 5’-AATAGAGAACGGCAGGAGCA-3’, PS1 reverse: 5’-GCCATGAGGGCACTAATCAT-3’. WT forward: 5’-CTAGGCCACAGAATTGAAAGATCT-3, WT reverse: 5’-GTAGGTGGAAATTCTAGCATCATCC-3. For the COX-1KO, the samples were incubated at 94°C for 4 min, followed by 32 cycles ( denaturation at 94°C for 50 sec, annealing at 62°C for 50 sec, and elongation at 72°C for 2 min), followed by a final extension for 5 min at 72℃, 4℃ store. The following primers (Sangon Biotech, China) were used: CDF 141:5'-ATCGCCTTCTTGACGAGTTC-3', CDF eric:5’-CATGAGTGACTGCGTCCTCTTG-3’, and COX1 KI probe2: 5’-TCAGAGCTCAGTGGAGCGT-3’. The PCR products were run on 1% agarose gels made with TAE bugger for 30min (140 V constant voltage) and EB exposed in the exposure instrument (Bio-Rad, USA) to observe the gene bands (Fig. S2e and f).

Reference

[1] Yang Y, Wang J, Ni H, Ding H, Wei L, Ke ZJ. Genetic model of selective COX2 inhibition improve learning and memory ability and brain pathological changes in 5×FAD mouse. Brain Res. 2023;1821:148566.

**Table S1 Primers for quantification of gene expression**

| Gene | Forward | | Reverse |
| --- | --- | --- | --- |
| *Cox-1* | 5′-GATTGTACTCGCACGGGCTAC-3′ | 5′-GGATAAAGGTTGGAVVGCACT-3′ | |
| *Cox2* | 5′-TGCACTATGGTTACAAAAGCTGG-3′ | 5′-TCAGGAAGCTCCTTATTTCCCTT-3′ | |
| *Il-1β* | 5′-ATCTCGCAGCAGCACATCAAC-3′ | 5′-TGTTCATCTCGGAGCCTGTAGT-3′ | |
| *Nlrp3* | 5′-GACACGAGTCCTGGTGACTTT-3′ | 5′ -GATGATGTTGGCAGCAATGG-3′ | |
| *Gapdh* | 5′-GCCAAATTCAACGGCACAGT -3′ | 5′-AGATGGTGATGGGCTTCCC-3′ | |
| *Dp-1* | 5′-AACCTCTATGACATGCACAGGCG-3′ | 5′-AAGGCTTGGAGGTCTTCTGAGTC-3′ | |
| *Dp-2* | 5′-TCTCAACCAATCAGCACACCCGA-3′ | 5′-TCTCAACCAATCAGCACACCCGA-3′ | |
| *Ep-1* | 5′-CGCAGGGTTCACGCACACGA-3′ | 5′-CACTGTGCCGGGAACTACGC-3′ | |
| *Ep-2* | 5′-AGGACTTCGATGGCAGAGAGAC-3′ | 5′-CAGCCCCTTACACTTCTCCAATG-3′ | |
| *Ep-3* | 5′-AGGGAAATGATGGCACCA-3′ | 5′-GCTGTCCGTCTGTTGGTC-3′ | |
| *Ep-4*  *Tnf-α*  *iNOS*  *Il-6*  *Il-1α*  *Cd206*  *Il-10*  *Il-13*  *Il-4* | 5′-TGGCTGAGGTTGGAGGTA-3′  5′-TACTGAACTTCGGGGTGATTGGTCC-3′  5′-GATAAAGGGACAGCGTCAGC-3′  5′-GCTACAGCACAAAGCACCTG-3′  5′-AAGGAGAGCCGGGTGACAGT-3′  5′-ACTGCGTGGTGATGAAAGG-3′  5′-TTGAACCACCCGGCATCTAC-3′  5′-CCTGGCTCTTGCTTGCCTT-3′  5′-CGTGATGTACCTCCGTGCTT-3′ | 5′-GAAGTAGGCGTGGTTGAT-3′  5′-CAGCCTTGTCCCTTGAAGAGAACC- 3′  5′-CCTTCGGGCCAAAGATCCTG-3′  5′-GACTTCAGATTGGCGAGGAG-3′  5′-GAAACTCAGCCGTCTCTTCTTCA-3′  5′-TAACCCAGTGGTTGCTCACA-3′  5′-CCAAGGAGTTGCTCCCGTTA-3′  5′-GGTCTTGTGTGATGTTGCTCA-3′  5′-GTGAGTTCAGACCGCTGACA-3′ | |

**
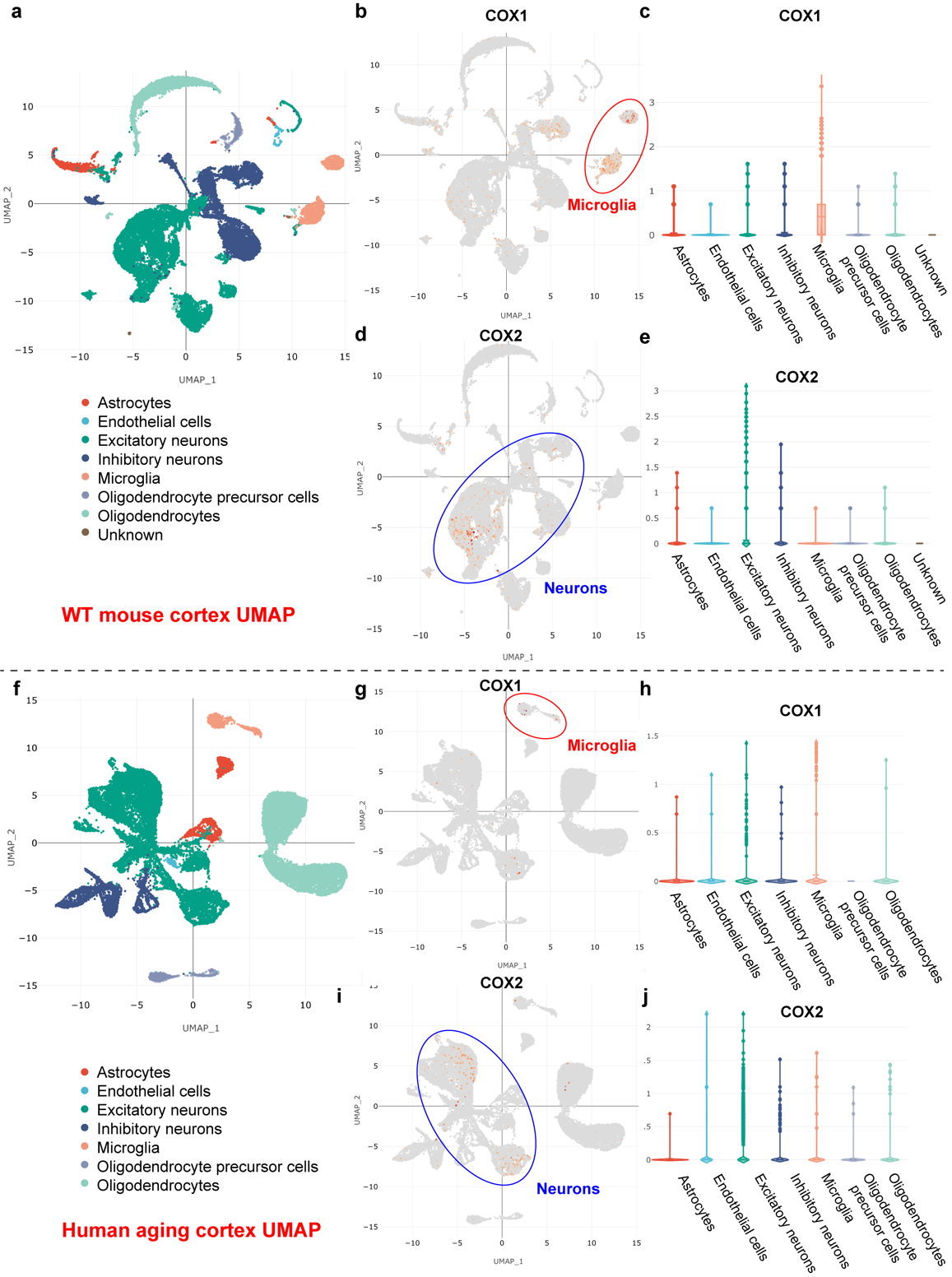
**

**Figure S1. Single-cell expression profiling of COX-1 in different cell types by using ssREAD. a** UMAP visualization and unsupervised clustering of brain cells derived from WT mice (Data ID: AD00301, male, 7 months old). Colors represent cluster identity. **b** Expression of COX-1 in different subpopulations of brain cells. **c** Average expression of the COX-1 across clusters. **d** Expression of COX-2 in different subpopulations of brain cells. **e** Average expression of COX-2 across clusters. **f** UMAP visualization and unsupervised clustering of brain cells derived from aging patients (Data ID: AAD00101, female, 75-90 years). Colors represent cluster identity. **g** Expression of COX-1 in different subpopulations of brain cells. **h** Average expression of the COX-1 across clusters. **i** Expression of COX-2 in different subpopulations of brain cells. **j** Average expression of COX-2 across clusters.


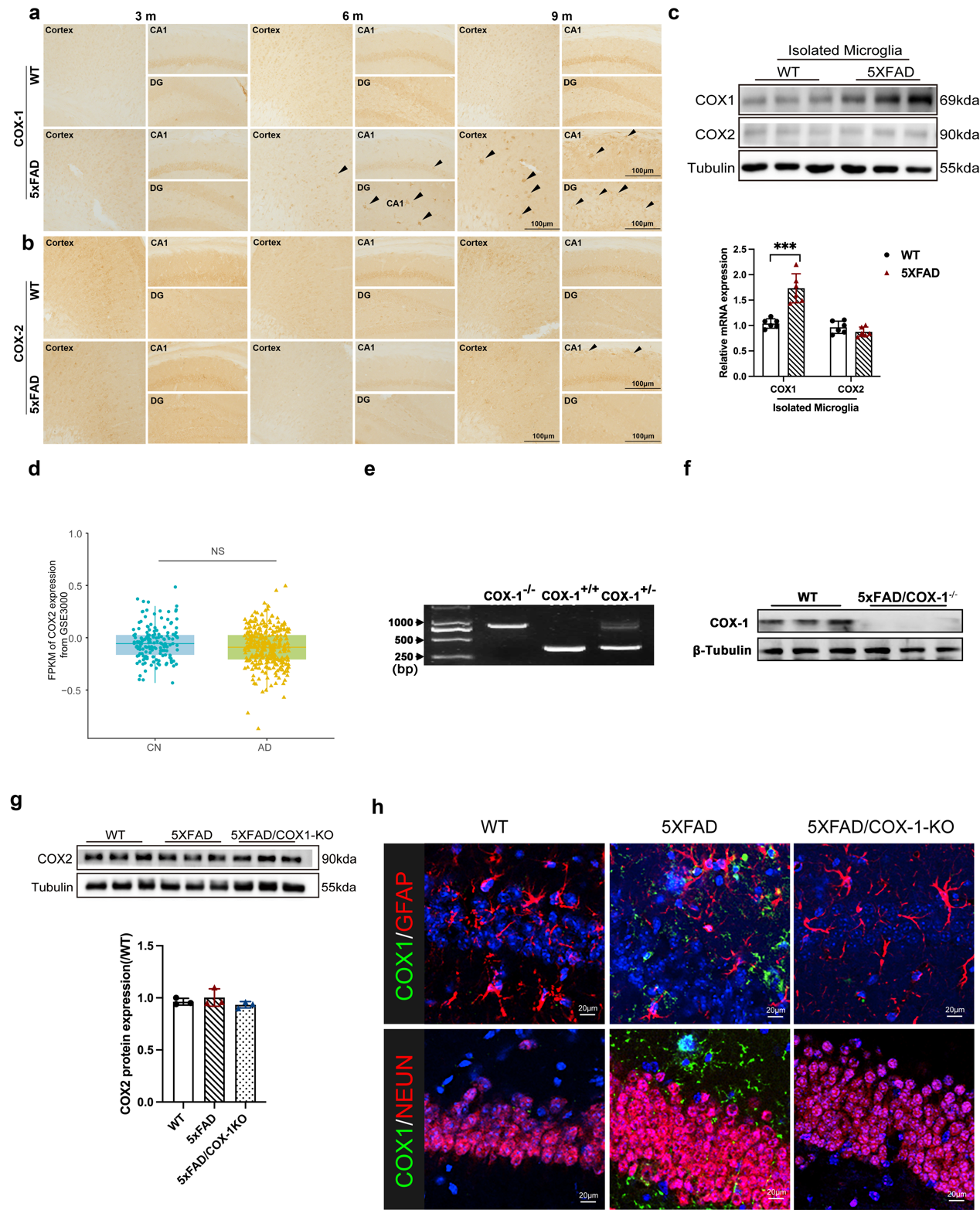


**Figure S2. Expression of COX1/2 in AD and characterization of COX-1 expression in 5×FAD/COX-1 KO mice**. **a** COX1 expression in the cortex, CA1 and DG regions of WT and 5×FAD mice at different age (3-, 6-, and 9-month-old) (n=5); **b** COX2 expression in the cortex, CA1 and DG regions of WT and 5×FAD mice at different age (3-, 6-, and 9-month-old) (n=5); **c** COX1 and COX2 protein expression in isolated microglia from WT and 5×FAD mice (n=3). **d** COX2 expression from GEO database (GSE3000) between controls and AD humans. **e** Genotyping of COX-1 KO mice by PCR. The PCR product of COX1 was 900 bp. **f** COX-1 protein expression in the hippocampus of WT and 5×FAD/COX-1 KO mice detected by Western blot (n=3); **g** COX-2 protein expression in the hippocampus of WT, 5**×**FAD and 5**×**FAD/COX-1 KO mice (n=3); **h** Representative double immunofluorescence staining of COX-1 (green) and GFAP/NEUN (red) in the cortex of WT, 5**×**FAD and COX-1 KO mice at 9 months old**.** Means ± SEM; ns: nonstatistical significance; ^***^ *P* < 0.001. Scale bar = 20μm.


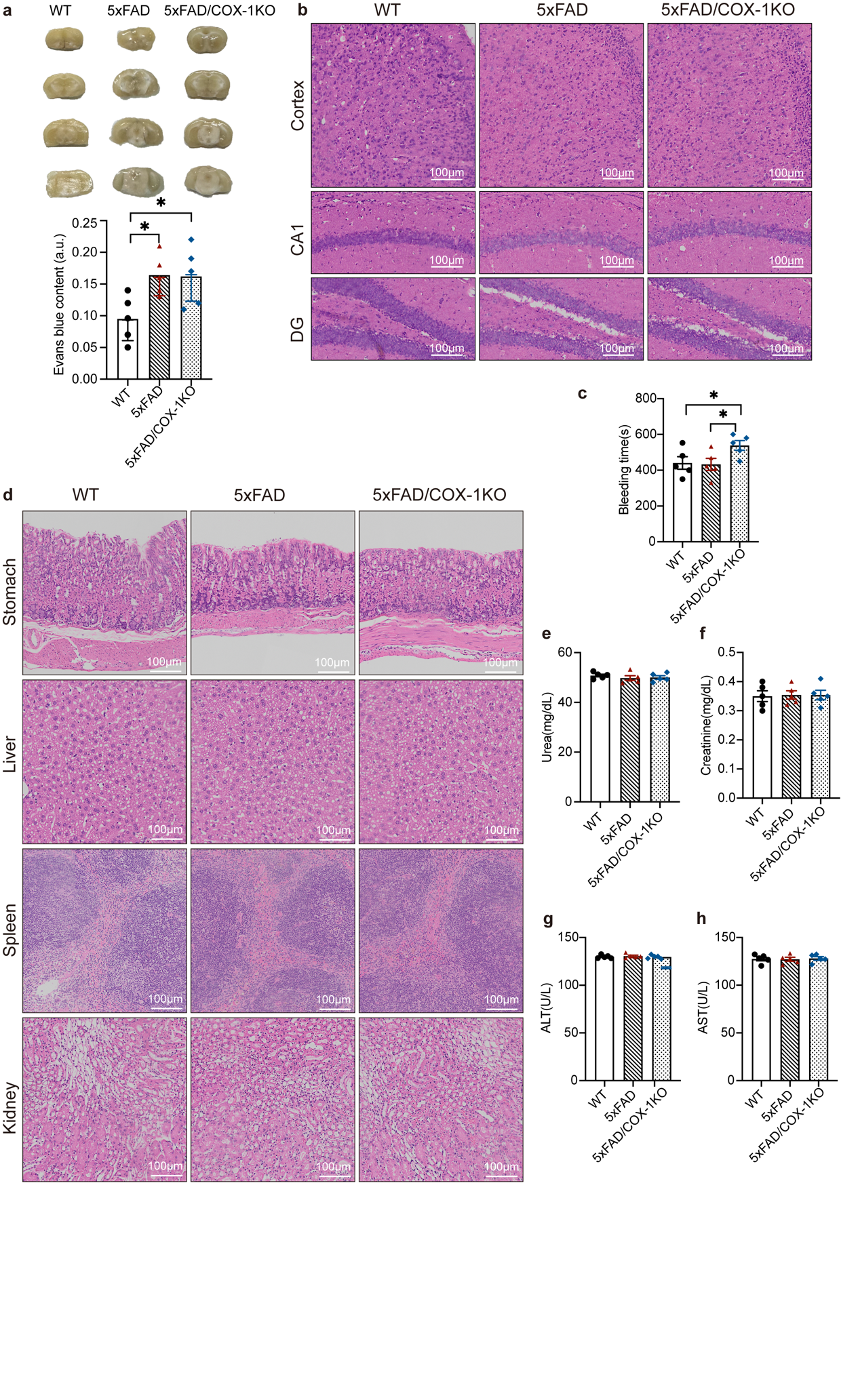


**Figure S3.** **Cerebral hemorrhage and safety assessments in 5×FAD/COX-1 KO mice.** **a** Evans blue staining and quantification of Evans blue dye, normalized to serum dye content and brain tissue weight; **b** Representative HE staining in different brain regions of WT, 5**×**FAD and 5**×**FAD/COX-1 KO mice at 9 months old; **c** Tail bleeding time of WT, 5**×**FAD and 5**×**FAD/COX-1 KO mice; **d** Representative HE staining of stomach, liver, spleen, and kidney tissues of WT, 5**×**FAD and 5**×**FAD/COX-1 KO mice at 9 months old; **e** The levels of Urea (**e**), Creatinine (**f**), ALT (**g**), and AST(**h**) in the serum of WT, 5**×**FAD and 5**×**FAD/COX-1 KO mice at 9 months old; n=5; Means ± SEM; ns: nonstatistical significance; * *P* < 0.05. Scale bar = 100 μm.

**
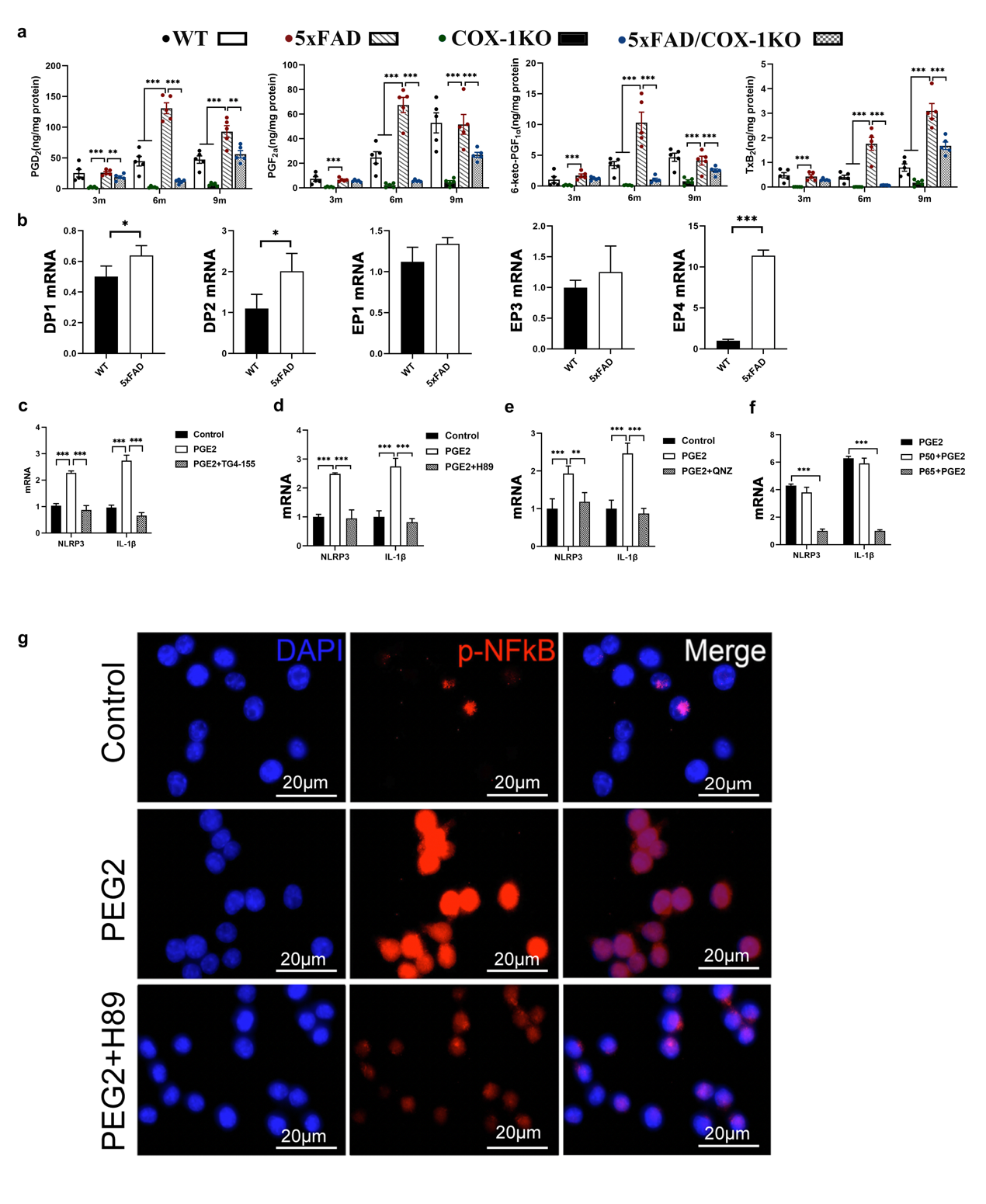
**

**Figure S4.** **Levels of other PG_S_ in the hippocampus, mRNA expression of DP/EP receptors, and levels of NF-κB/NLRP3 pathway-related proteins. a** Protein levels of PGD_2_, 6-keto-PGF_1a_, PGF_2a_, and TxB_2_ measured by HPLC/mass spectrometry in the hippocampus of WT, COX-1KO, 5×FAD and 5×FAD/COX-1KO mice at 3-, 6-, and 9-month-old. **b** mRNA Expression of DP-1, DP-2, EP-1, EP-3, and EP-4 in the hippocampus of WT and 5×FAD mice at 9 months old. **c-f** mRNA expression of NLRP3 and IL-1β in BV2 cells treated with PGE_2_ and TG4-155 (**c**), H89 (**d**), QNZ (**e**), or siRNA for NF-κB p65 or p50 (**f**). **g** Representative double immunofluorescence staining of p-NF-κB (red) and DAPI (blue) in BV2 cells in different groups (n=3). Means ± SEM; n = 5 per group; * *P* < 0.05, ** *P* < 0.01, *** *P* < 0.001.
