# Supplementary material for: Cyclooxygenase-1 deletion in 5 × FAD mice protects against microglia-induced neuroinflammation and mitigates cognitive impairment
Source: Transl Neurodegener. 2025 Aug 22;14:43. doi: 10.1186/s40035-025-00501-9 (PMC12372357; doi:10.1186/s40035-025-00501-9)
Supplement: Supplementary file 4 — Additional file 4. Uncropped Western blots. [file 40035_2025_501_MOESM4_ESM.docx]

**Original blots of COX-1 of WT and 5×FAD mice at 3-, 6-, and 9-month-old (Corresponding to Fig. 3)**

Cox-1 β-Tubulin


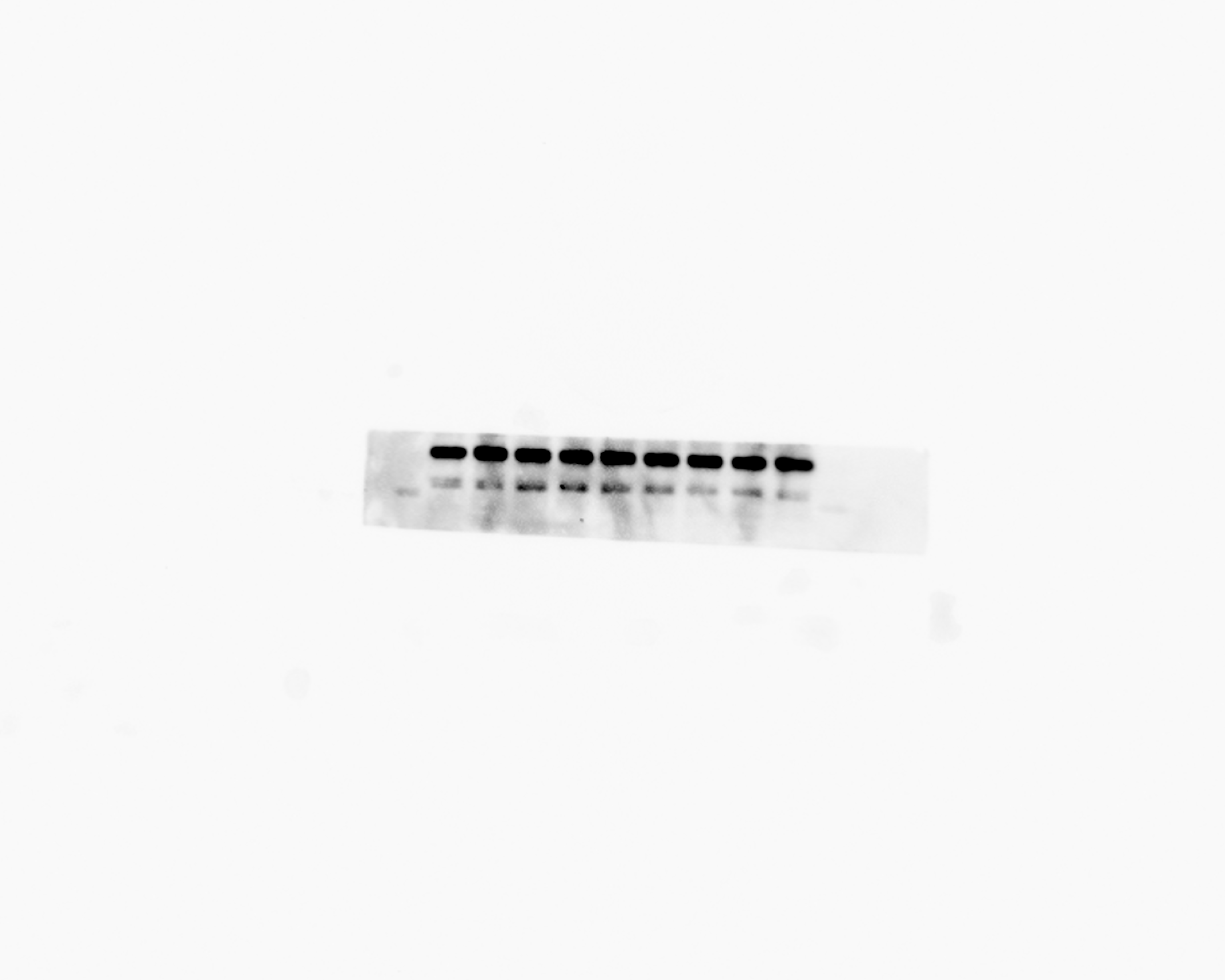

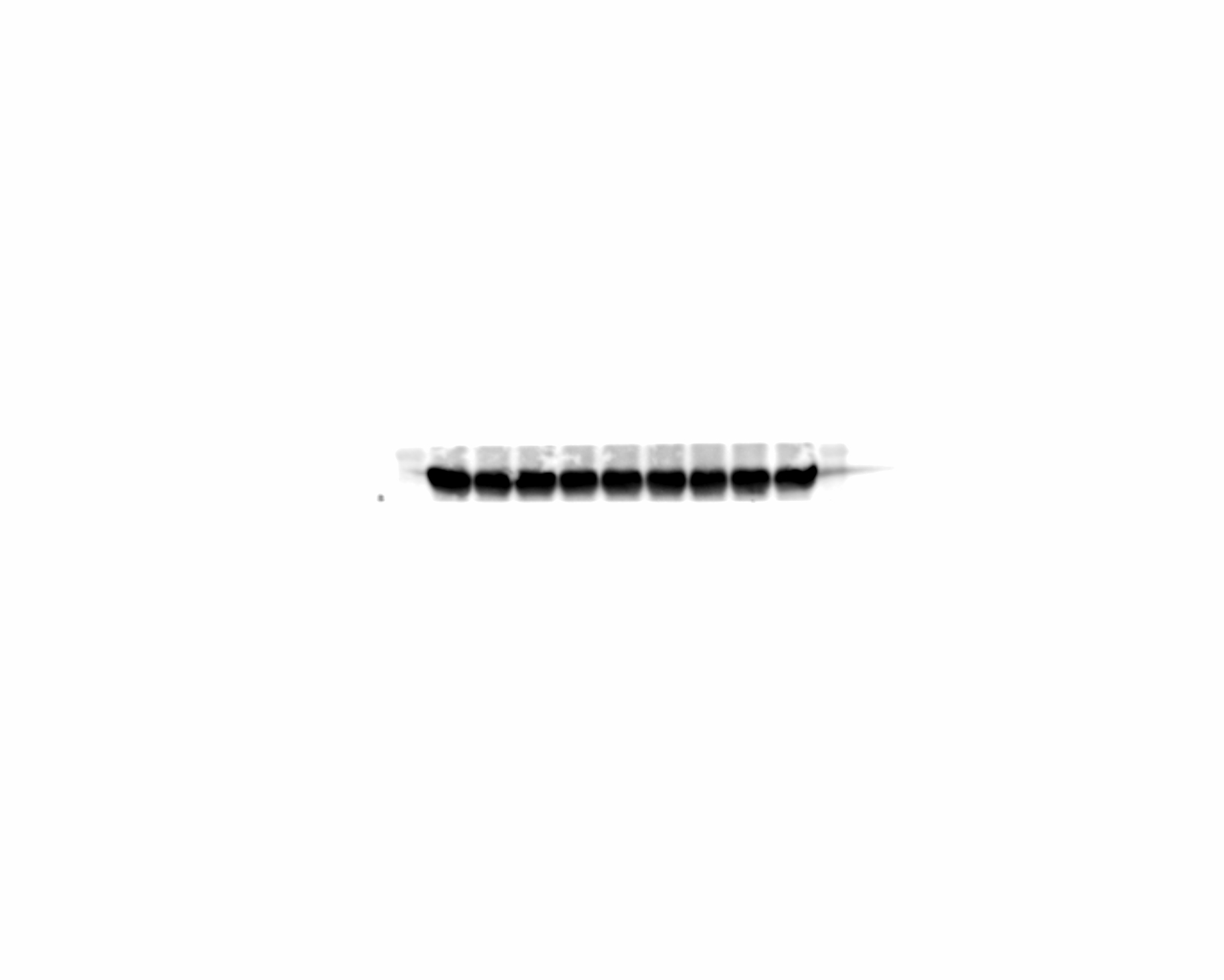


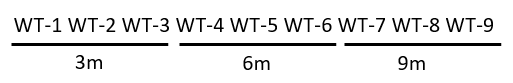

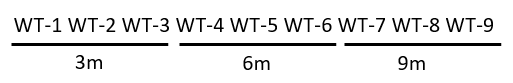


Cox-1 β-Tubulin


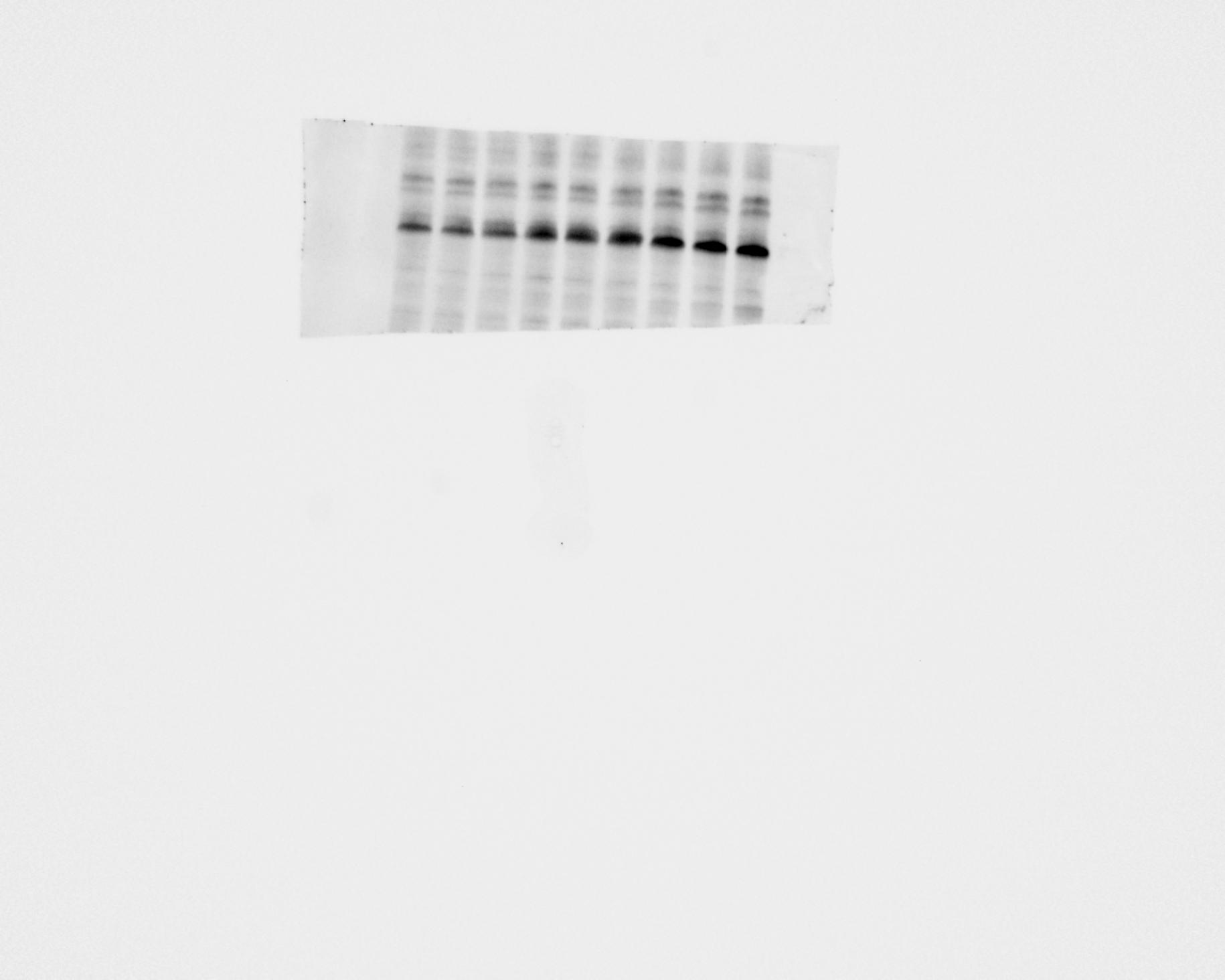

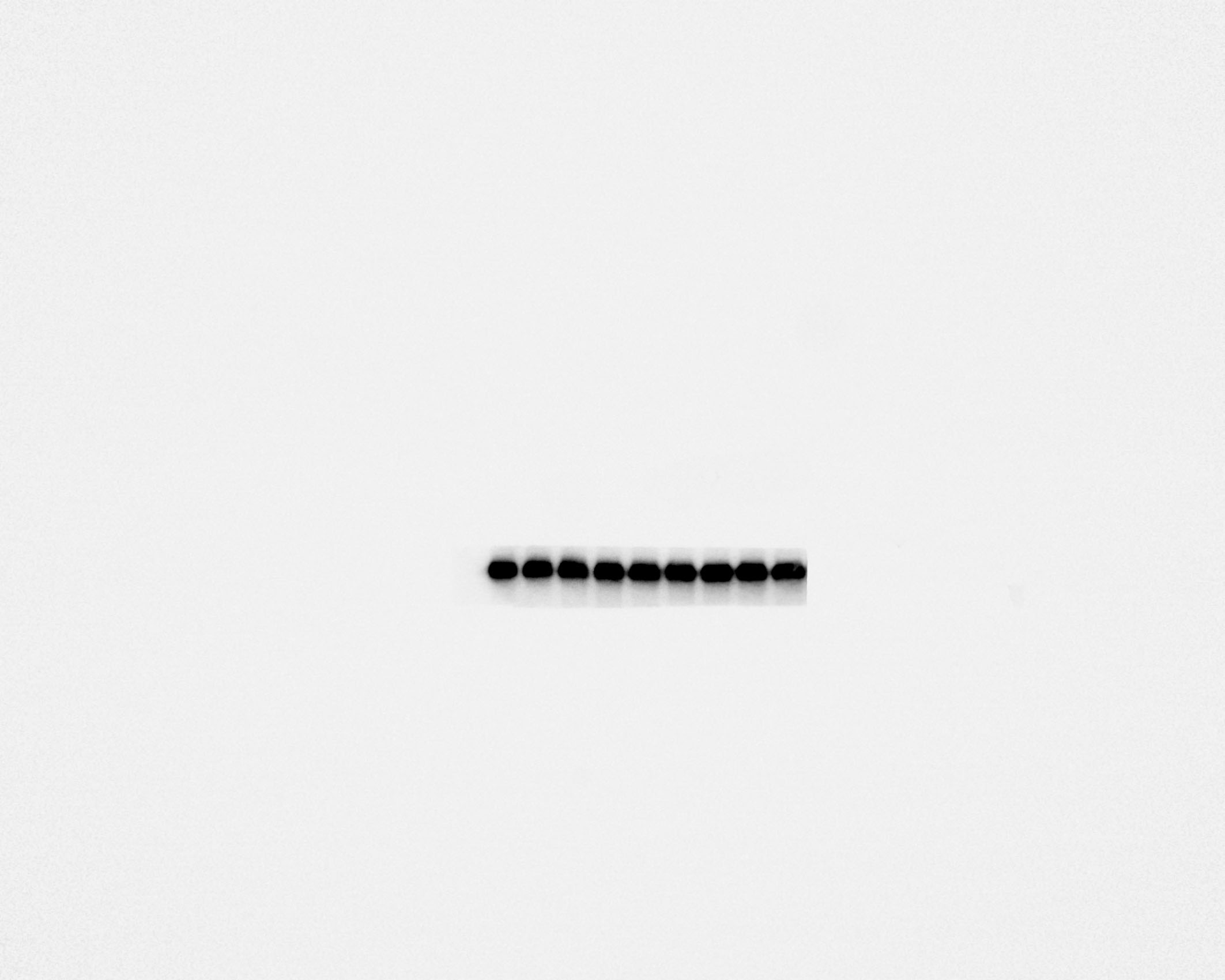


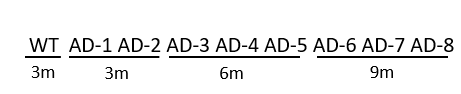

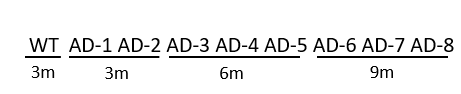


**Original blots of EP-2, p-PKA, PKA, NLRP3, NF-kB P65, ASC, Casepase-1, IL-1β, β-Tubulin of WT, 5×FAD, and 5xFAD/COX-1KO mice at 9-month-old (Corresponding to Figure 8).**

**
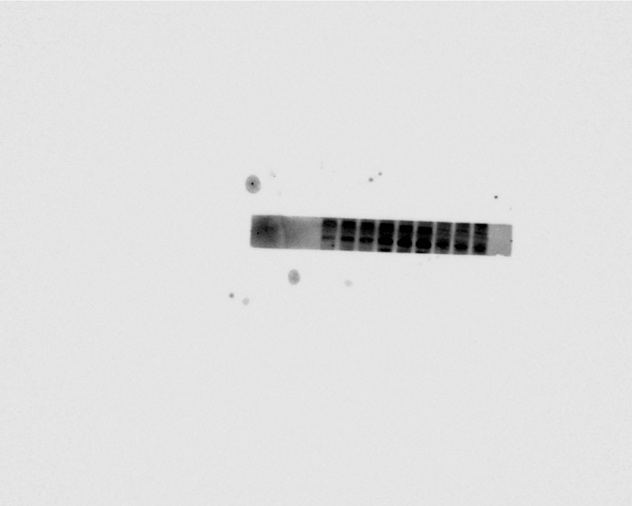
**EP-2 β-Tubulin

**
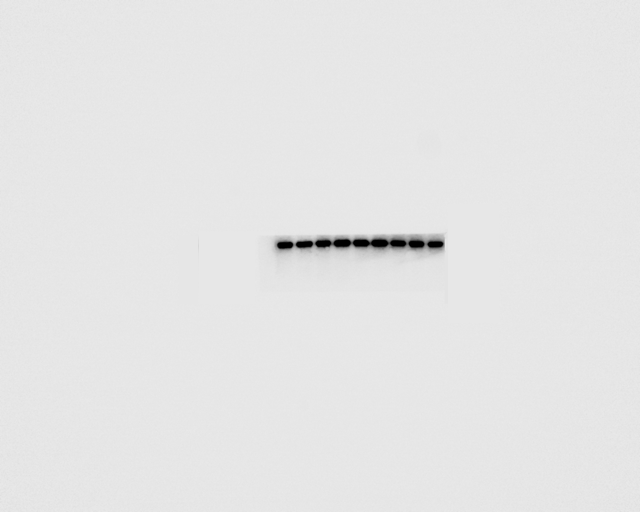
**


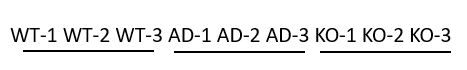

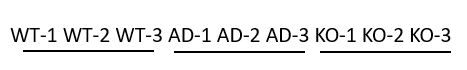


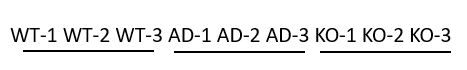

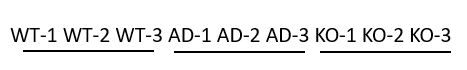

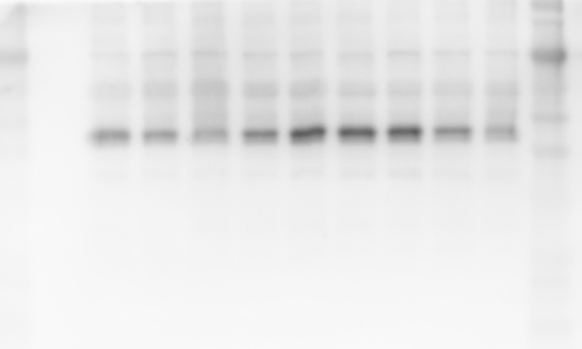
p-PKA PKA


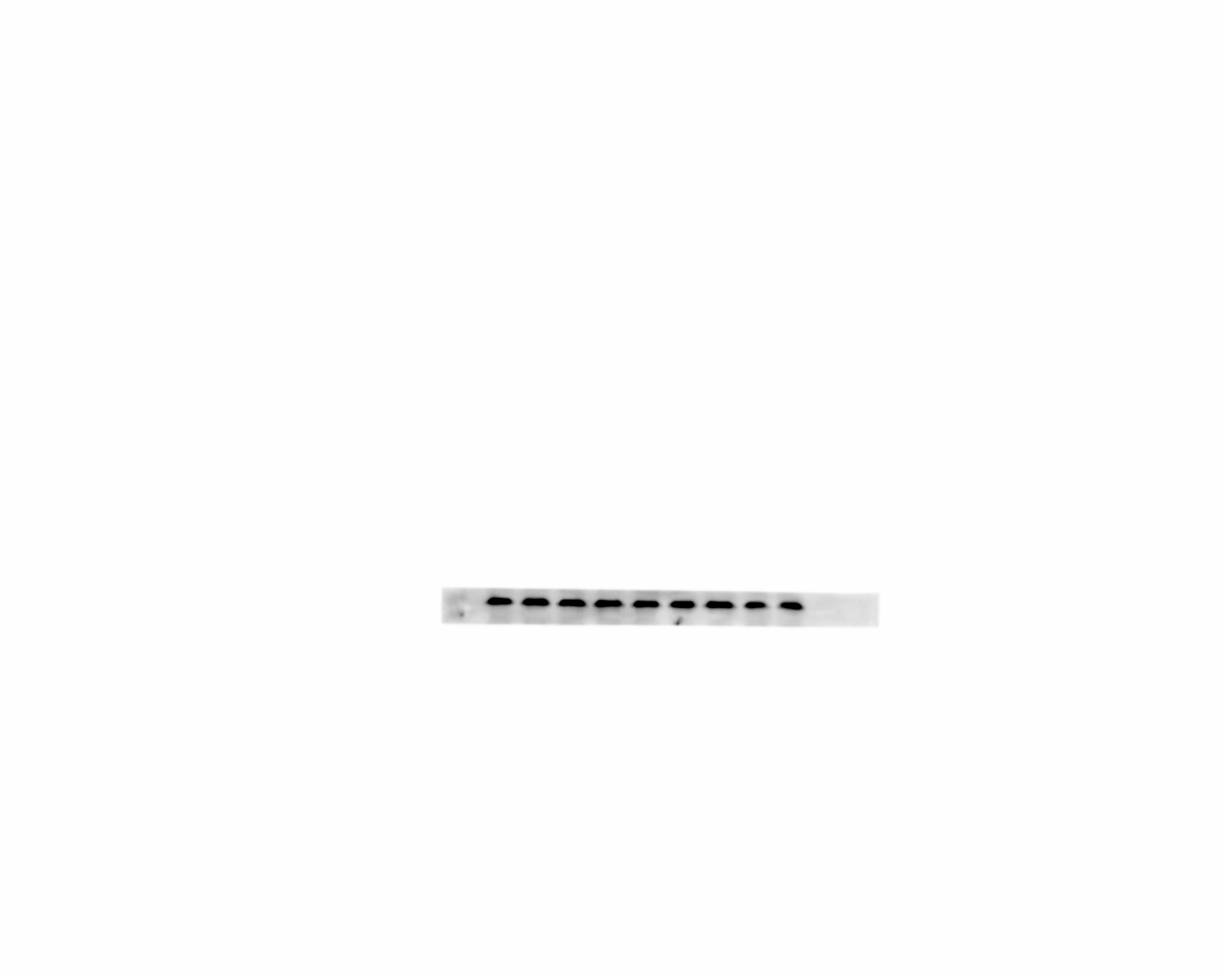


NLRP3 NF-kB p65


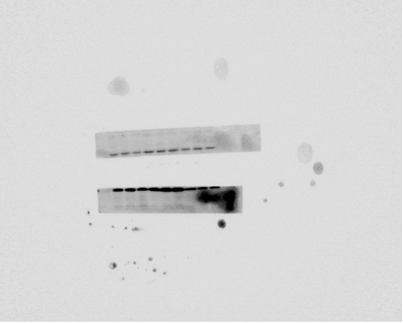


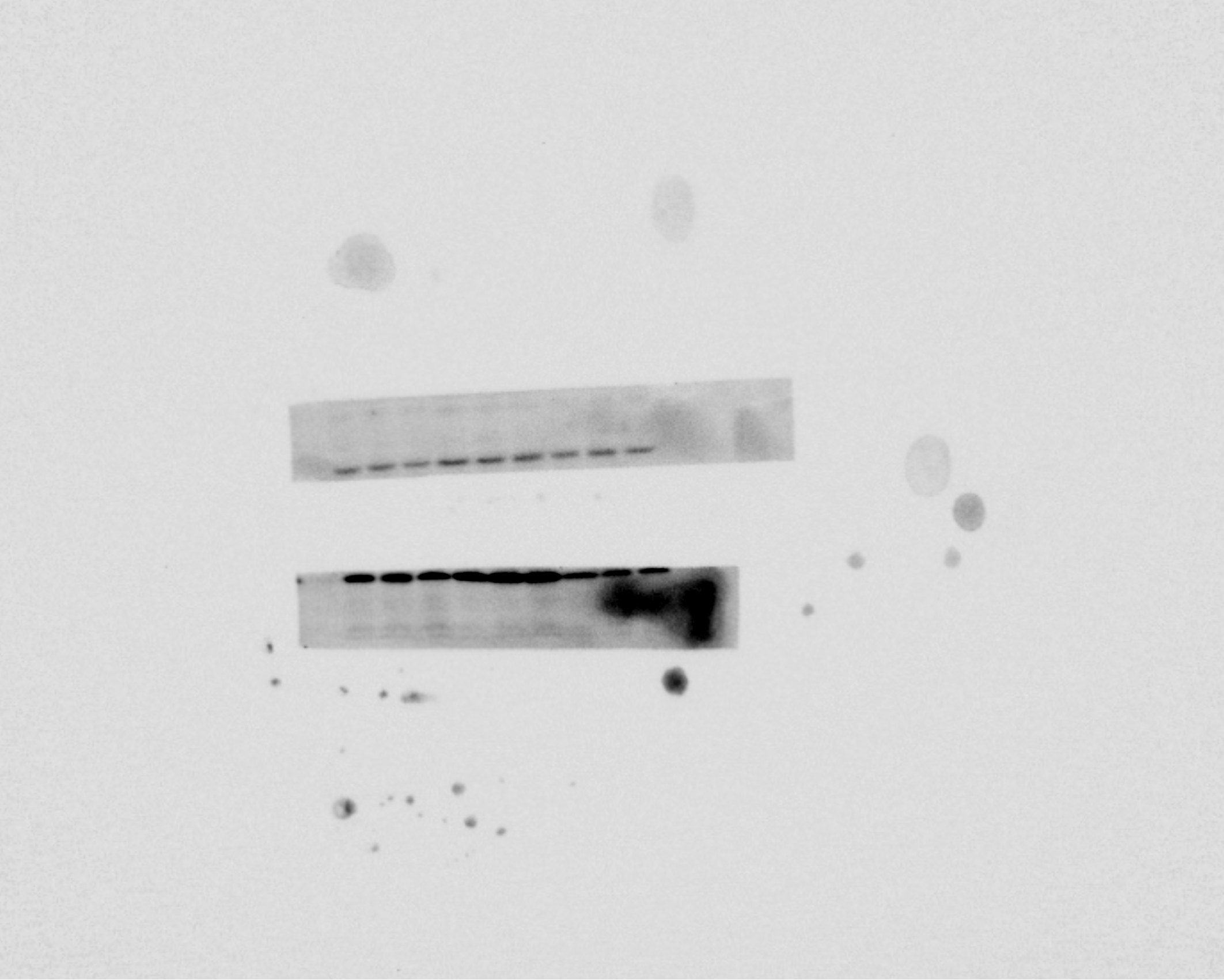


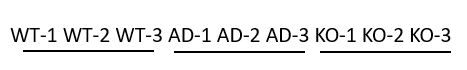

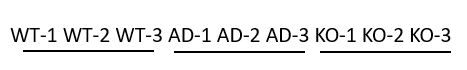


ASC Caspase-1


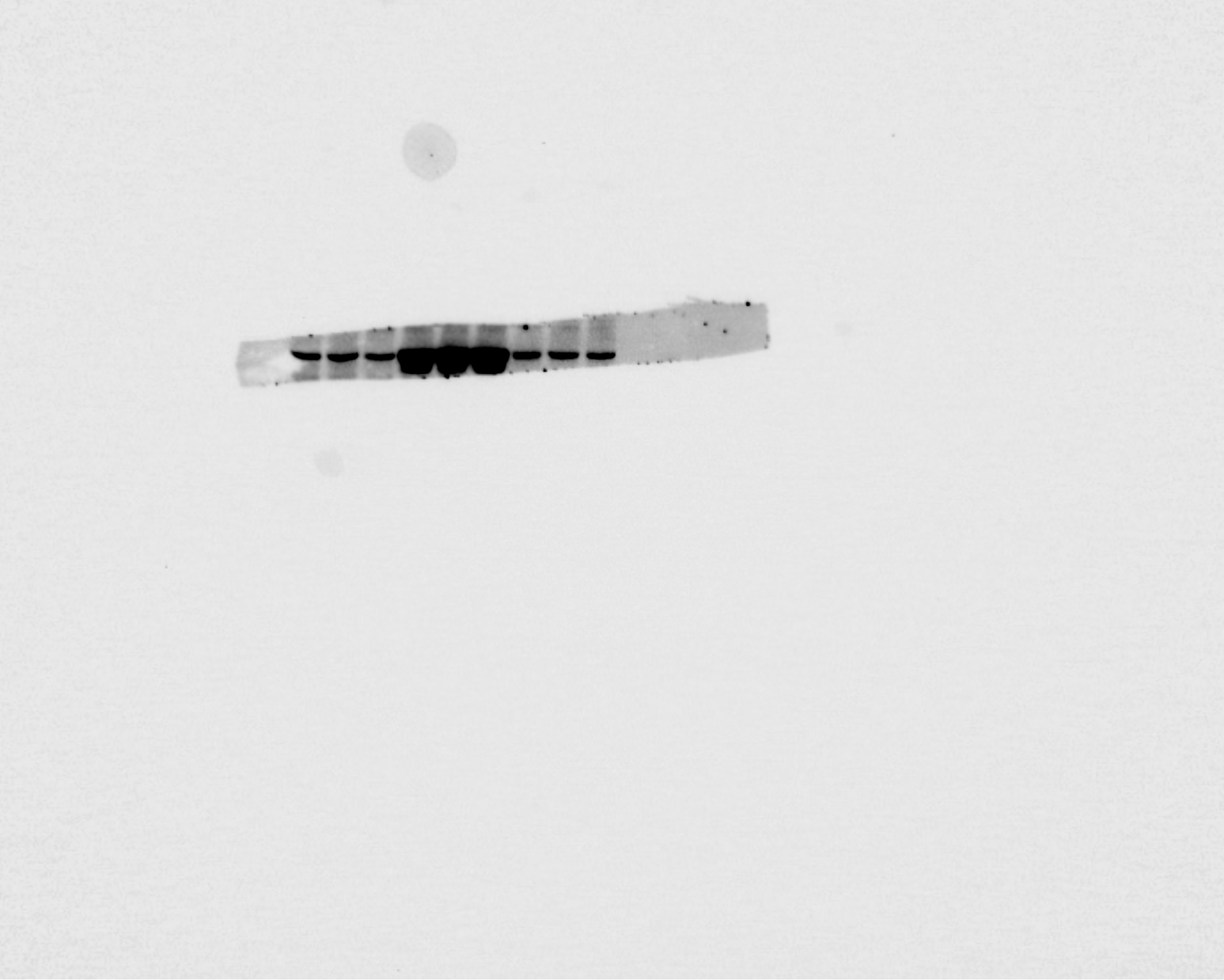

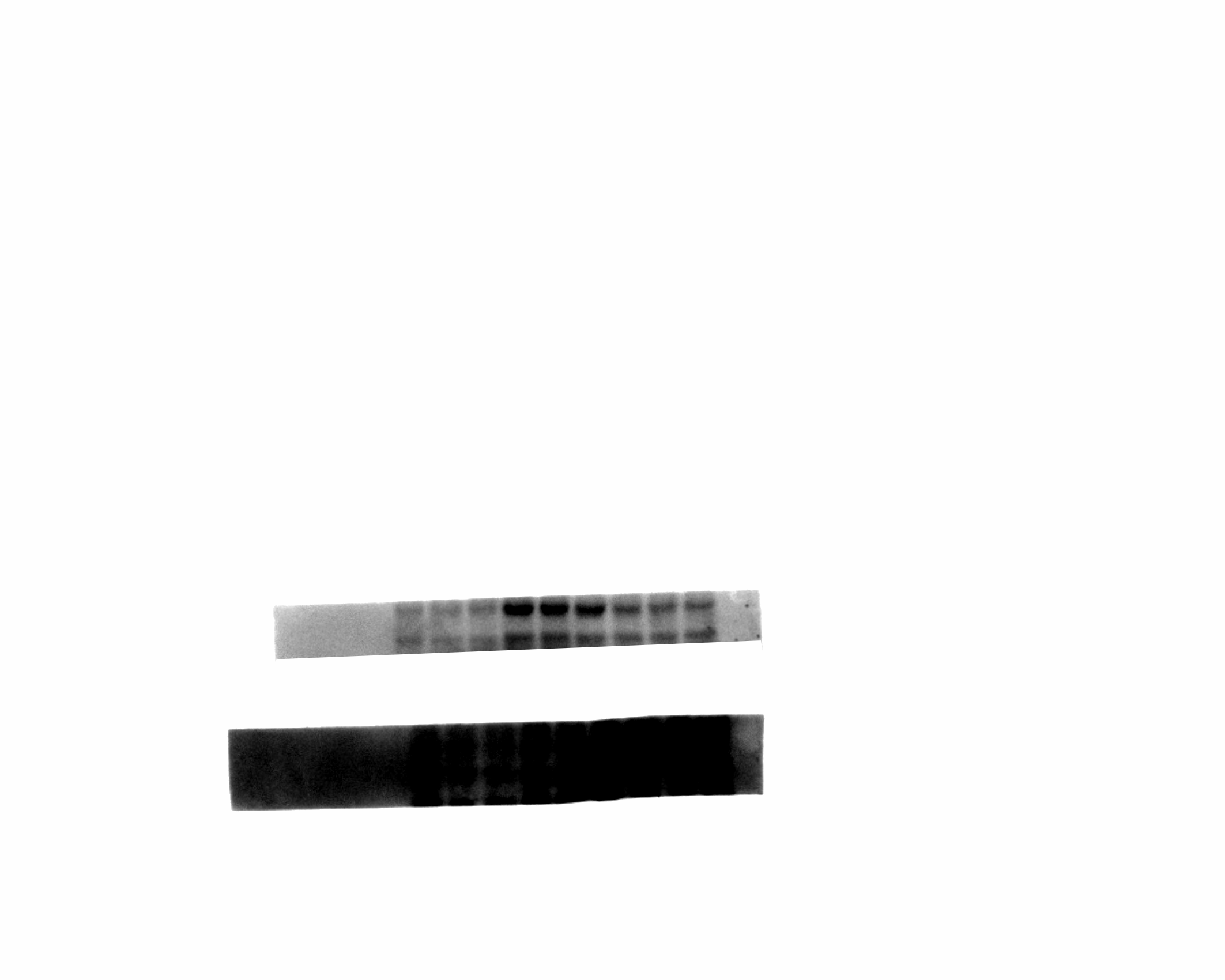


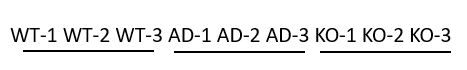

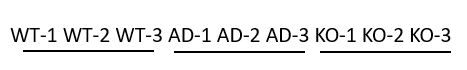


IL-1β β-Tubulin

IL-1β β-Tubulin


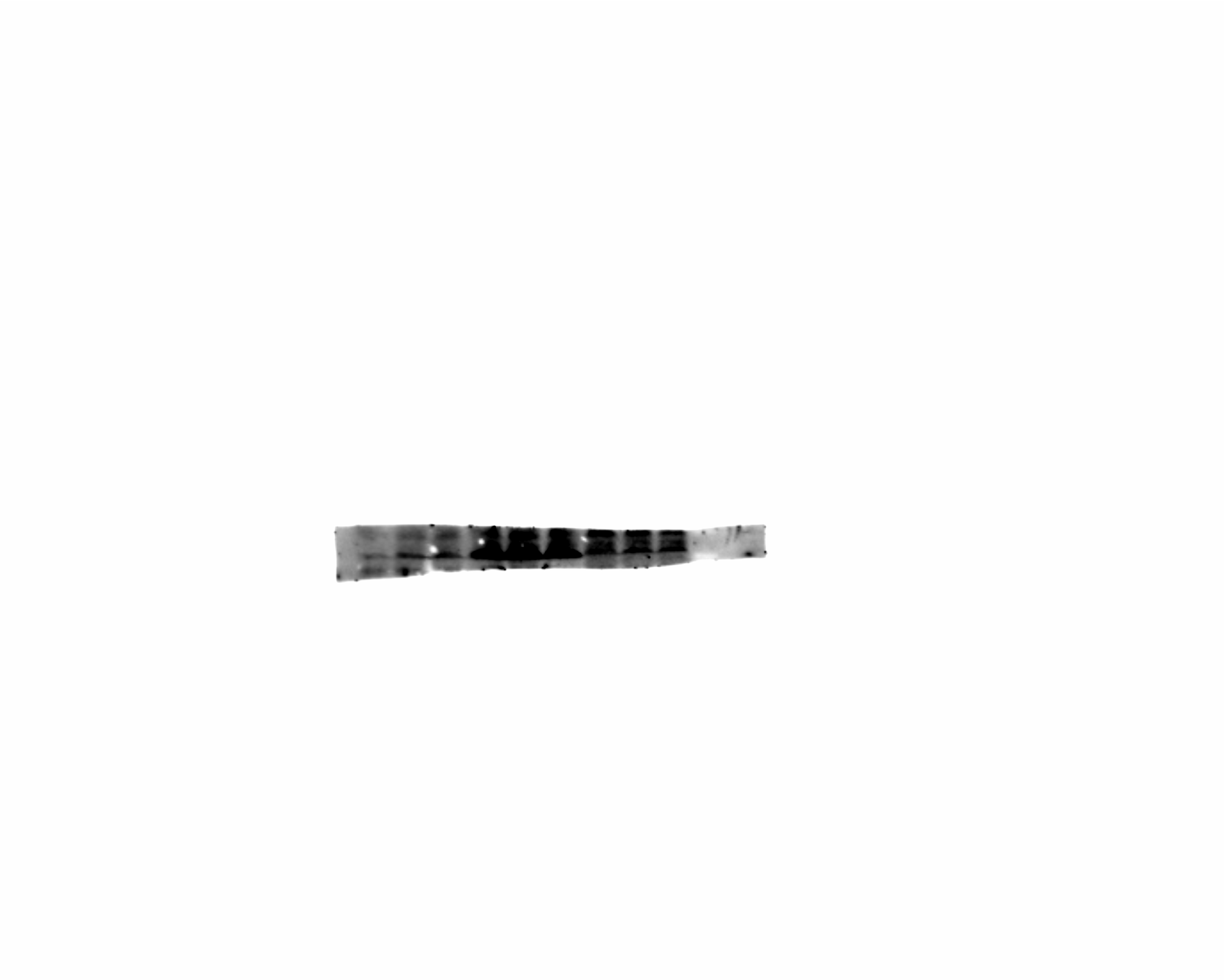

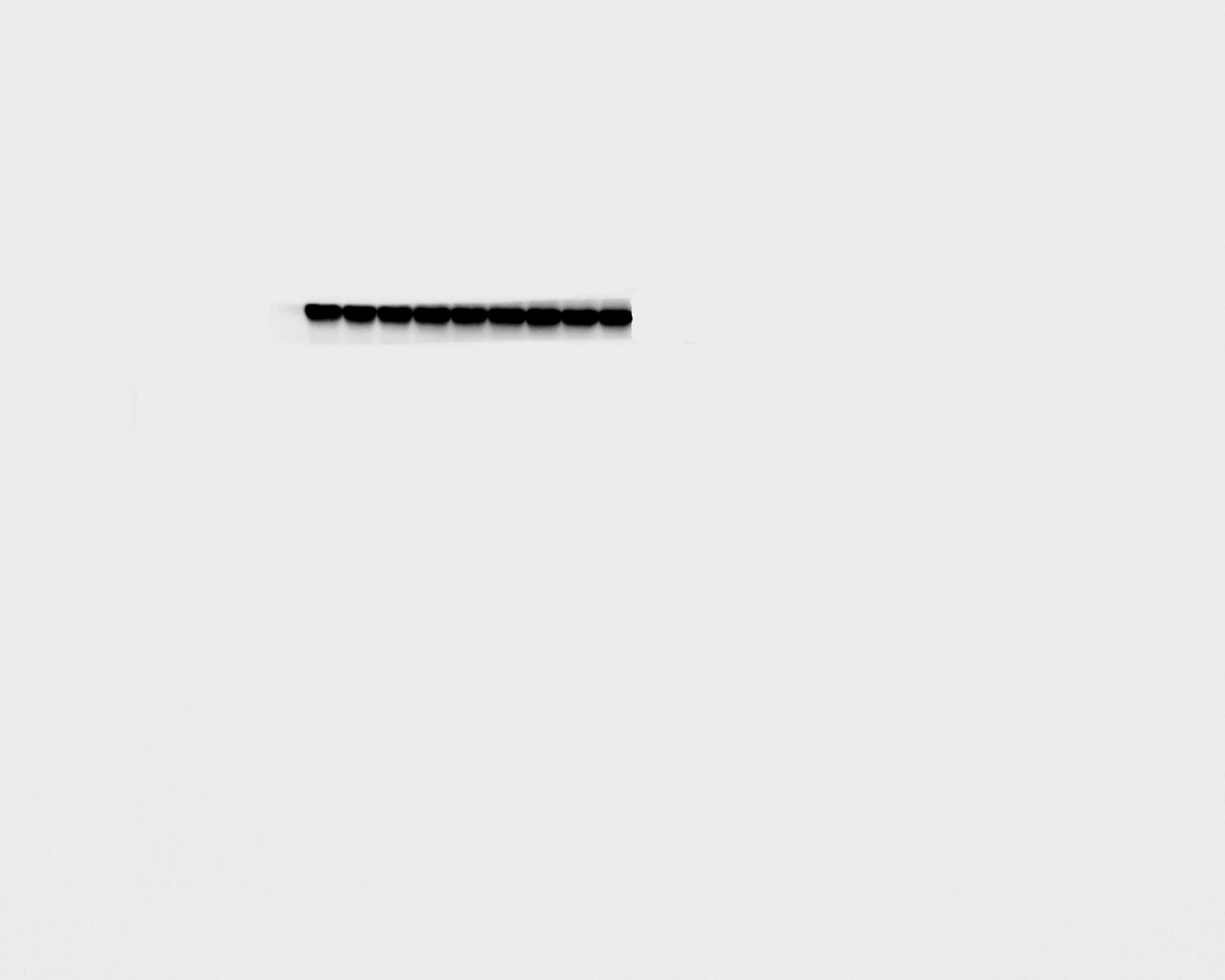


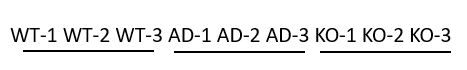

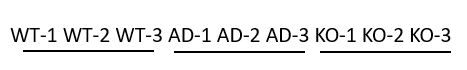


**Original blots of COX-1, EP-2, p-PKA, PKA, NLRP3, ASC, Casepase-1, IL-1β, β-Tubulin of BV2 cells treated with SC-560/TG4-155/H89/QNZ/siNF-κB(p50 or p65) (Corresponding to Figure 10)**

COX-1 ASC


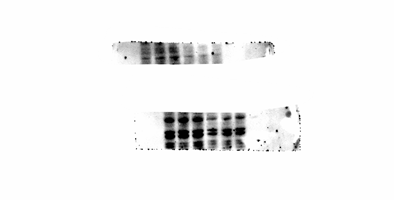


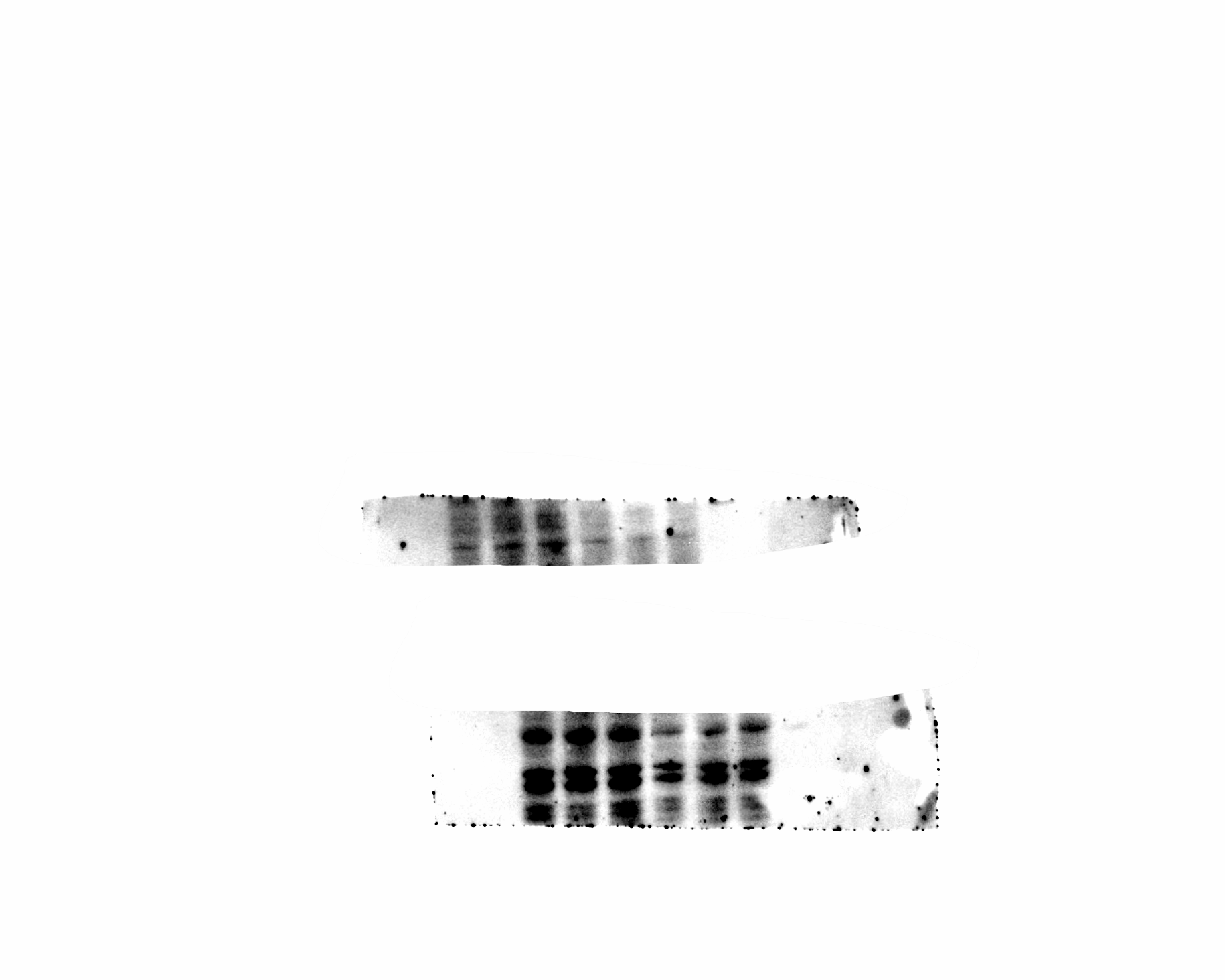


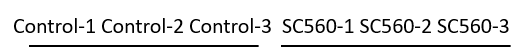

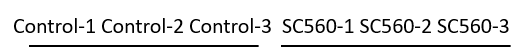


IL-1β Caspase-1


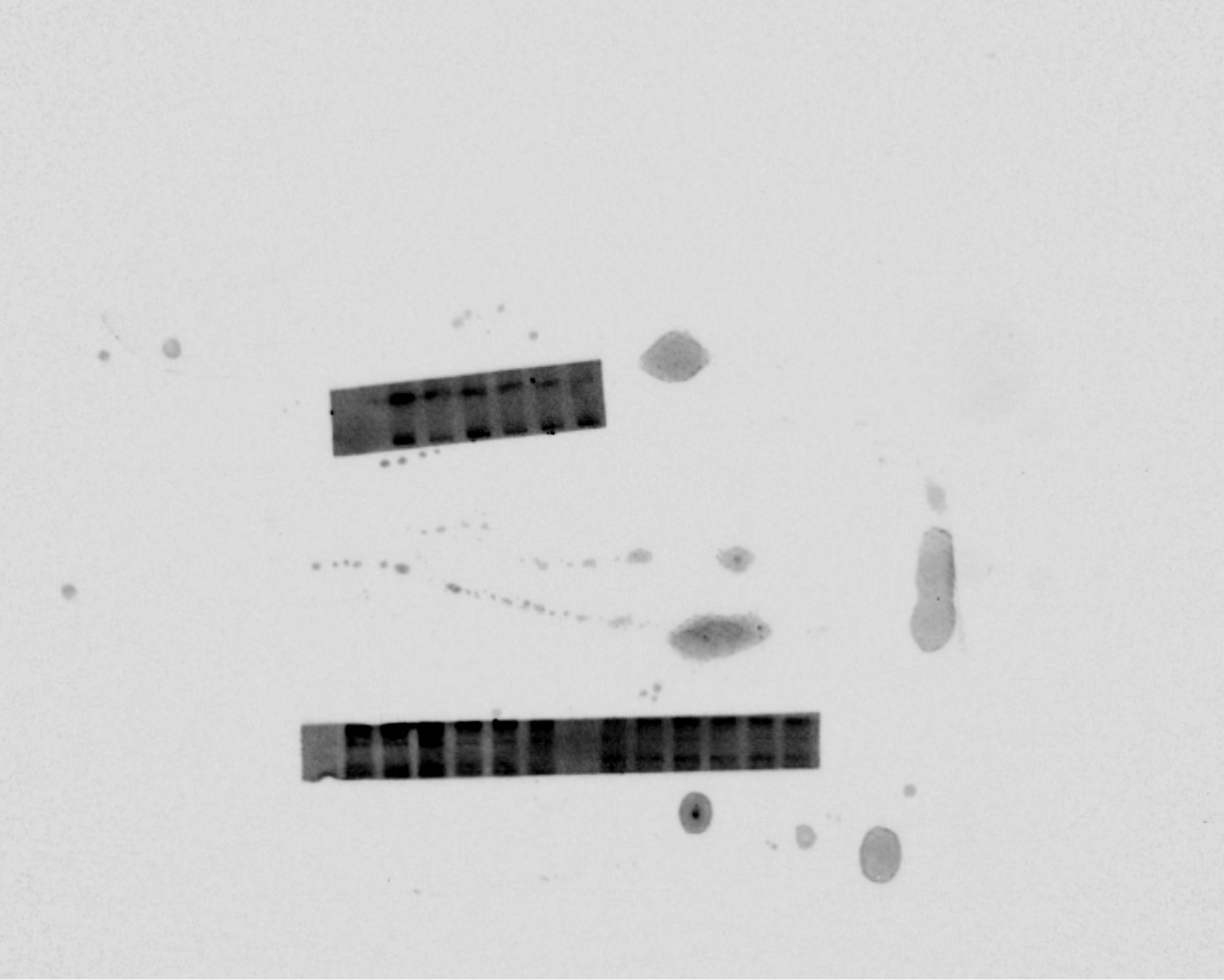


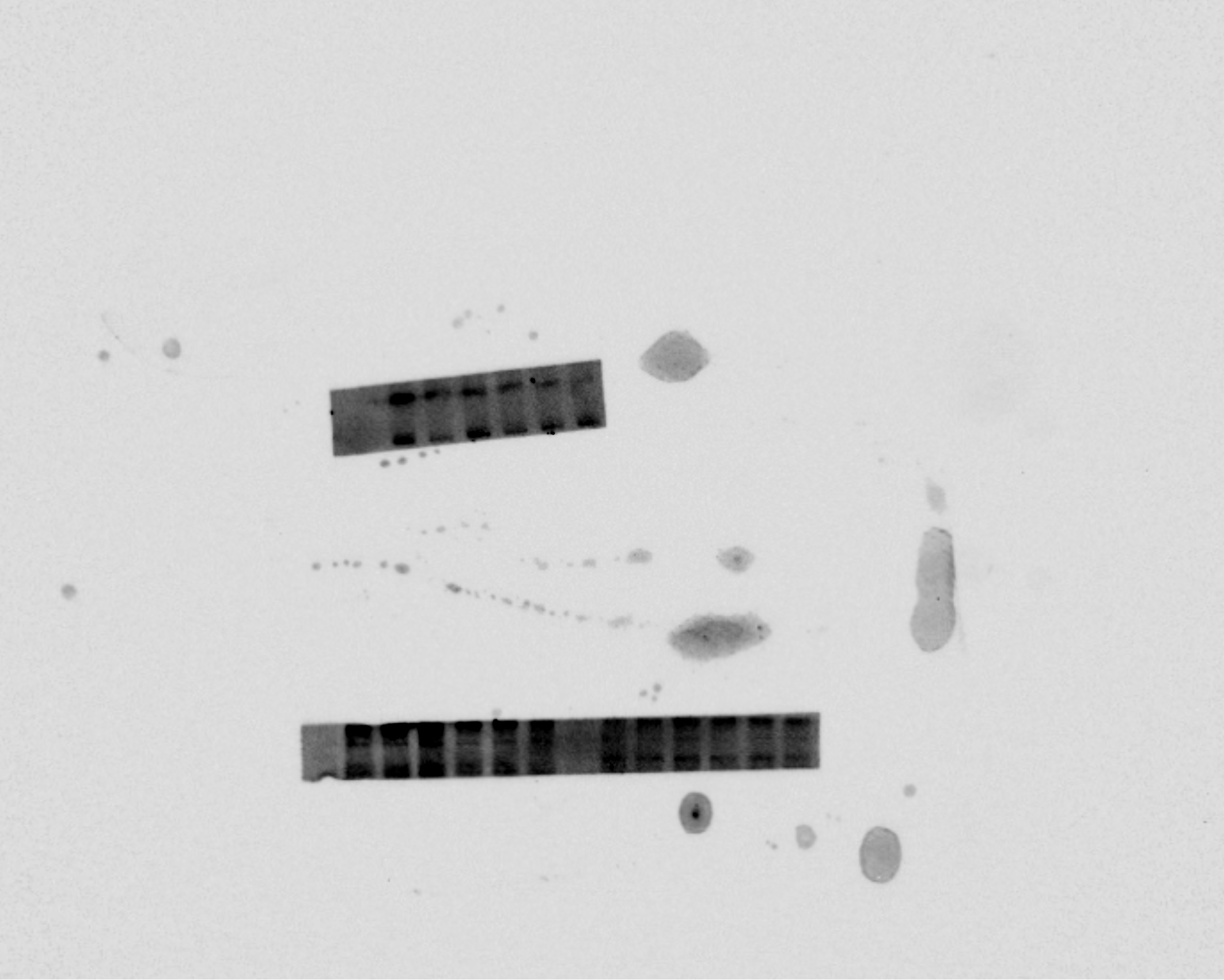


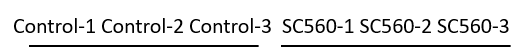

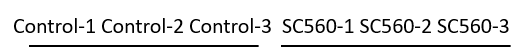


EP-2 NLRP3


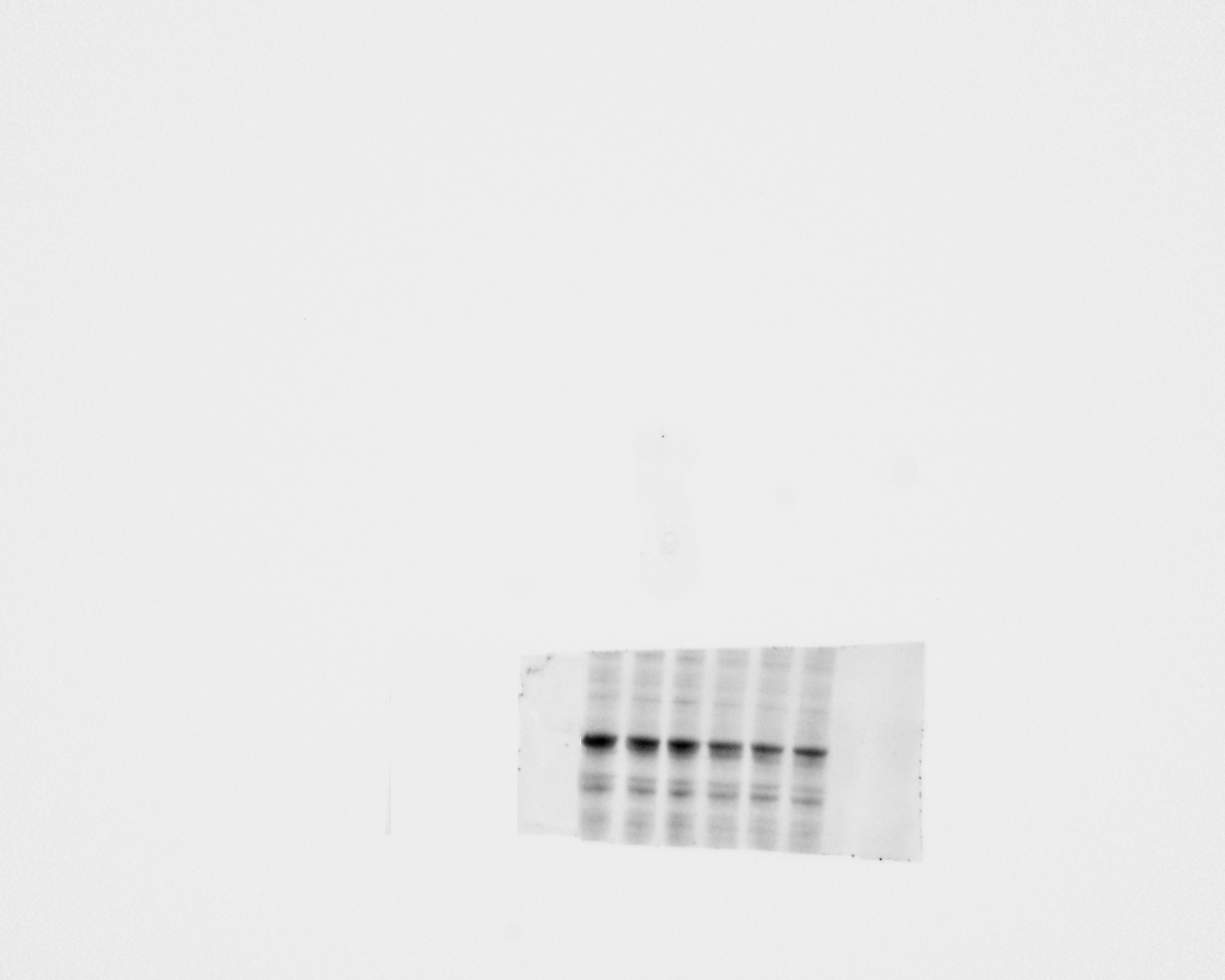

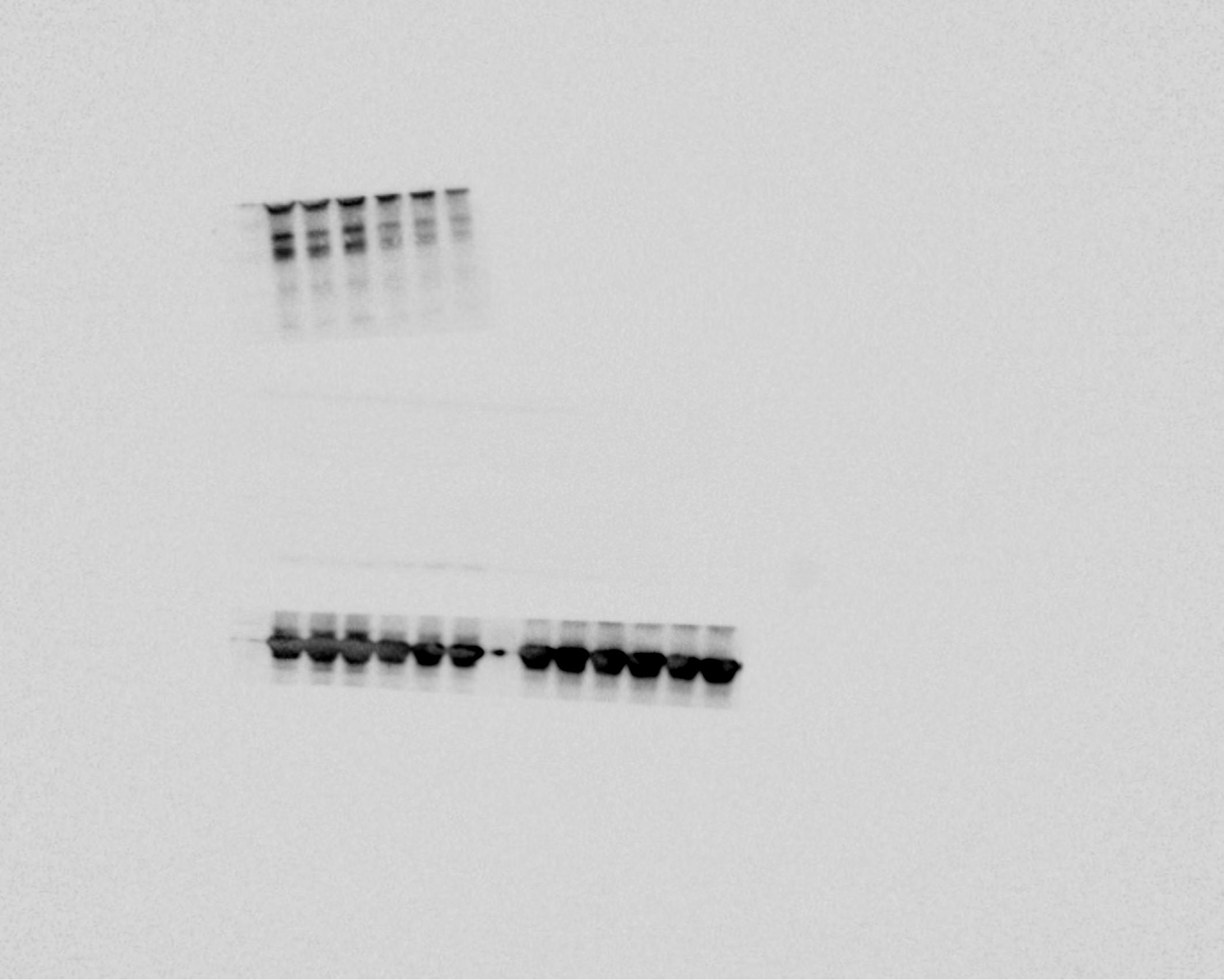


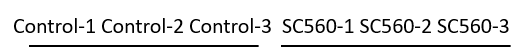

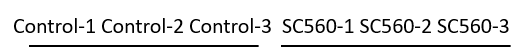


p-pka β-Tubulin


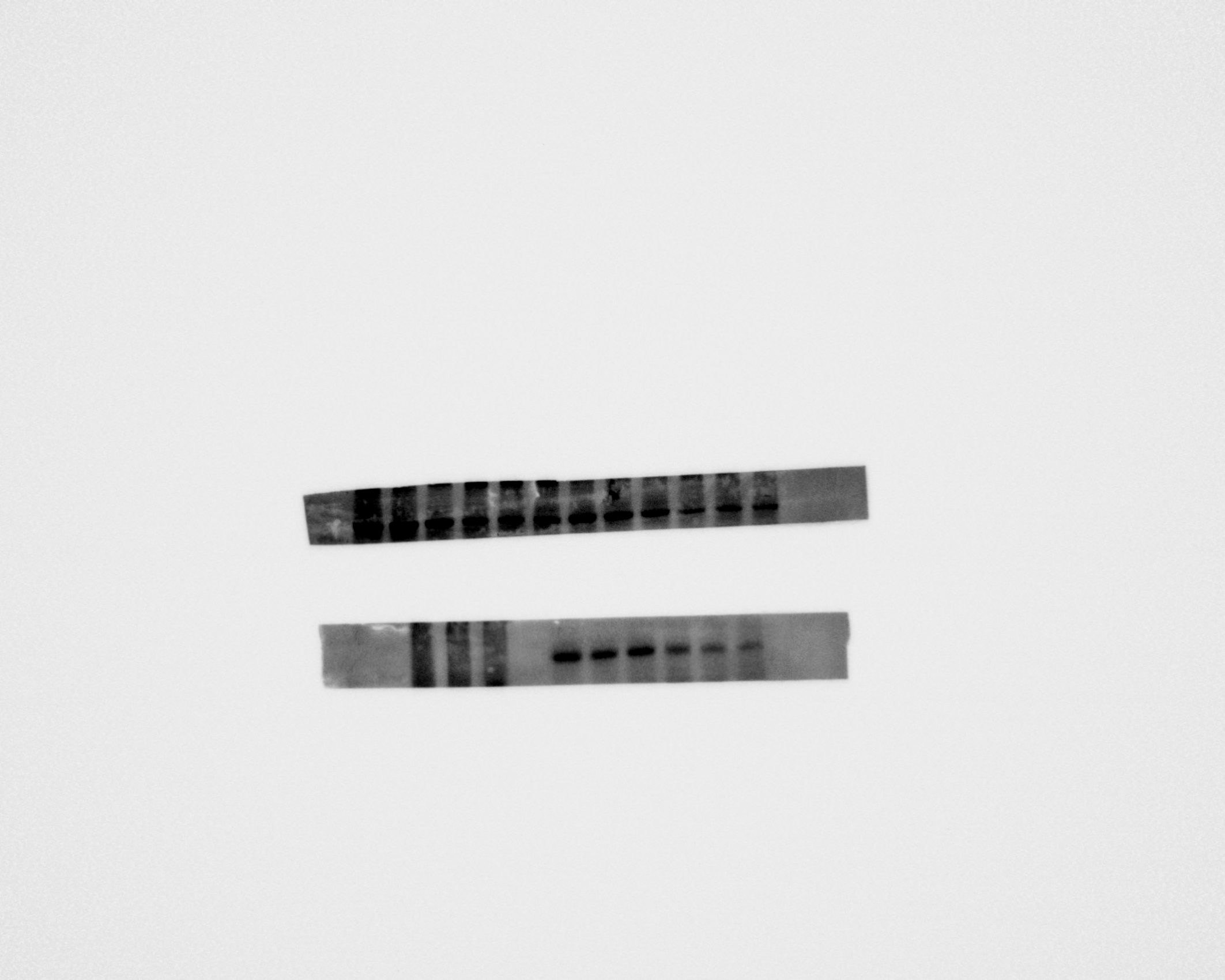

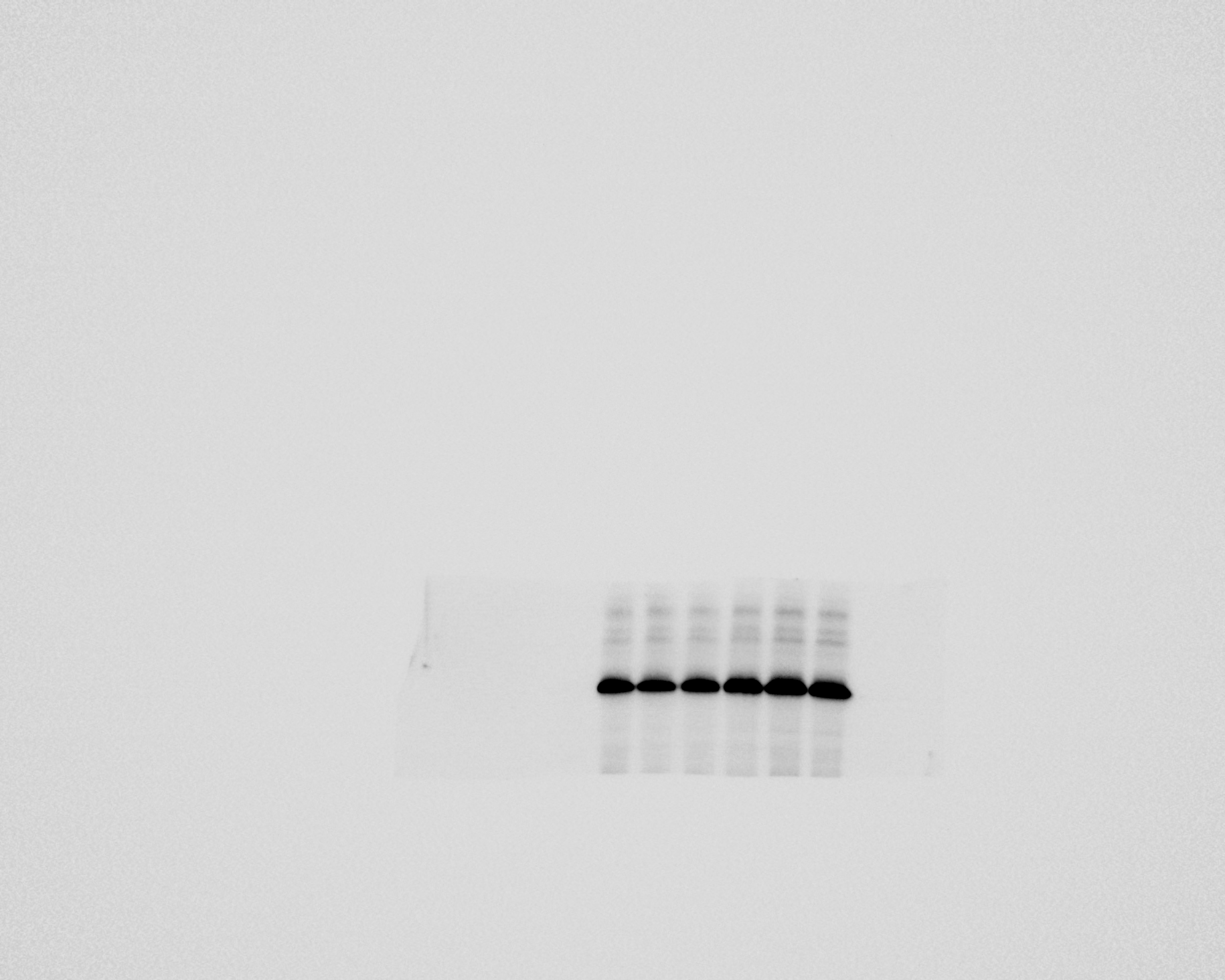


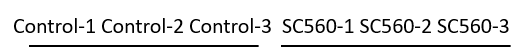

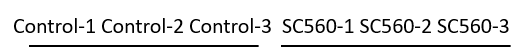


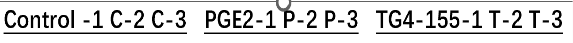
EP-2 p-PKA


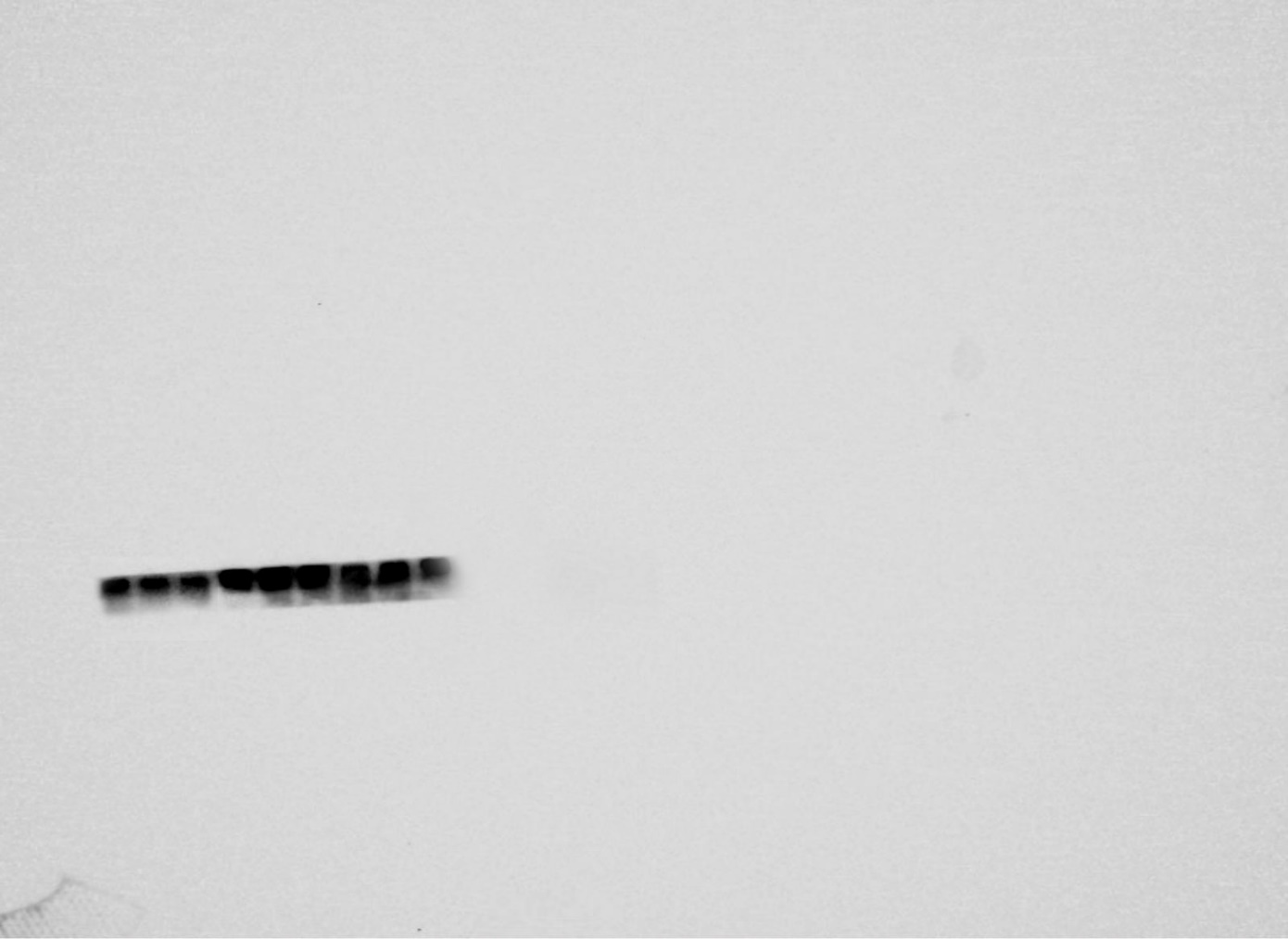

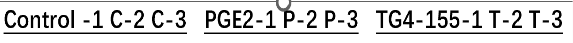

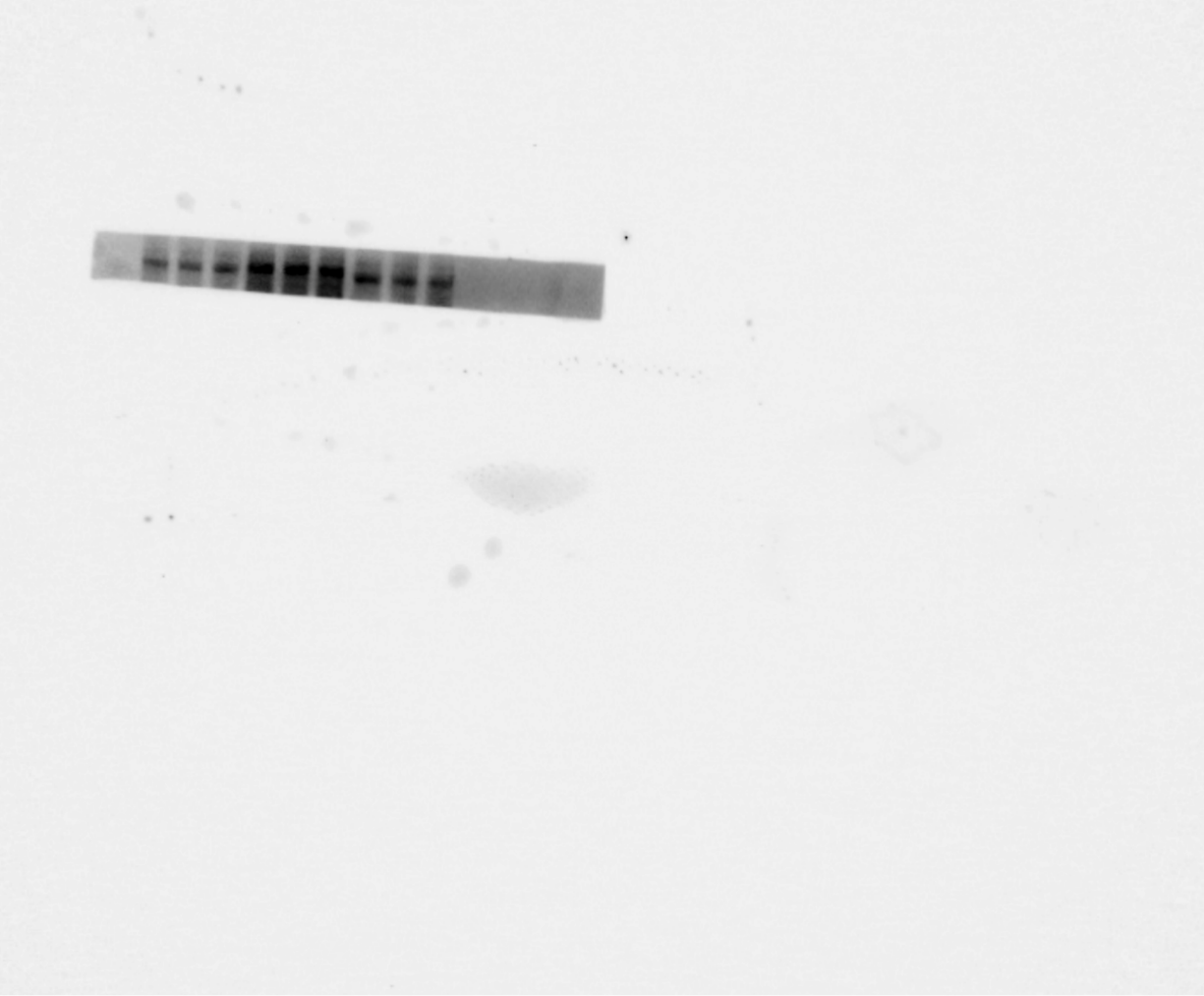


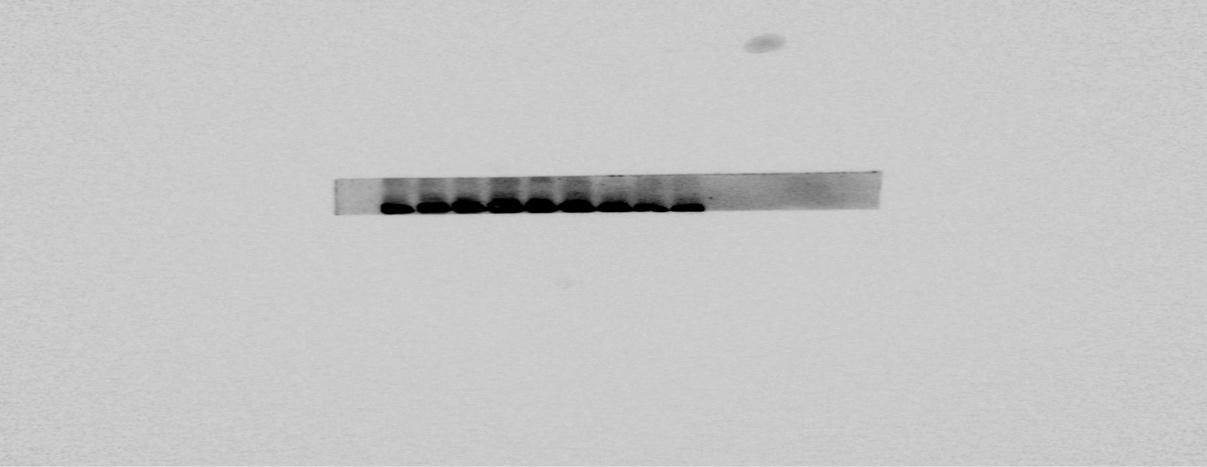

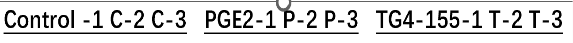

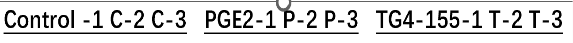
PKA NLRP3


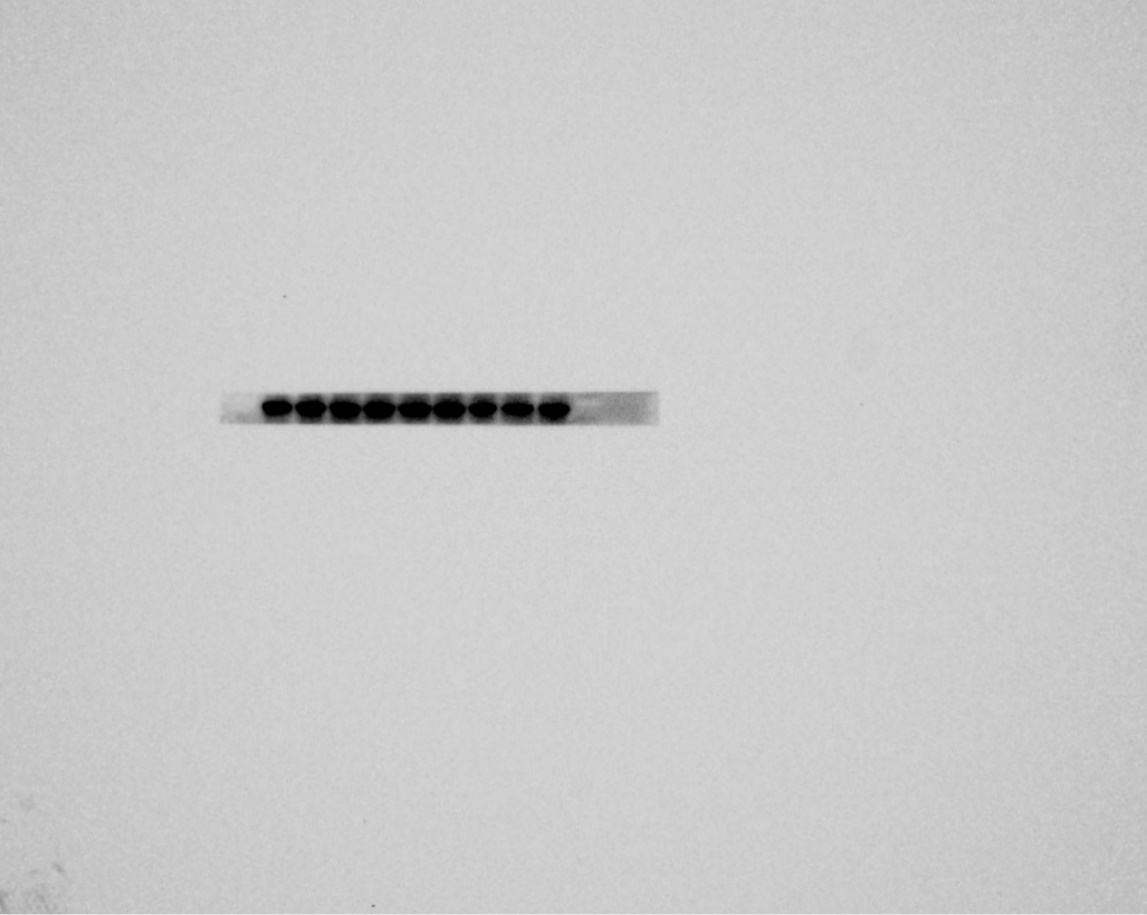


NF-kb p65 ASC


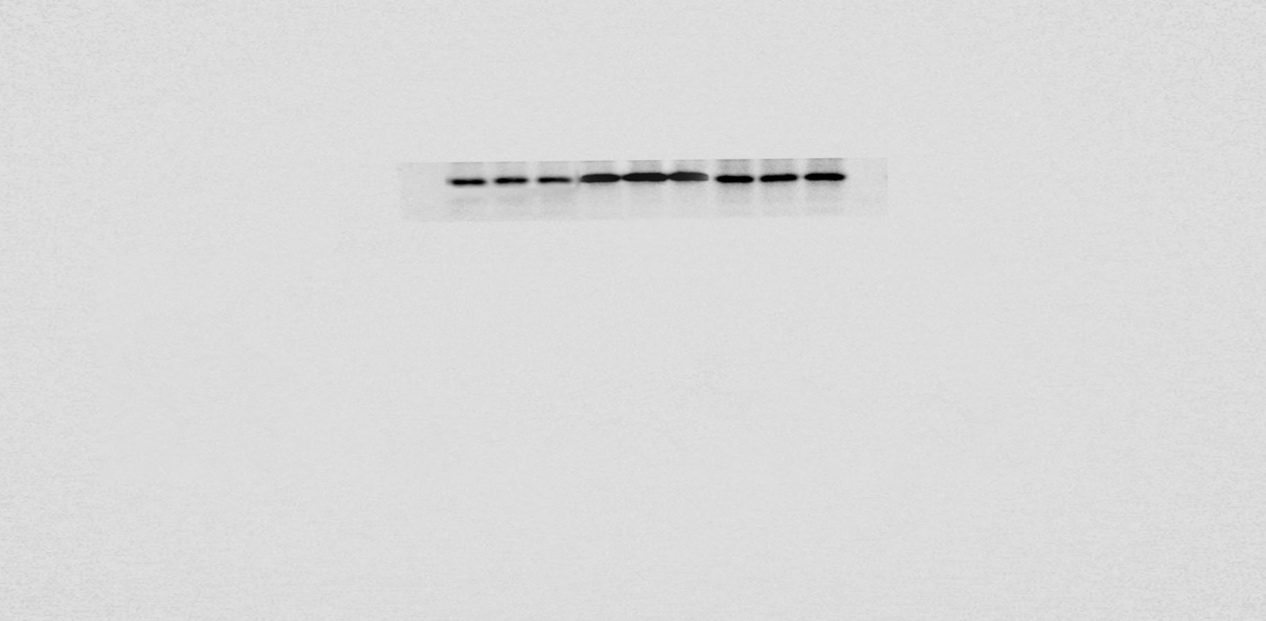

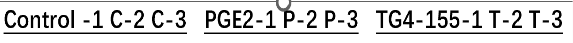

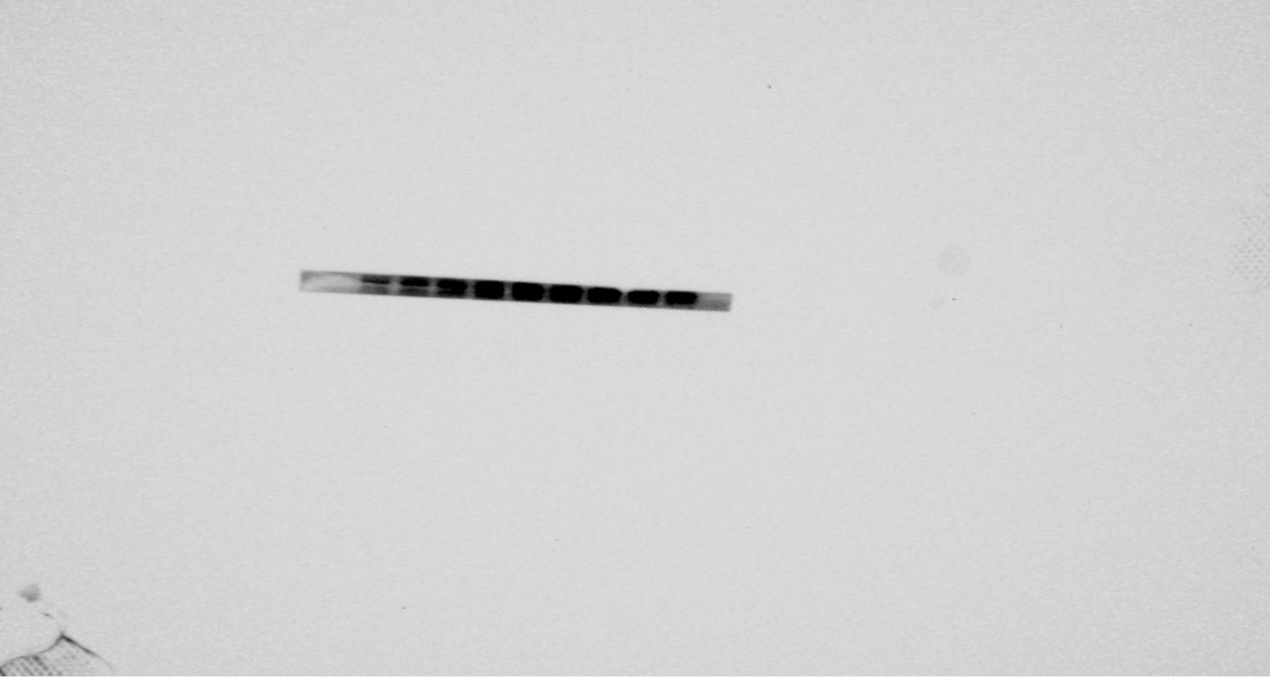


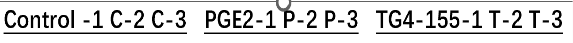


Caspase-1 IL-1β


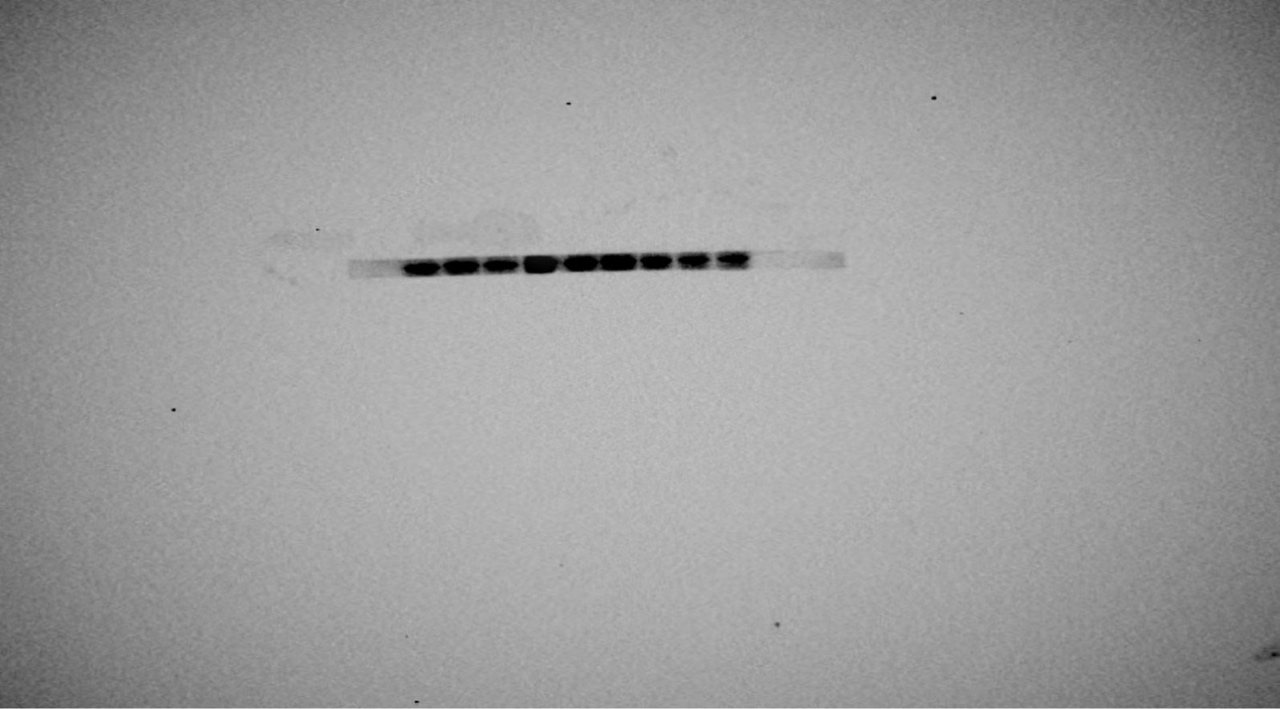

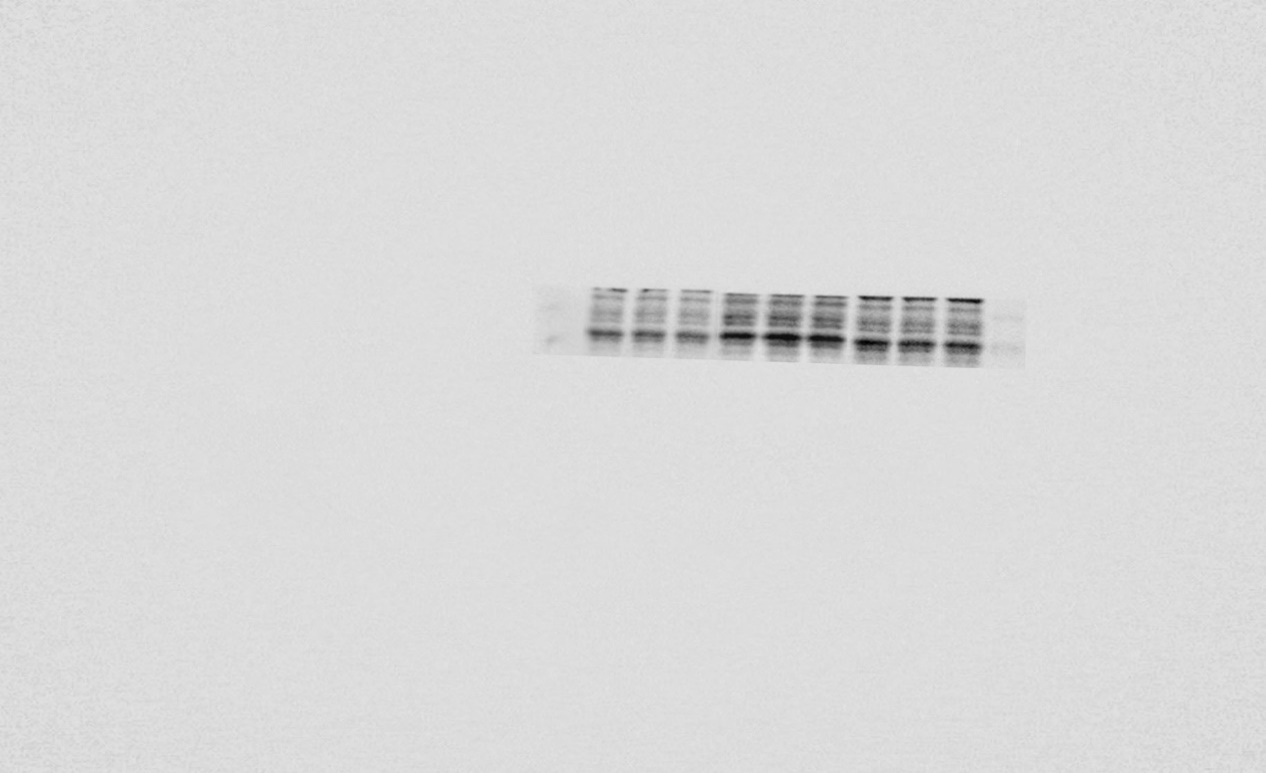


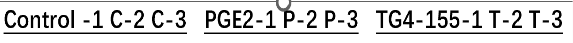

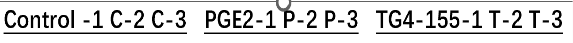


Tubulin


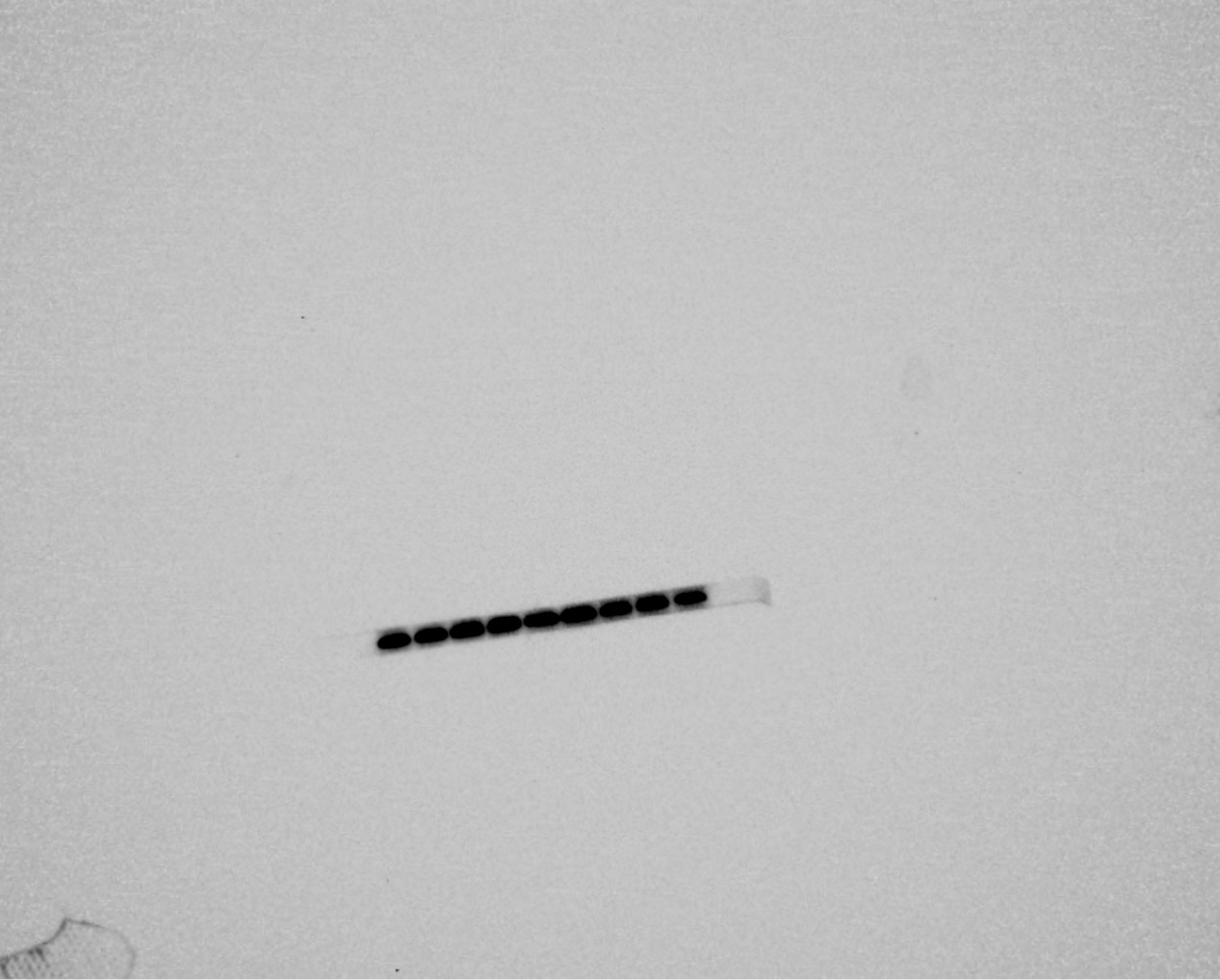


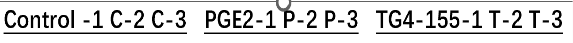


EP-2 p-PKA


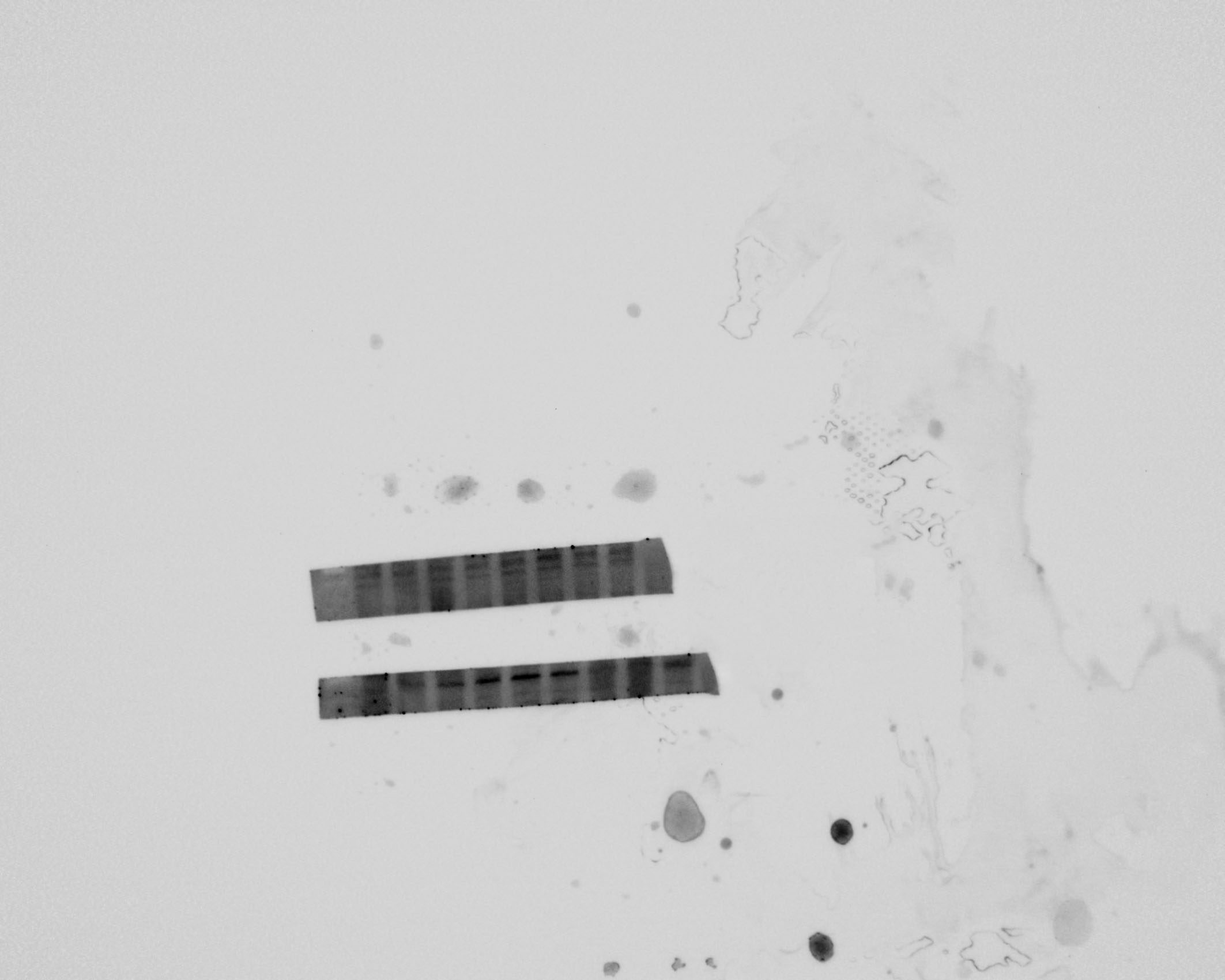

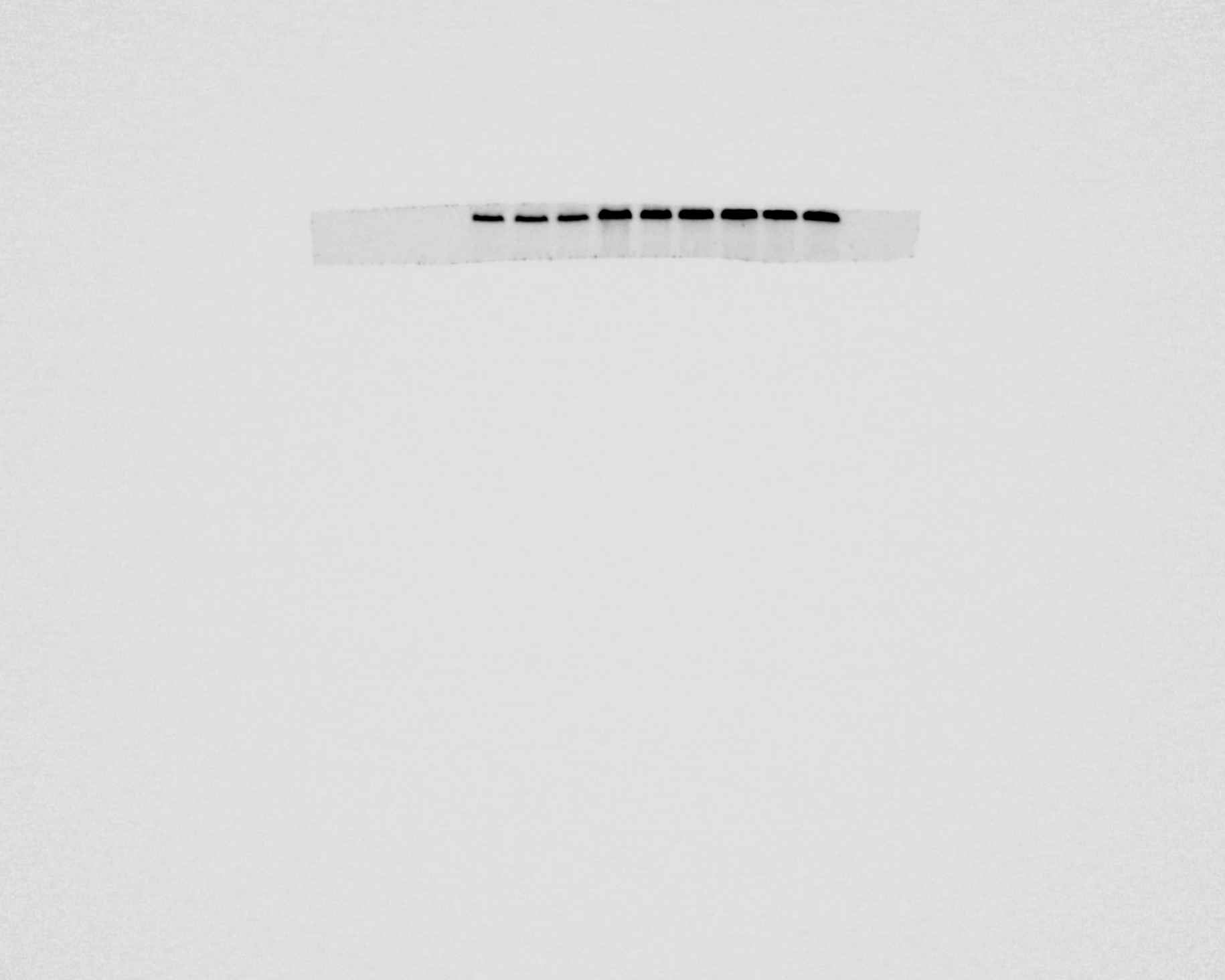


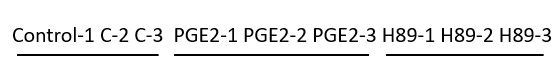


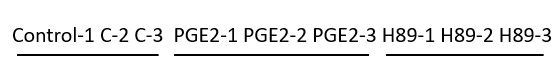


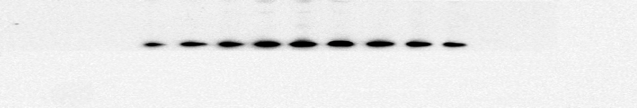

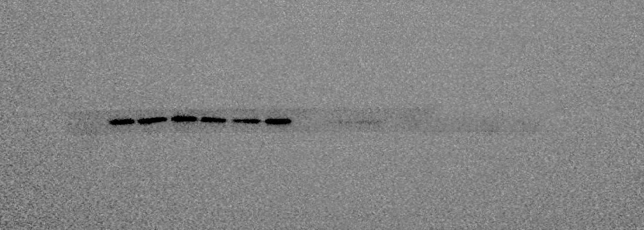
PKA NLRP3
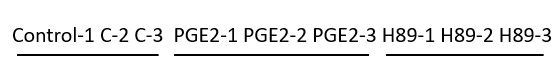


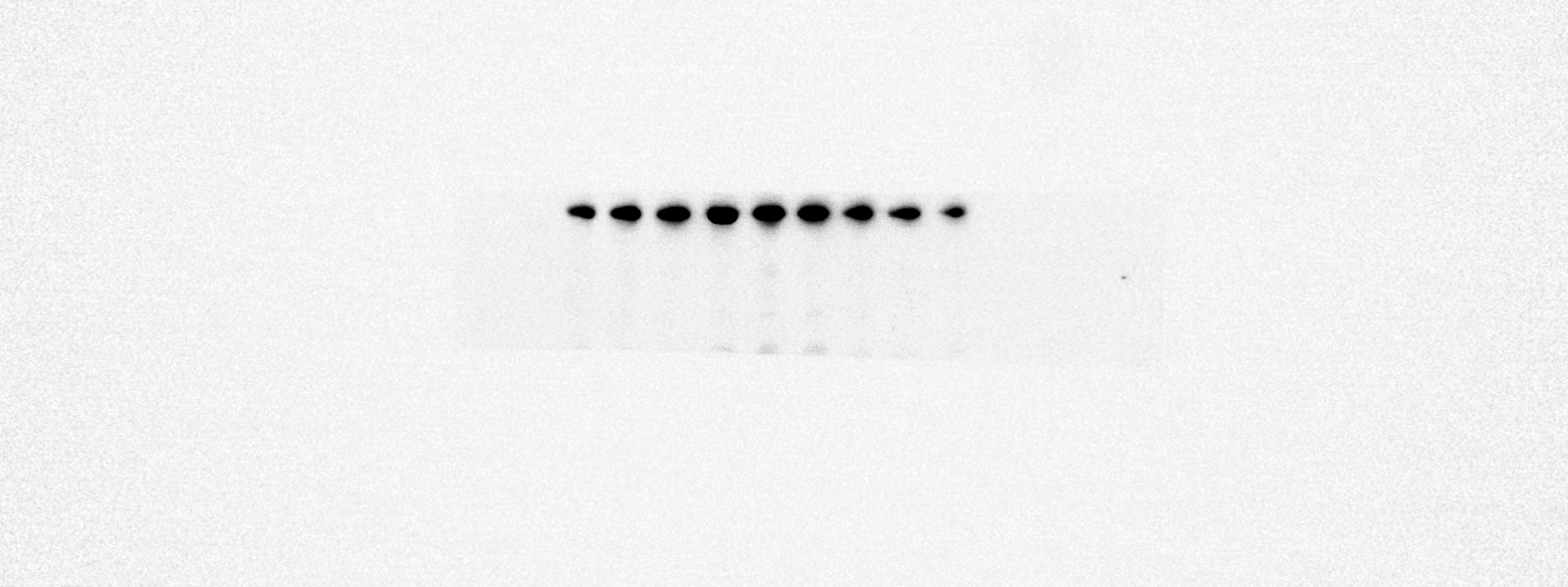


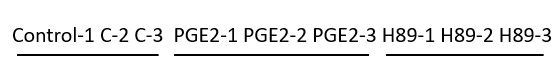


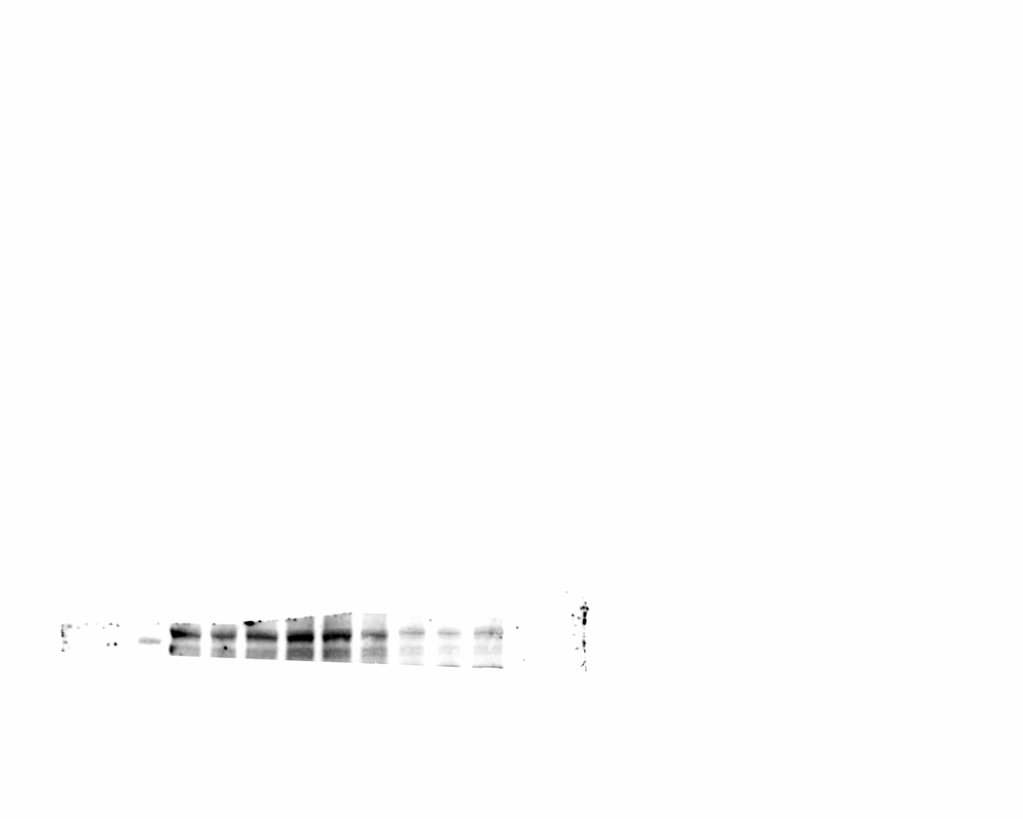
NF-Kb p65 ASC


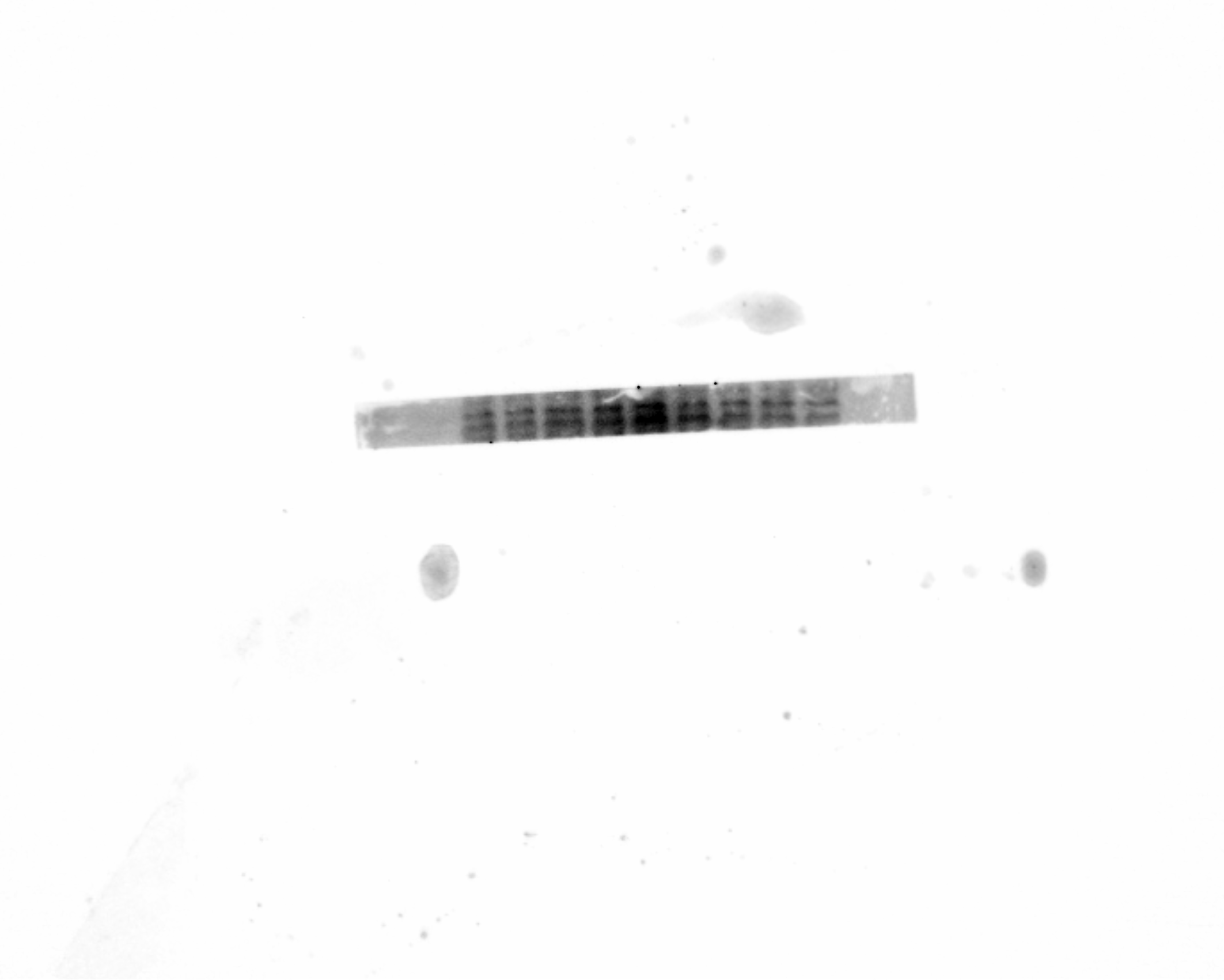


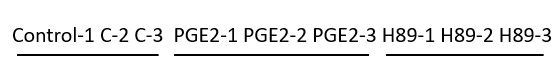

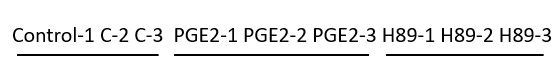


Caspase-1 IL-1β


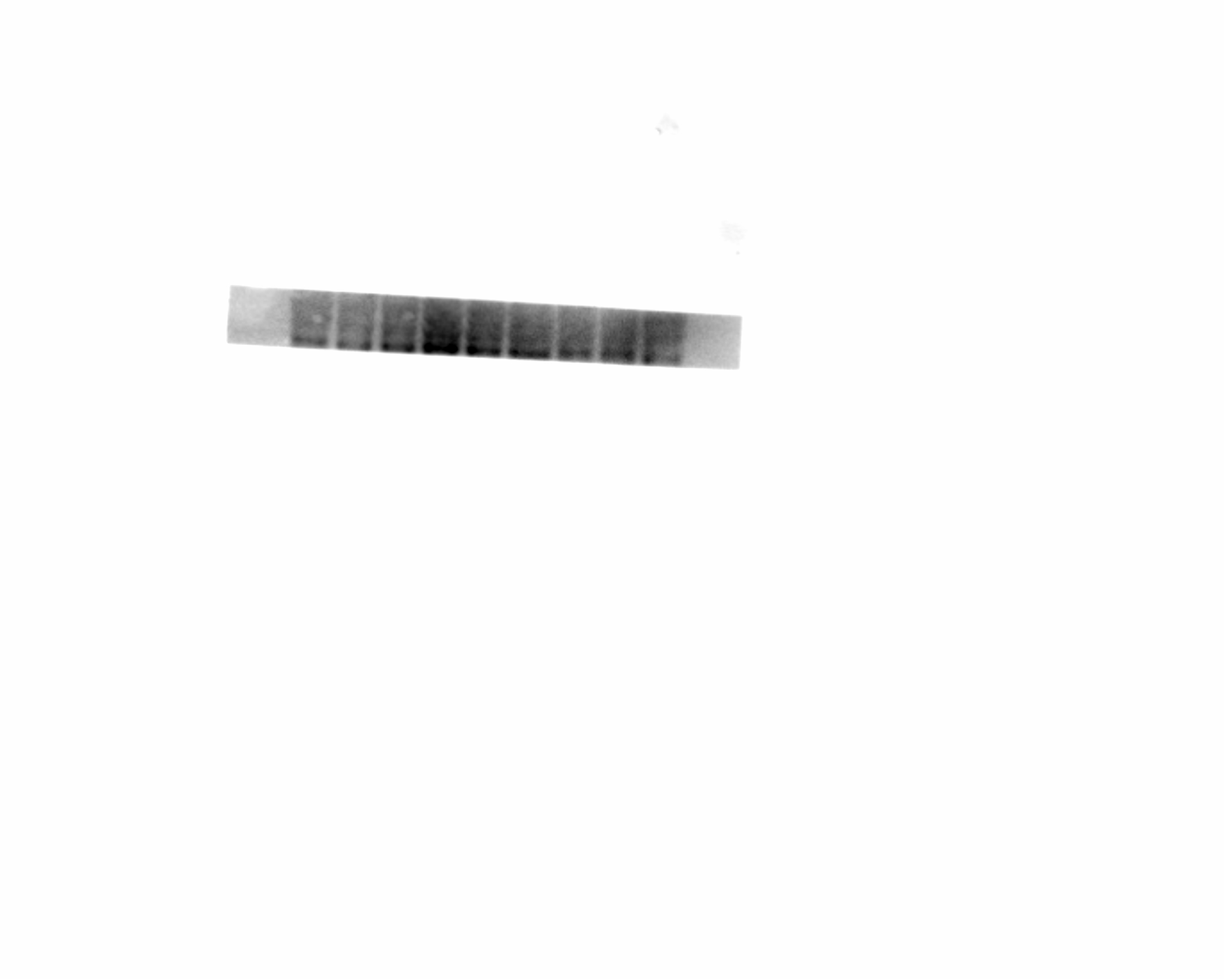

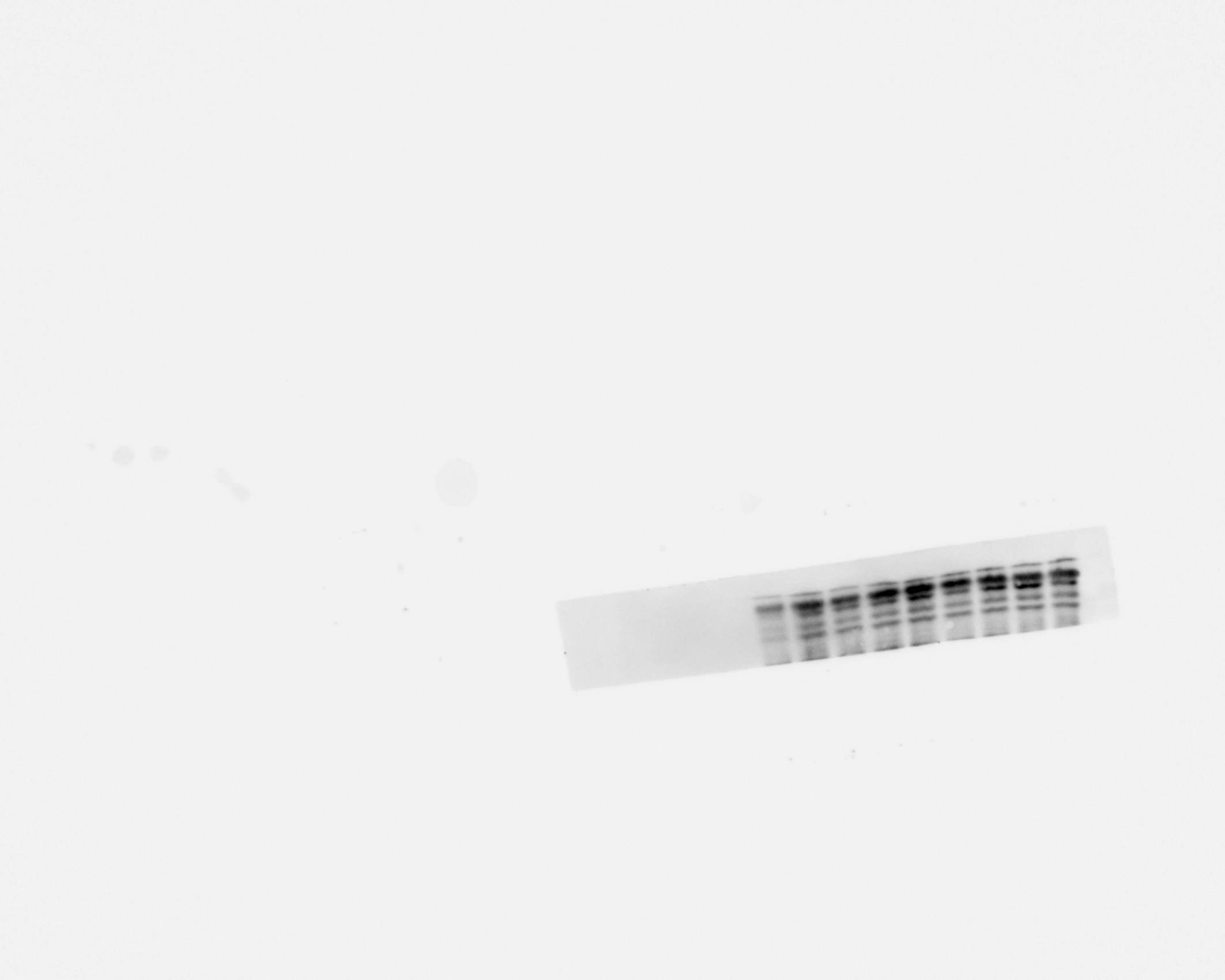


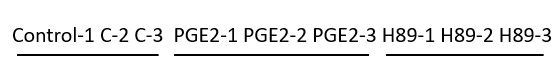


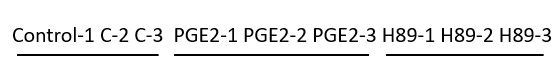


β-Tubulin


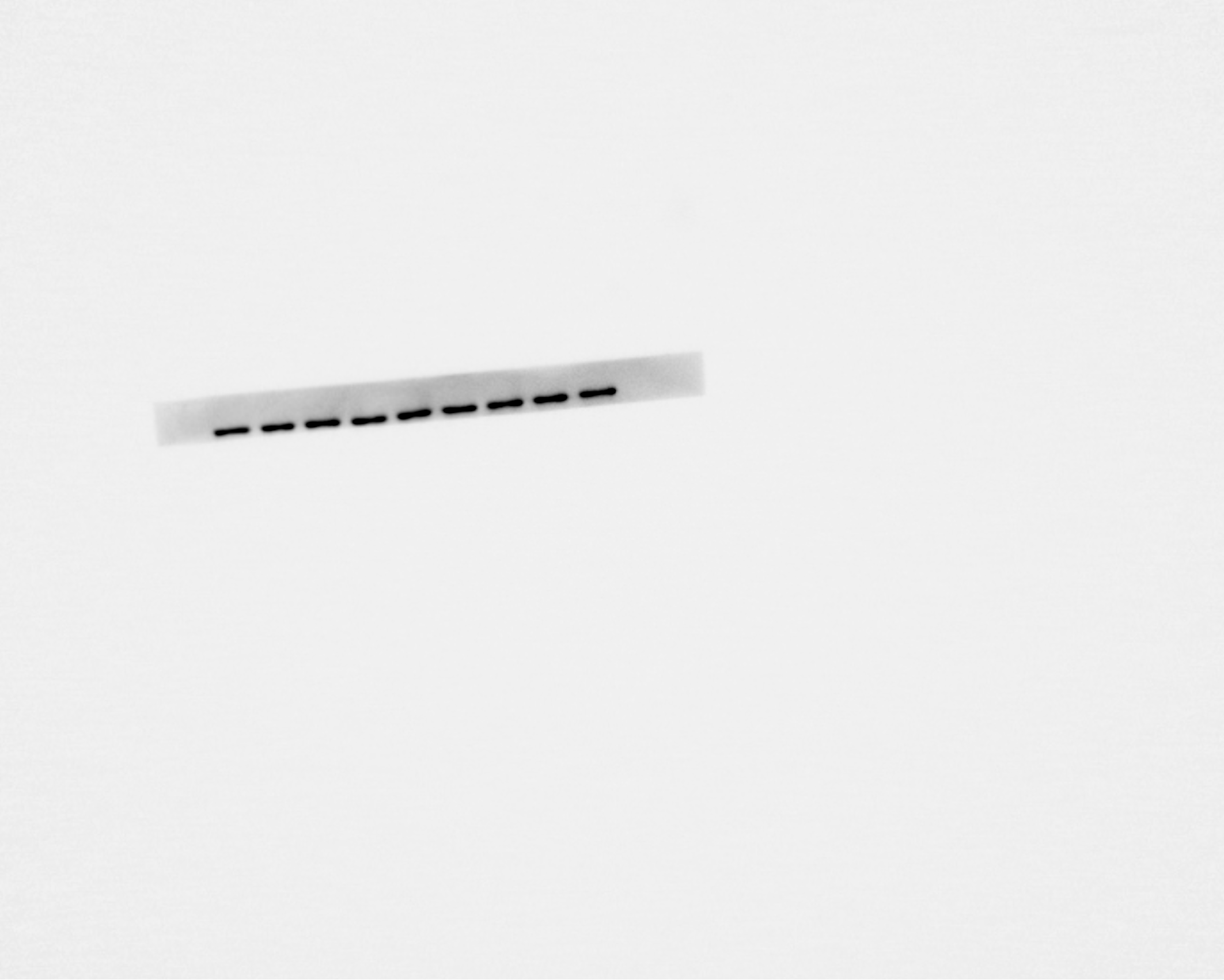


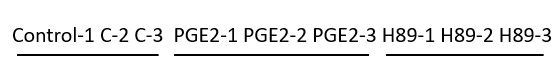


EP-2 p-PKA


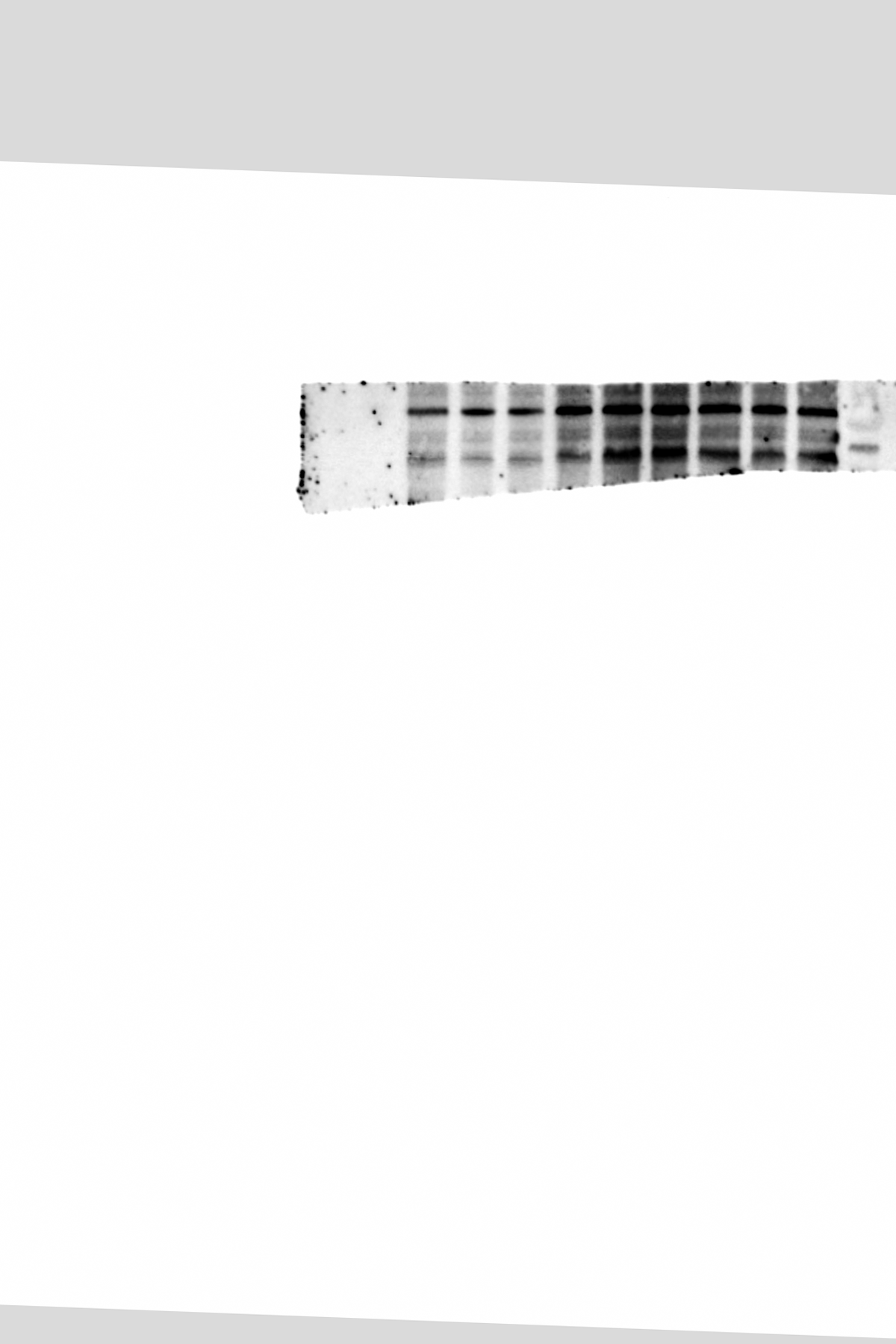

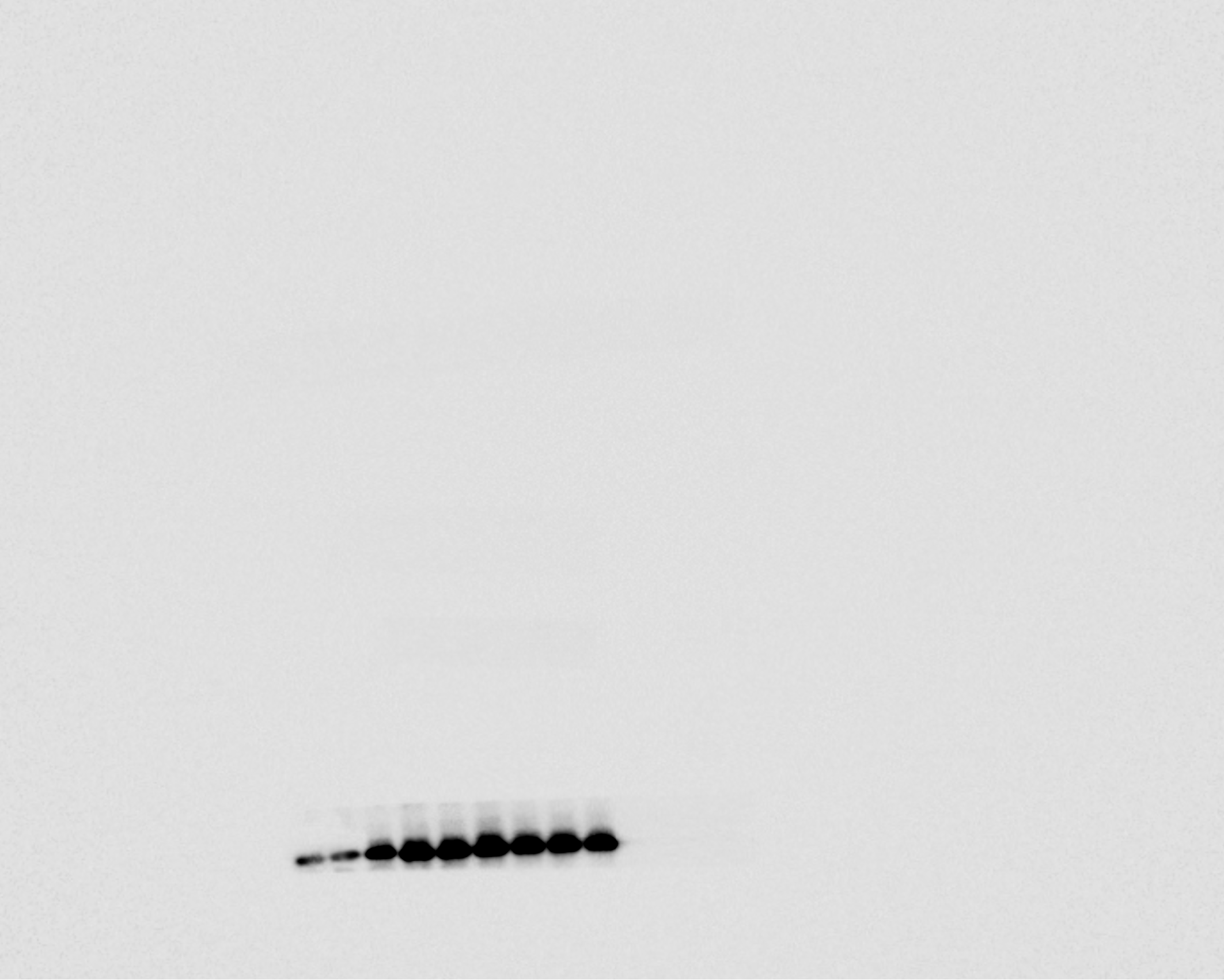


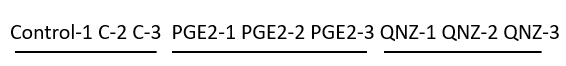

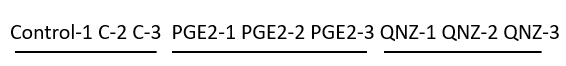


PKA NLRP3


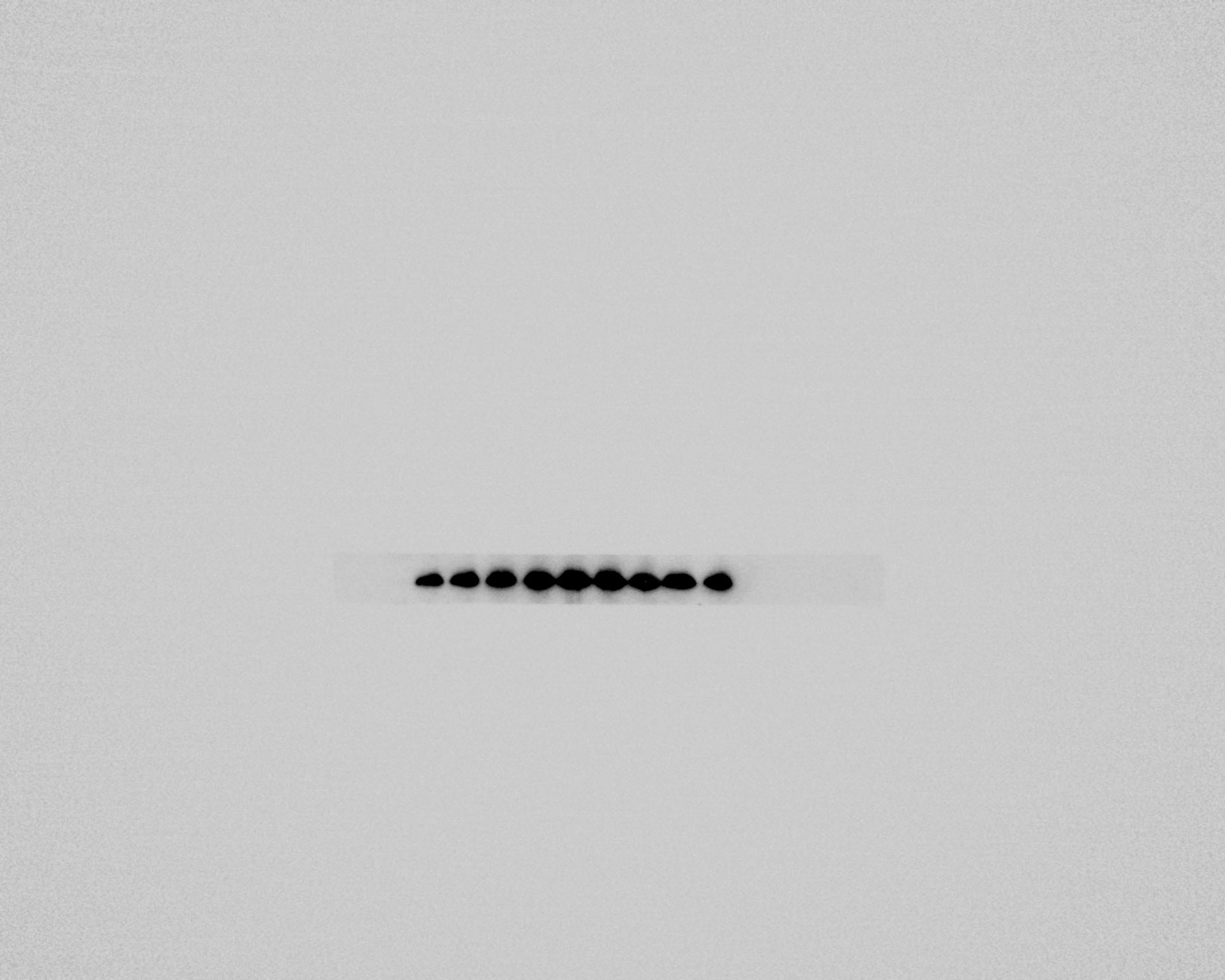

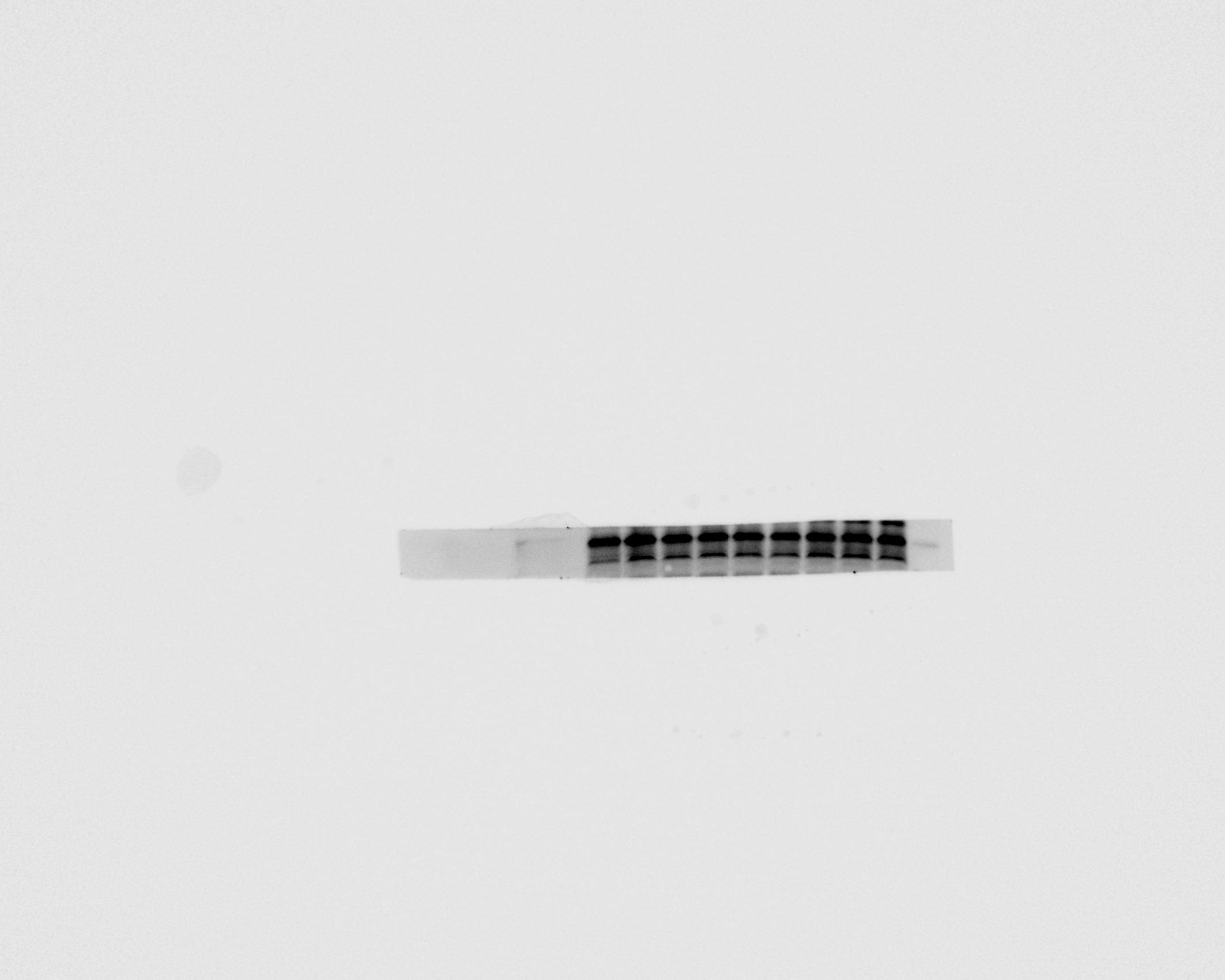


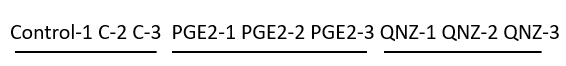

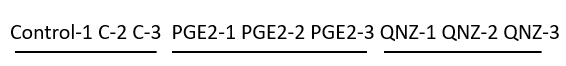


NF-Kb p65 Caspase-1


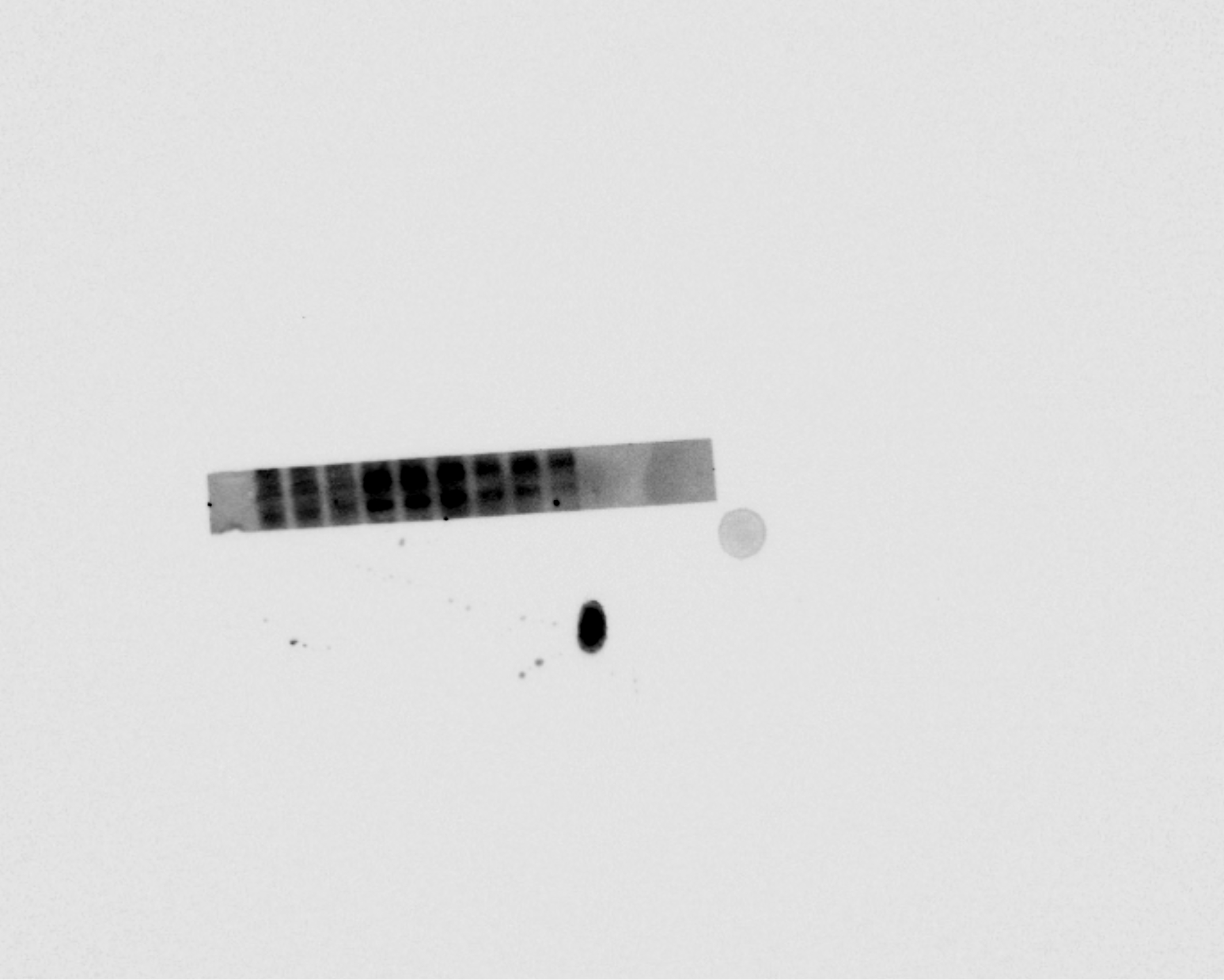

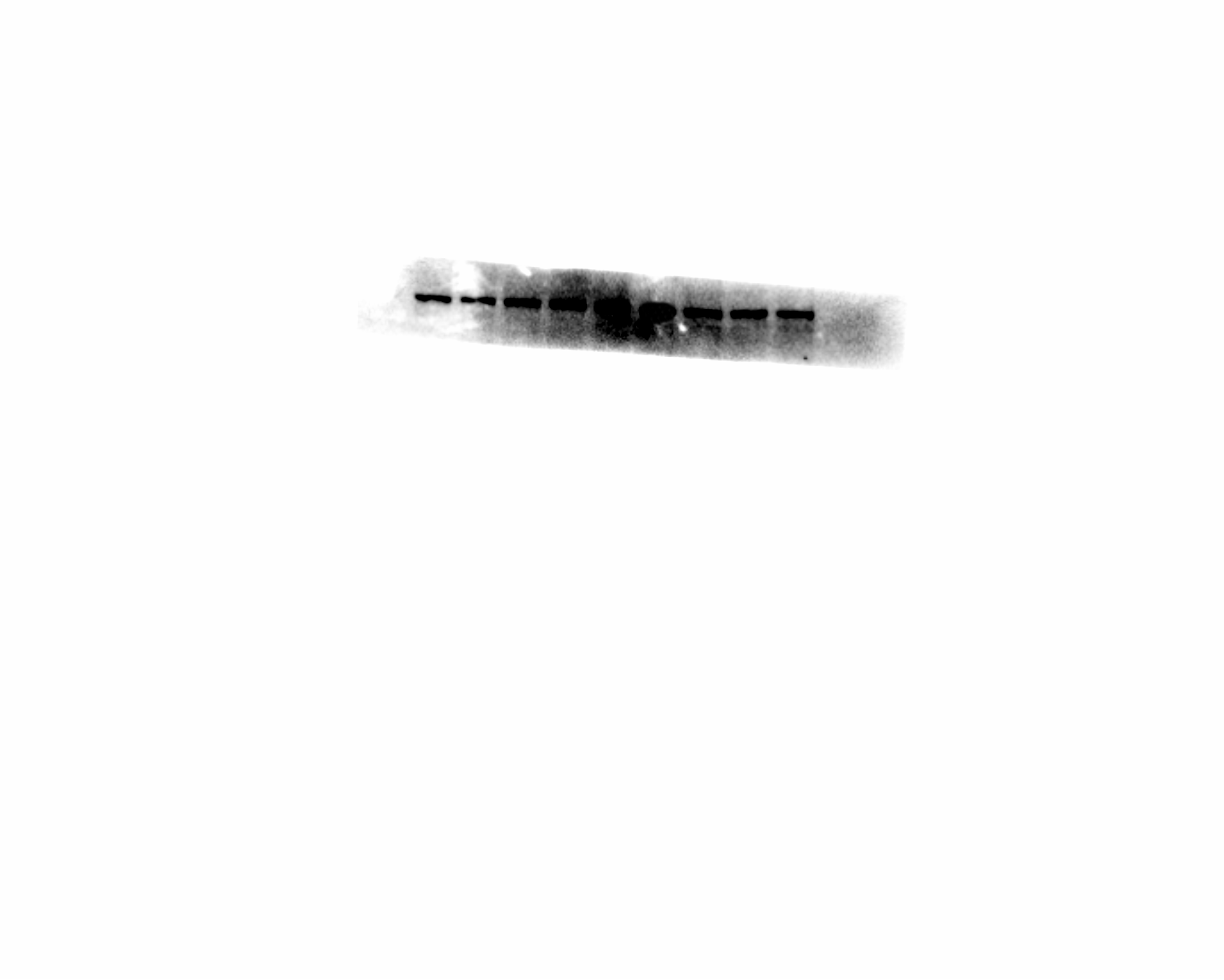


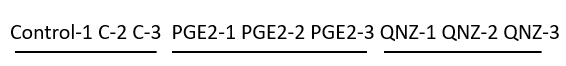

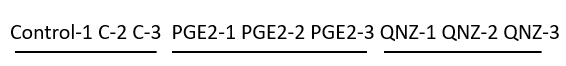


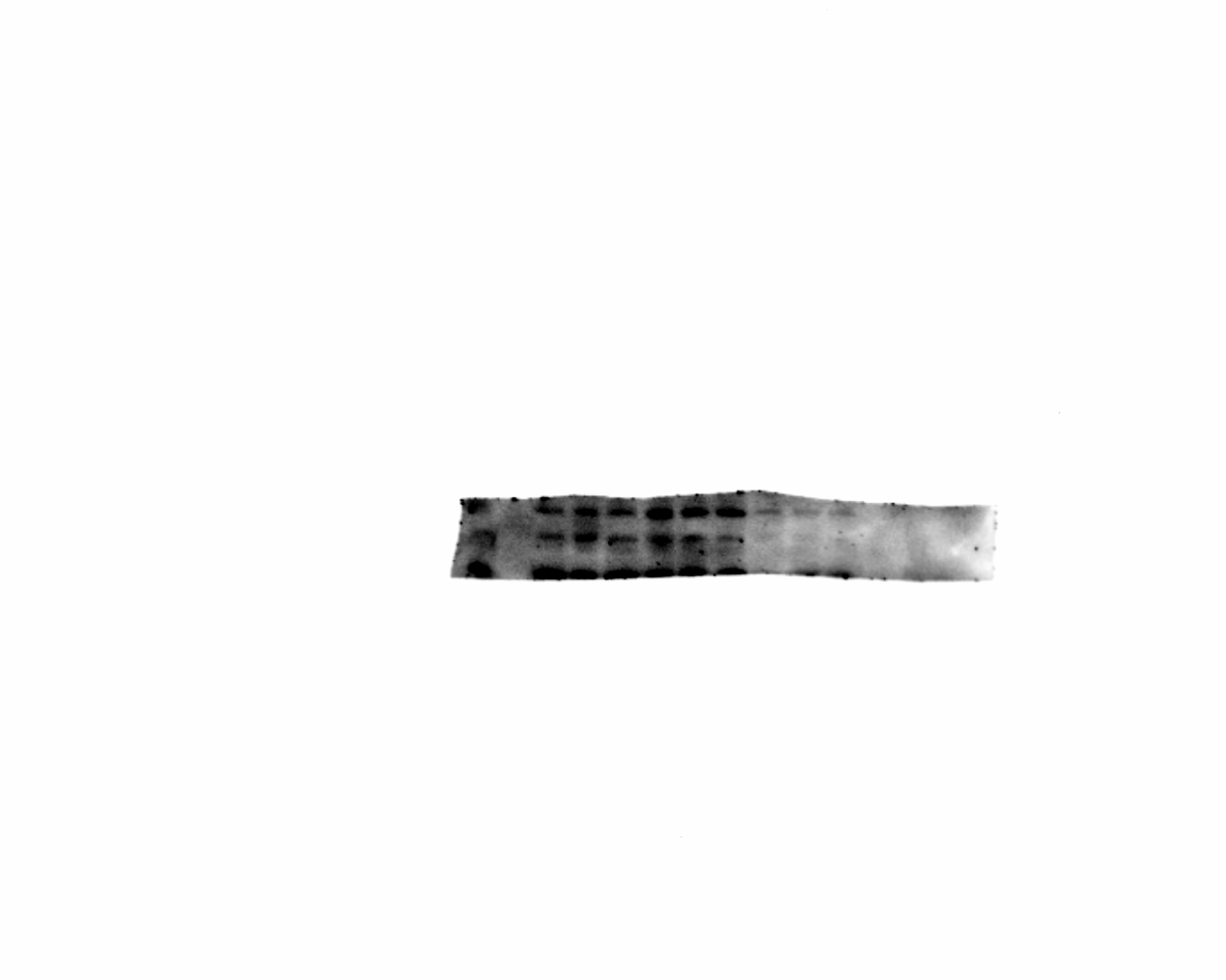


ASC β-Tubulin


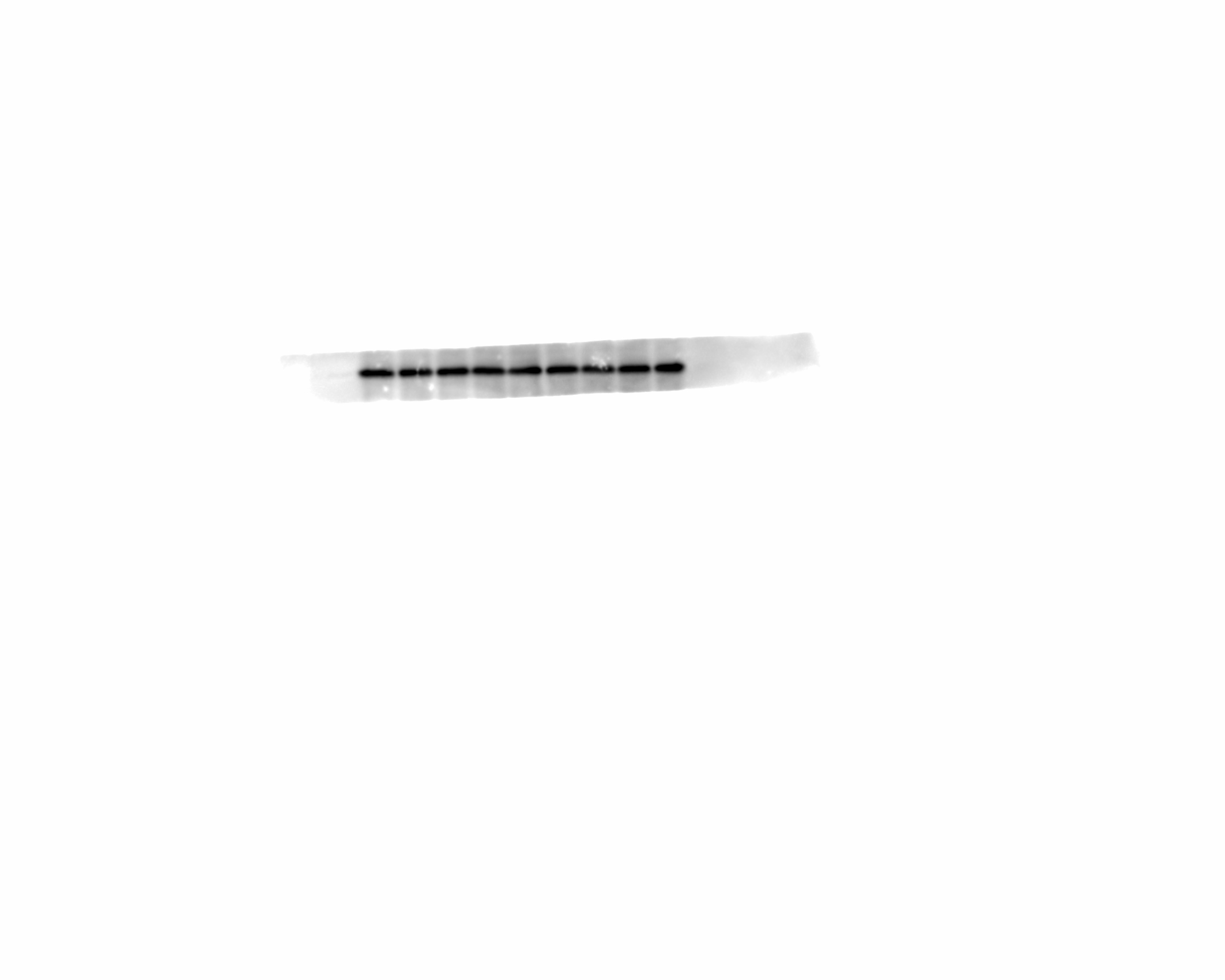


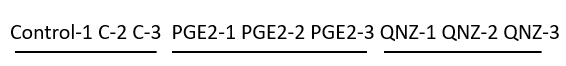

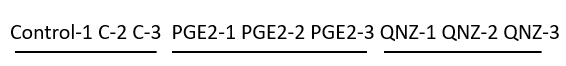


EP-2 p-PKA


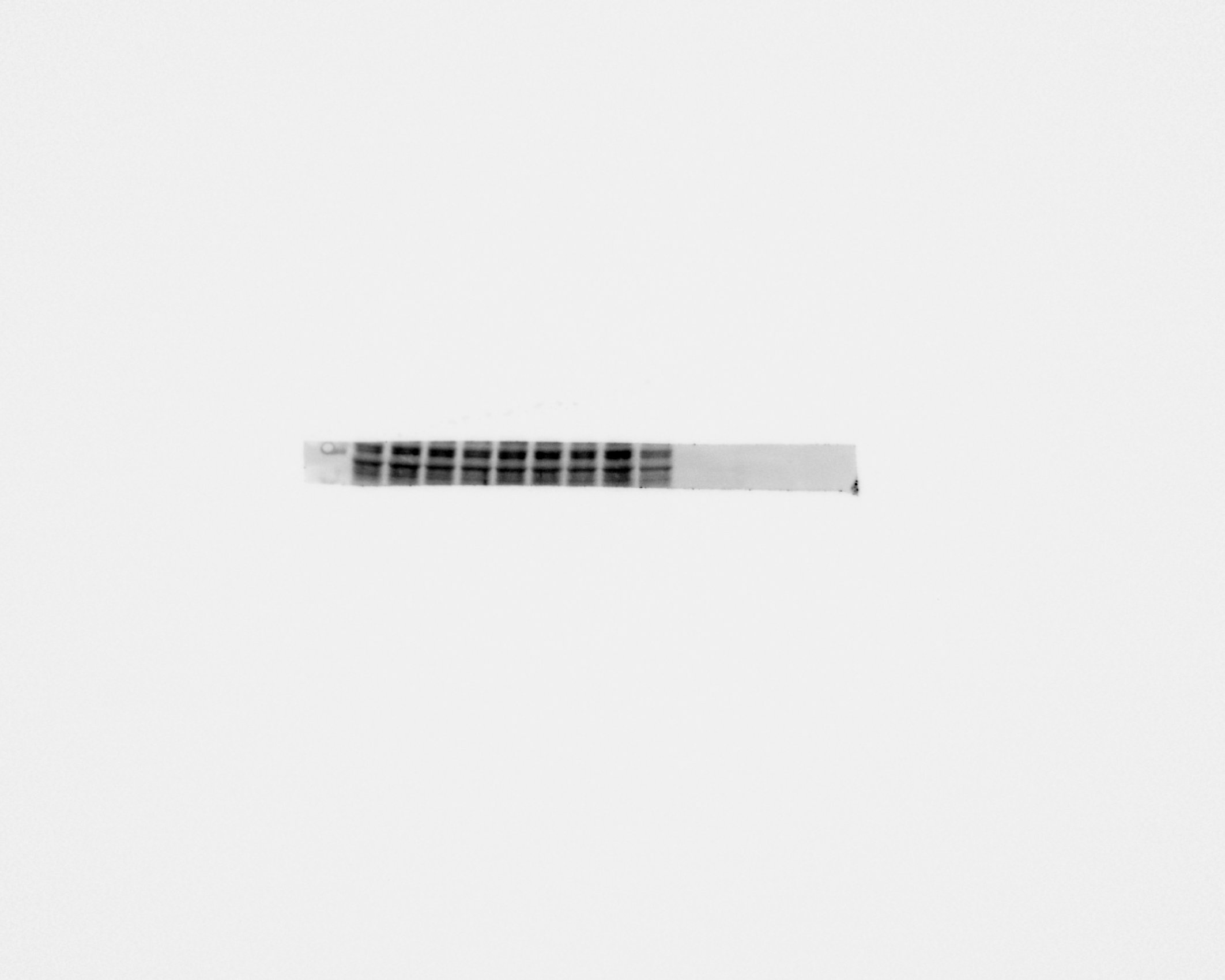

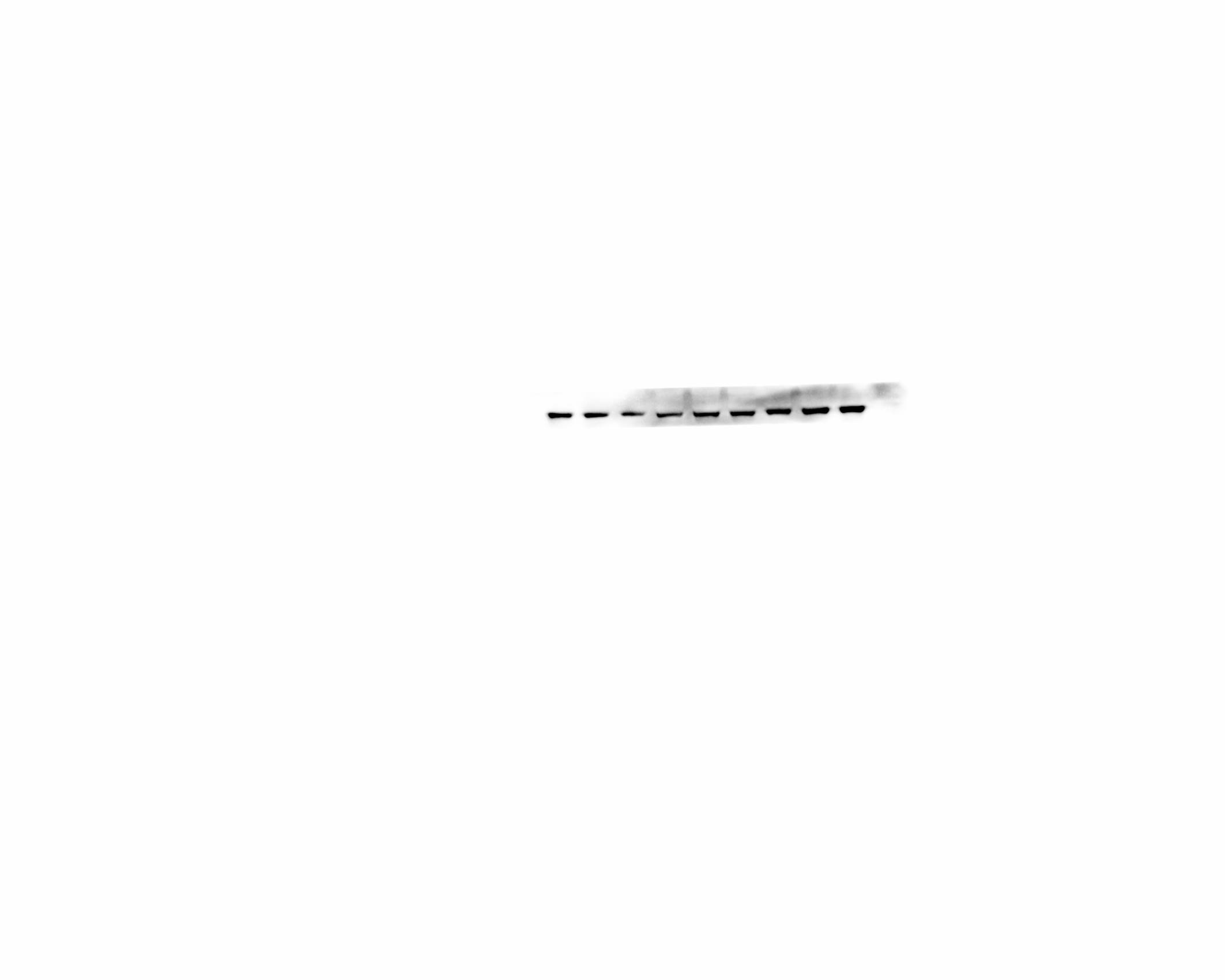


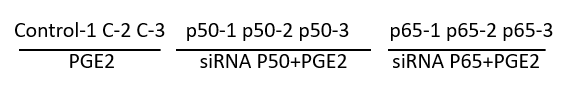


PKA NLRP3

NF-Kb p65 ASC

Caspase-1 IL-1β

β-Tubulin

**FigureS2.** Original blots of COX-1/COX2 and β-Tubulin of WT,5xFAD and 5xFAD/COX-1KO mice or isolated microglia.

COX-1 β-Tubulin

COX1 COX2 Tubulin-1

COX2 Tubulin-1
